# Enhancement of protein production via the strong *DIT1* terminator and two RNA-binding proteins in *Saccharomyces cerevisiae*

**DOI:** 10.1038/srep36997

**Published:** 2016-11-15

**Authors:** Yoichiro Ito, Takao Kitagawa, Mamoru Yamanishi, Satoshi Katahira, Shingo Izawa, Kenji Irie, Makoto Furutani-Seiki, Takashi Matsuyama

**Affiliations:** 1 Toyota Central R&D Labs. Inc., Nagakute, Aichi 480-1192, Japan; 2 Systems Biochemistry in Pathology and Regeneration, Yamaguchi University Graduate School of Medicine, Ube, Yamaguchi 755-8505, Japan; 3 Laboratory of Microbial Technology, Graduate School of Science and Technology, Kyoto Institute of Technology, Kyoto 606-8585, Japan; 4 Laboratory of Molecular Cell Biology, Faculty of Medicine, University of Tsukuba, Tsukuba, Ibaraki 305-8575, Japan

## Abstract

Post-transcriptional upregulation is an effective way to increase the expression of transgenes and thus maximize the yields of target chemicals from metabolically engineered organisms. Refractory elements in the 3′ untranslated region (UTR) that increase mRNA half-life might be available. In *Saccharomyces cerevisiae*, several terminator regions have shown activity in increasing the production of proteins by upstream coding genes; among these terminators the *DIT1* terminator has the highest activity. Here, we found in *Saccharomyces cerevisiae* that two resident *trans*-acting RNA-binding proteins (Nab6p and Pap1p) enhance the activity of the *DIT1* terminator through the *cis* element GUUCG/U within the 3′-UTR. These two RNA-binding proteins could upregulate a battery of cell-wall–related genes. Mutagenesis of the *DIT1* terminator improved its activity by a maximum of 500% of that of the standard *PGK1* terminator. Further understanding and improvement of this system will facilitate inexpensive and stable production of complicated organism-derived drugs worldwide.

Strong expression of the enzyme-encoding genes involved in target metabolic pathways is essential for the efficient production of target chemicals in the cells of metabolically engineered microorganisms, where multiple reactions proceed simultaneously. In *Saccharomyces cerevisiae*, this goal typically is achieved through upregulation at the transcriptional level, such as by incorporating strong promoters and multiple copies of transgenes^1^. A successful example of this strategy is the transgenic *S. cerevisiae* strain that produces the precursor for the antimalarial drug artemisinin^2^. Specifically, the *GAL1/10* promoter—one of the strongest promoters in *S. cerevisiae*^3^—was used in combination with the multi-copy 2μ vector to strongly express several genes encoding enzymes involved in the synthesis of the isoprenoid^2^. However, upregulation at the transcriptional level comes with the burden of mRNA turnover and the accompanying energy consumption. The greater the increase in the number of transgenes to be introduced, the greater the reduction in yield of the target chemical. Therefore, alternative means that upregulate gene expression at the post-transcriptional level are required for the efficient production of complicated organism-derived drugs, such as opioids^4^.

Terminator regions are located within about 200 bp downstream of protein-coding sequences and have distinct functions, namely transcriptional termination, poly(A) addition, and post-transcriptional regulation via the 3′-UTR^5^,^6^. The amount of protein accumulated depends upon mRNA stability, which might in turn depend on secondary structures in the 3′-UTR^7^. Refractory elements in the 3′ untranslated region (UTR) that increase mRNA half-life might be available. In *Saccharomyces cerevisiae*, several terminator regions have shown activity in increasing the production of proteins by upstream coding genes^8^,^9^,^10^,^11^,^12^. Whereas some *cis*-elements within the 3′-UTR and its corresponding *trans* proteins downregulate gene expression via mRNA degradation or translational repression^13^,^14^, in vertebrates a few promote the accumulation of protein through translation initiation by cytoplasmic polyadenylation^15^, stabilization of fragile mRNA^16^, or translational activation^17^,^18^ by formation of mRNA–protein complexes. These elaborate mechanisms, which function transiently during developmental process or stress responses, have yet to be applied in metabolic engineering.

Among the terminator regions in the *S. cerevisiae* genome, the dityrosine-deficient 1 (*DIT1*) terminator (*DIT1t*), which was identified by comprehensive screening^9^, has the strongest ability to increase transgene expression under various growth conditions and among various *S. cerevisiae* strains^19^. The *DIT1* terminator also upregulates the expression of upstream transgenes that encode various proteins, including fluorescent proteins and cellulases, under different promoters^19^,^20^. Only the activity of *DIT1t* is enhanced in the stationary phase, whereas those of the other top-five-ranked terminators decrease, suggesting that *DIT1t* activity is post-transcriptionally upregulated by unknown factors^19^, which might interact with the *DIT1* 3′-UTR (Fig. 1A). In this study, we identified these factors to improve the strong *DIT1* terminator for protein production and metabolic engineering.

## Results and Discussion

### Identification of *DIT1t*-activating genes

**Figure 1:**
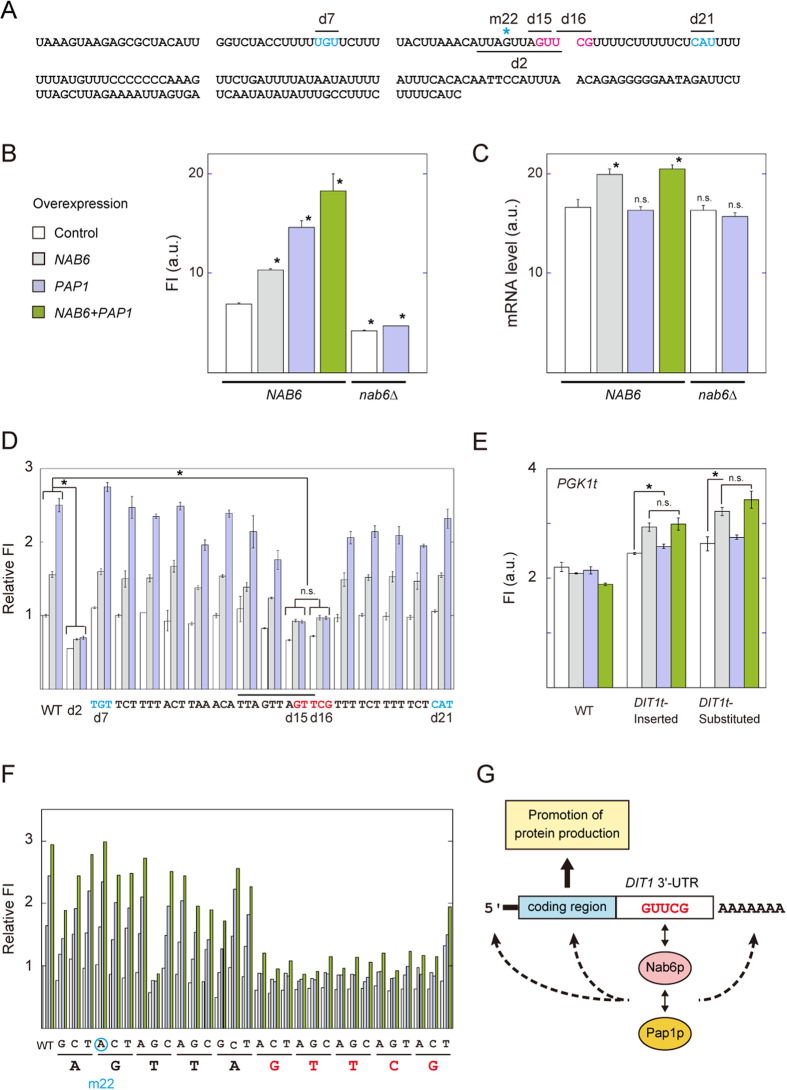
Identification of *DIT1t*-activating factors. (**A**) Sequence of the *DIT1* 3′-UTR. The length is 208 bp. The *cis*-element GUUCG is indicated in red. Five deleted regions are indicated by lines. One point mutation (m22) is indicated by an asterisk. Two effective deletion mutations (d7 and d21) are indicated in cyan. (**B**) Genetic interaction between *PAP1* and *NAB6*. The GFP fluorescence intensity of the control *NAB6* strain harboring pGP564 was used as the standard. (**C**) GFP mRNA levels analyzed by RT-PCR. Each total RNA was extracted from the corresponding cells, as denoted in the caption to Fig. 1B. (**D**) Deletion analysis of the *DIT1* terminator to identify Pap1p- and Nab6p-recruiting *cis* region(s). A series of *DIT1t* mutants (d7 to d21) with 3-bp deletions in the *DIT1t* region (Supplementary Table 3) and a 10-bp-deleted d2 mutant were constructed. The GFP fluorescence intensity of the wild-type DIT1t strain harboring pGP564 was used as the standard control (indicated as “WT”). A d2-deleted region is indicated by the line. Four deleted regions (d7, d15, d16, and d21) are indicated. (**E**) Gain-of-function analyses using mutated *PGK1* terminators. Two strains harboring a mutated terminator (Supplementary Fig. 7) were constructed. The GFP fluorescence intensities were measured. (**F**) Identification of *DIT1t*-activating *cis*-element sequences by using point mutagenesis. A series of *DIT1t* mutants (m1 to m30) with point mutations in the *DIT1t* region (Supplementary Table 3) were constructed. One point mutation (m22) is indicated by a cyan circle. The GFP fluorescence intensity of the wild-type DIT1t strain harboring pGP564 was used as the standard. Data are means of three or four independent experiments. (**G**) The protein production system involving the *cis* element GUUCG in the *DIT1* 3′-UTR. Both Nab6p and Pap1p are considered to be *trans*-acting factors in this system. The empty control vector (white), pGP564 with *NAB6* insert (gray), pGP564 with *PAP1* insert (blue), and pGP564 with *PAP1*–*NAB6* combined insert (green) were separately transformed into the corresponding strains. Data are means ± 1 SD of at least three independent experiments. n.s., not significant; *p < 0.01. See also Supplementary Table 5.

To shed light on these unknown *DIT1t*-activating factors, we introduced the Yeast Genomic Tiling Collection^21^ into a DIT1t strain that had a genome-integrated green fluorescent protein (GFP) upstream of *DIT1t* under the control of the *TDH3* gene promoter (Supplementary Figs 1–3 and Supplementary Table 1). We identified two *DIT1t*-activating genes: nucleic acid-binding protein 6^22^ (*NAB6*), which had one RNA-binding domain and three poly(A) binding domains, and poly(A) RNA polymerase^23^ (*PAP1*). Each gene enhanced the fluorescence intensity of GFP upon overexpression in the DIT1t strain (Fig. 1B), but not in the other top-five-ranked strains (Supplementary Fig. 4). A transformant overexpressing both *NAB6* and *PAP1* in the DIT1t strain exhibited greater GFP fluorescence than did the transformants harboring each gene separately. Deletion of *NAB6 (nab6*Δ) in the DIT1t strain background decreased the GFP fluorescence to 60% and virtually prevented *PAP1* overexpression from increasing GFP fluorescence (Fig. 1B). In contrast, no significant differences in fluorescence intensity between the *NAB6* background and the *nab6*Δ background were observed in the other terminator backgrounds (Supplementary Fig. 5). These results indicated that *NAB6* and *PAP1* increased *DIT1t* activity specifically and coordinately. In taking these results together with the physical interaction between Nab6p and Pap1p^24^, we hypothesized that Nab6p might preferentially bind with the *DIT1* 3′-UTR of mRNA and recruit Pap1p to sterically inhibit the degradation of the mRNA. Alternatively, RNA polymerase II-dependent gene looping^25^ between the *DIT1* terminator and the cognate promoter via a Nab6p–Pap1p complex might facilitate transcription reinitiation.

### mRNA level

To evaluate these two possibilities, we conducted quantitative PCR to investigate the amounts of GFP mRNA containing the *DIT1* 3′-UTR that were produced by these strains (Fig. 1C). Overexpression of *NAB6* increased the amount of GFP mRNA by 10% independently of *PAP1*, whereas the GFP mRNA level was not affected by the *PAP1*-overexpressing or *nab6*Δ background. These results indicated that gene looping likely does not promote transcription of the transgenes harboring *DIT1t*. These results raise the possibility that Nab6p binds to the *DIT1* 3′-UTR and thus stabilizes the mRNA to increase protein yield; however, the contribution of Pap1p to the increase strongly suggests a mechanism other than mRNA stabilization.

### Identification of *cis*-element

To genetically identify the *cis*-elements associated with *DIT1t* activation, we made six *DIT1t* mutants with 10-bp deletions (d1 to d6) from the 208-bp sequence of the wild-type *DIT1* 3′-UTR, as determined by 3′-RACE (Supplementary Fig. 6A). Of the six deletions, only the d2 deletion (i.e., UUAGUUAGUU [+51 to +60 from the stop codon]; Fig. 1D and Supplementary Fig. 6B) prevented both *NAB6* and *PAP1* overexpression from increasing *DIT1t* activity. This result suggested that a specific *cis*-element was present near the d2 deletion. As a result of comprehensive comparative analyses of diverse eukaryotic genomes, the length of the *cis*-element within the 3′-UTR is considered to be about 5 to 10 bp^26^. To conduct fine mapping of the *cis*-element, we made another series of *DIT1t* mutants with 3-bp deletions between regions d1 and d3 (d7 to d21). Of the 15 deletions, two consecutive ones, d15 (AGT [+57 to +59]) and d16 (TCG [+60 to +62]) prevented the activation of *DIT1t* by overexpression of both *NAB6* and *PAP1* (Fig. 1D). These deleted regions overlapped with the d2 deletion, suggesting that AGUUCG was the *cis*-element involved in *DIT1t* activation. Gain-of-function analyses using mutated *PGK1* terminators with the AGTTCG insertion or substitution showed that the *cis*-element might increase terminator activity by overexpression of *NAB6* (Fig. 1E and Supplementary Fig. 7). Overexpression of *PAP1* has not been observed to significantly increase the fluorescence intensity of GFP (Fig. 1E). Saturation mutagenesis with 1-bp mutations around the core candidate sequence was conducted to determine the functional *cis*-element sequences (Fig. 1F). Among the mutated sequences shown in this figure, substitution of any of the first five nucleotides only slightly reduced DIT1t activation compared with that in the wild type, although substitution of the first T for a G reduced activation more markedly. Substitution of the last five nucleotides markedly reduced the degree of activation, whereas substitution of the last G with a T made *DIT1t* activation only slightly lower than in the wild type. Taken together, the *cis*-element GUUCG/U in the 3′-UTR might enhance upstream protein production in a way that is dependent upon Nab6p and Pap1p (Fig. 1G). Pap1p might modulate the length of the poly(A) tail of the mRNA harboring the *DIT1* 3′-UTR to increase translation. Alternatively, an Nab6p–Pap1p complex directly interacts with the initiation factor complex to increase the frequency of translation initiation or to stabilize polyribosomes to promote translation.

### Other target mRNAs

In eukaryotes, RNA-binding-protein–mRNA complexes have been proposed to function as hubs to regulate genetic networks at the posttranscriptional level but remains to be well elucidated^26^,^27^. The mRNAs associated with Nab6p have been identified comprehensively and the significance of the interactions ranked whereas the sequence of the Nab6p binding motif has yet to be determined^27^. *DIT1* mRNA was not detected as an Nab6p-binding mRNA in the previous study^27^; this could have been because *DIT1* is expressed strictly at a specific time during sporulation^28^. We investigated whether the terminators from Nab6-binding mRNA genes were activated by overexpression of *NAB6* and *PAP1* in the same way as was *DIT1t* (Table 1). Eleven of the top-18-ranked terminators exhibited Nab6p–Pap1p-dependent activation, and all 11 terminator regions had at least one core *cis*-element sequence, GUUCG/U, within 200-bp downstream from the stop codon, as was found in *DIT1t* (Table 1). Nab6p–Pap1p-dependent activation appeared irrelevant to the intensity of terminator activity (Table 1). Six of the 11 terminators were derived from cell wall–related genes (Table 1). *DIT1* is also involved in the synthesis of spore walls^29^, suggesting that Nab6p–Pap1p–mRNA complexes could form part of the networks regulating cell-wall modification. This putative network could also regulate the other cell wall-related genes. These results also support the hypothesis that Nab6p directly associates with the *cis*-element GUUCG/U within the 3′-UTR.

### Effects on cell growth

**Figure 2:**
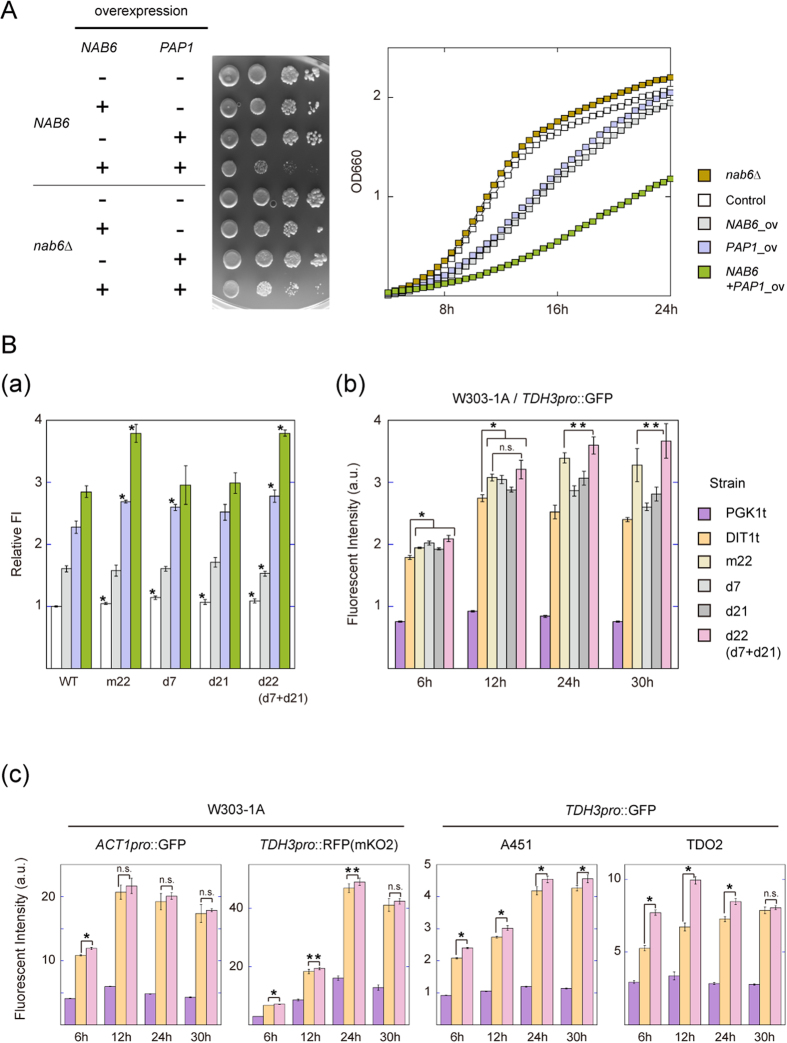
(**A**) Growth retardation by overexpression of *NAB6* and *PAP1*. (Left) Empty pGP564 vector, pGP564-NAB6, pGP564-PAP1, and pGP564-PAP1-NAB6 were separately transformed into the wild-type W303-1A strain (upper; marked *NAB6*) or the *nab6*Δ strain (lower; marked *nab6*Δ). Two days after inoculation on SD-leucine medium at 30 °C, the colonies were observed. (Right) Optical density (OD) was measured every 30 min from 0 to 24 h after reinoculation at OD_660_ = 0.1. The averages of three replicate experiments from 6 to 24 h are indicated. The *nab6*Δ/DIT1t strain (gold) and the *NAB6*/DIT1t strains harboring pGP564 (white), pGP564-NAB6 (gray), pGP564-NAB6 (blue), or pGP564-PAP1-NAB6 (green) are indicated. (**B**) Identification of the strongest terminator derived from *DIT1t*. (a) Activation of strong *DIT1t*-derived terminators by overexpression of *NAB6* and *PAP1*. The fluorescence intensity of the wild-type DIT1t strain harboring pGP564 was used as the standard. These experiments were conducted independently of those in Fig. 1. (b) Comparison of terminator activities among DIT1t strains and the standard PGK1t strain in various growth phases. The fluorescence intensities of terminator strains with the GFP gene under the control of the *TDH3* promoter were measured by flow cytometry. Sampling times were 6, 12, 24, and 30 h after reinoculation at OD_660_ = 0.1. (c) Characterization of the strongest *DIT1t*-d22 terminator. (Left) Effects of promoter and reporter gene exchange on 3′-UTR activity. These strains expressed either the GFP gene under the control of *ACT1pro* (left) or the mKO2 gene under the control of *TDH3pro* (right), as indicated. (Right) Effect of the host strain on 3′-UTR activity. Two wild-type *Saccharomyces cerevisiae* strains (A451 and TDO2) were transformed individually. The fluorescence intensities of the terminator strains containing the GFP gene under the control of the *TDH3* promoter were measured by flow cytometry. The terminator strains examined were PGK1t (purple), DIT1t (orange). DIT1t-m22 (yellow), DIT1t-d7 (gray), DIT1t-d21 (dark gray), and *DIT1t*-d22 (pink). Data are means ± 1 SD of at least three independent experiments. n.s., not significant; *p < 0.01; **p < 0.05. See also Supplementary Table 5.

In batch fermentation, both the rate of production and the overall product yield often are correlated with growth rate. We investigated whether the deletion of *NAB6* or overexpression of *NAB6* or *PAP1* influenced the growth rates of the yeasts in the absence of the expression of other transgenes upstream of *DIT1t*. At 30 °C, the growth of the *nab6*Δ mutant was comparable to that of the control strain. In contrast, strains overexpressing either *NAB6* or *PAP1* showed decreased growth compared with the control strain, and the strain overexpressing both *NAB6* and *PAP1* exhibited even greater growth retardation than did either of the single protein–overexpressing strains (Fig. 2A). This detrimental effect could have resulted from exacerbation of *NAB6* and *PAP1* gene expression by the action of the Nab6p–Pap1p complex, followed by disordered composition, or modification, of the cell wall. Overexpression of cell wall–related genes decreases growth rates during the vegetative stage in *S. cerevisiae*^30^. Excess Nab6p could non-specifically bind poly(A) within mRNAs via its poly(A) binding domains to indiscriminately affect gene expression. In metabolically engineering yeasts, we must design the elements in such a way that specifically increases the expression of the target enzyme genes. Our result here implied that directly overexpression of *NAB6* and/or *PAP1* was inappropriate as a means of enhancing the expression of transgenes.

### Improvement of the *DIT1* terminator

To improve this post-transcriptional expression system without overexpressing *NAB6* and *PAP1*, we modified the sequence of the *DIT1* 3′-UTR. We found that three mutated *DIT1* terminators had 5% to 10% greater activity than the wild-type *DIT1t* when *NAB6* and *PAP1* were overexpressed (Fig. 2B (a)). The activity of *DIT1t*-d22, which was made by accumulation of the d7 and d21 mutations, was greater than those of *DIT1t*-d7 and *DIT1t*-d21 and comparable to that of *DIT1t*-m22. Among five *DIT1t*-derived terminators, *DIT1t*-d22 had the greatest activity from the early log phase to the stationary phase in the W303-1a background (Fig. 2B (b)). During the stationary phase, the maximum activity of *DIT1t*-d22 was about 5 times that of the standard *PGK1* terminator and 1.5 times that of the wild-type *DIT1* terminator. We examined the activities of both *DIT1t* and *DIT1t*-d22 in terms of the effects of promoter and reporter gene exchanges, namely by using the weak *ACT1* promoter instead of the strong *TDH3* promoter and by using an mKO2 red fluorescent protein gene instead of the GFP gene (Left panel in Fig. 2B (c)). The *DIT1t*-d22 strain showed greater terminator activity than the *DIT1t* strain while a significant difference was observed only at log phase in both cases. In another haploid strains (A451) and one diploid strain (TDO2) used in the abovementioned study^19^ and transfected with constructs harboring *DIT1t* or *DIT1t*-d22, we observed that transfection with the *DIT1t*-d22 strain gave significantly greater terminator activity than did transfection with the wild-type *DIT1t* strain at almost all phases (Right panel in Fig. 2B (c)).

## Conclusions

Like in the case of opioid-producing yeasts with dozens of transgenes^4^, use of our system might improve the efficiency of production of target chemicals. The molecular mechanism by which Nab6p–Pap1p–3′-UTR complexes increase protein production by transgenes remains to be determined. Two mutated *PGK1* terminators were associated with an increase in upstream protein production in a manner dependent on Nab6p but not on Pap1p (Fig. 1E). These results suggested that *cis-*elements other than GUUCG/U are needed for Pap1p to interact with Nab6p or the 3′-UTR to enhance protein production. Further investigation is needed to determine all of the components of this mechanism and their functions. With such knowledge, we would be able to improve this regulatory system to increase protein production by transgenes and apply it to other organisms by using synthetic biological methods. The use of these methods likely will contribute to the development of metabolically-engineered organisms for producing a wide variety of beneficial chemicals.

## Materials and Methods

### Host strain and media

*Escherichia coli* JM109 was used as the host cell for DNA manipulation. Luria–Bertani (LB) medium supplemented with 100 mg/L ampicillin or 30 mg/L kanamycin was used for *E. coli* cell culture to select the transformants. *Saccharomyces cerevisiae* strain BY4741 (*MAT***a** *URA3*Δ0 *LEU2*Δ0 *HIS3*Δ1 *MET15*Δ0) was used for screening and cloning. Strains expressing codon-optimized GFP under the control of the *TDH3* promoter and either *DIT1t (DIT1t* strain), the *RPL15BA* terminator (*RPL15BAt* strain), the *RPL41B* terminator (*RPL41B* strain), the *RPL3* terminator (*RPL3t* strain), the *IDP1* terminator (*IDP1t* strain), or the *PGK1* terminator (*PGK1t* strain) were constructed previously^19^. *Saccharomyces cerevisiae* strain W303-1A (*MATa leu2-3, 112 trp1-1 can1-100 ura3-1 ade2-1 his3-11, 15*) was also used as a host strain for genetic analyses. Three *S. cerevisiae* strains were used as wild-type strains to examine the activity of *DITt*-d22: they were A451 (*MAT*α *can1 leu2 trp1 ura3 aro7*); TDO2 (*MAT*a/α *trp1/trp1 ura3/ura3*) made from OC-2T^31^ with the use of 5-fluoroorotic acid; and YPH499 (*MAT*a *ura3-52 lys2-801 ade2-101 trp1*-Δ63 *his3*-Δ200 *leu2*-Δ1). Yeast cells were grown in synthetic complete medium, which contained 0.67% yeast nitrogen base without amino acids (Difco, Detroit, MI), 0.082% complete supplement mixture (CSM), CSM-Leucine (ForMedium, Norfolk, UK), and adenine (40 mg/L), supplemented with 2% glucose (Synthetic Dextrose minimal medium; SD) and with 2% agar as needed.

### Plasmid construction

Except in the case of the pGP564-PAP1-NAB6 vector, all plasmid construction was performed by using the gap-repair cloning method^32^ and the BY4741 strain. For each gene, the region from the predicted promoter region to the terminator region was cloned into the pGP564 vector (#YSC5034, Thermo Fisher Scientific Inc., MA). The pGP564 vector was digested with *Bam*HI and *Xho*I restriction enzymes at 37 °C to prepare linear DNA for gap-repair cloning. The genes used in this study are as follows, and the locations of the regions amplified are given relative to the start codon of the gene (=position 1). NAB6 (−391 to +3952) and YML119W (−1000 to +1720) were amplified from 12B3 vector. PAP1 (−509 to +2149) and ECM9 (−746 to +1312) were amplified from 10A10 vector. CCC1 (−487 to +1540) was amplified from 11E10 vector. MSC3 (−500 to +2674) was amplified from the BY4704 strain chromosome. Plasmids pGP564-NAB6, -YML119W, -PAP1, -ECM9, -CCC1, and -MSC3 were extracted from the transformants and then used to transform the JM109 strain, as described above. The pGP564 vector containing both the *PAP1* and *NAB6* gene sequences (pGP564-PAP1-NAB6) was constructed by using an In-Fusion HD cloning kit (Takara Bio Inc.). After the digestion of pGP564-NAB6 vector with *Spe*I restriction enzyme, the *PAP1* PCR product was reacted with the digested vector to produce the pGP564-PAP1-NAB6 vector.

### Screening for *DIT1t*-activating factors by using the Yeast Genomic Tiling Collection

A simple scheme of the screening strategy is given in Supplementary Fig. 1. The Yeast Genomic Tiling Collection, which was constructed previously^21^, was purchased from Thermo Fisher Scientific (catalog no. YSC4613). This collection is stored in 16 wells of a 96-well microplate. For each well, we mixed the *Escherichia coli* Yeast Genomic Tiling Collection strains together and cultured them at 37 °C overnight in 100 mL of LB medium containing 30 μg/mL kanamycin. The DNA of each of the 16 plasmid pools was then isolated by using the alkali-sodium dodecyl sulfate (SDS) method without the addition of RNase and used to transform the DIT1t strain. Yeast transformation was performed as described previously^33^. Briefly, yeast cells were grown in yeast extract peptone dextrose (YPD) medium overnight, then diluted 10-fold with YPD and incubated for 5 h. Cells were precipitated and washed with deionized water, and then mixed with transformation solution (120 μL of 60% PEG3350, 5 μL of 4 M lithium acetate, 10 μL of 1 M DTT, and 10 μL of 10 mg/mL denatured salmon sperm DNA) and the plasmid DNA. The mixture was incubated at 42 °C for 40 min and then spread on SD-leucine medium. The resultant pool of transformants was transiently collected and stored at −80 °C until further use. The pool of transformants was spread on SD-leucine medium, and individual transformants were randomly picked into 96-deep well plates and incubated at 30 °C at 1800 rpm in an MBR-022UP Bio Shaker Incubator (Taitec, Saitama, Japan) overnight. A 10-μL aliquot of culture was reinoculated into 500 μL of SD-leucine and grown at 30 °C for 6 h. The GFP fluorescence intensity (FI) and side-scatter intensity (SS) of the cells were measured by using a Cell Lab Quanta SC MPL flow cytometer (Beckman-Coulter, CA), and a histogram of the FI/SS ratio in each cell was fitted with IgorPro 6.1 software (Wavemetrics, Lake Oswego, OR), as described previously^34^.

### Construction of *nab6*Δ strain

*NAB6* gene deletion in the W303-1a strain (*nab6*Δ) was performed as follows. First, the KanMX selectable marker region was amplified from the genome of the faa4::KANMX4/faa4::KANMX4 strain in the diploid non-essential homozygous deletion strain collection (Research Genetics, Huntsville, AL). We then conducted nested PCR with NAB6-nested-f (5′-CCTTGTTTCAGGTTACGTGAAAAGCATCCAGAGAAGAT-3′) and NAB6-nested-c (5′-AAGAAATCGGAGAAAAAAAGGGAGAAACACCTTGGCAG-3′), followed by NAB6–150(40)-KANMX4-334 (5′-AAAGCATCCAGAGAAGATATCCCAAAGACTGAAGAGGTGTcccagaataccctccttgacag-3′) and NAB6+3500c(40)-KANMX4+1023c (5′-GGAGAAACACCTTGGCAGGCCGCCTTAGGCTTCCTTGGGCgaatcgacagcagtatagcgacca-3′). The PCR product was transformed into the DIT1t strain, and the resultant transformants were plated on YPD medium containing 100 μg/mL G418 to select for the presence of the KanMX marker.

### Analysis of genetic interaction between *NAB6* and *PAP1*

Four plasmids (pGP564, pGP564-NAB6, pGP564-PAP1, and pGP564-NAB6-PAP1) were introduced into the DIT1t strain in the W303-1a *NAB6* background. Two plasmids (pGP564 and pGP564-PAP1) were introduced into the DIT1t strain in the *nab6*Δ background. As described above, the intensities of GFP fluorescence of these six strains were investigated 6 h after reinoculation.

### Quantitative real-time PCR analysis

mRNA levels were measured as described in our previous paper^8^. Briefly, the DIT1t strain was cultured for 6, 12, or 24 h, and then total RNA was extracted by using a High Pure RNA Isolation Kit (Roche, Basel, Switzerland). cDNA was synthesized from a 1-μL aliquot of total RNA by using a High Capacity cDNA Reverse Transcription Kit (Life Technologies, Carlsbad, CA). Real-time PCR analysis was performed with SYBR Green PCR Master Mix and an ABI PRISM 7000 sequence detection system (Life Technologies). To quantify the amount of cDNA, the following primers were used: PAP1-1 (5′-TGGAGTGGTCTTGTTGAAAGTAAGG-3′), PAP1-2 (5′-AAGGGTTTGGTGAAAGGATGTG-3′), NAB6-1 (5′-TGGGTTCAGACATAGGCAATAGAAC-3′), NAB6-2 (5′-TCCTCCACGCACGACATTA-3′), GFP-1 (5′-CCAATTGGTGATGGTCCAGTCT-3′), GFP-2 (5′-CGGTGACGAACTCCAACAAAA-3′), TUB1-1 (5′-CCAACTGGTTTCAAGATCGGTA-3′), and TUB1-2 (5′-TCCACAGTGGCCAATTGTGA-3′). The amount of *TUB1* mRNA was used as an internal control.

### Identification of the sequence of the *DIT1* 3′-UTR

Rapid amplification of cDNA ends PCR (RACE-PCR) was used to identify the complete sequence of the *DIT1* 3′-UTR (3′-Full RACE Core set; Takara Bio Inc., Shiga, Japan). One microgram of total RNA isolated from the *DIT1t* strain was reverse-transcribed by using the oligo(dT) adaptor primer, and this first-strand cDNA was amplified with PrimeSTAR HS DNA polymerase (Takara Bio Inc.) by using an optimized GFP-specific primer (5′-CCAGACAACCATTATTTG-3′) and an anchor primer. Amplified 3′-RACE products were cloned into the pCR4Blunt-TOPO vector (Invitrogen); more than 20 independent clones were sequenced.

### Identification of the sequence of the *DIT1t*-activating *cis*-element

A series of DNA fragments containing 21 deletion-mutated terminators (d1 to d21) and 30 point-mutated terminators (m1 to m30) were synthesized (Genscript, Inc., Piscataway, NJ). These mutated *DIT1* terminators are listed in Supplementary Table 2. Strains expressing codon-optimized GFP under the control of the *TDH3* promoter and each *DIT1t (DIT1t* strains) were constructed as described above. Three plasmids (pGP564, pGP564-NAB6, or pGP564-PAP1) were introduced into the deletion-mutated DIT1t strains. Four plasmids (pGP564, pGP564-NAB6, pGP564-PAP1, or pGP564-NAB6-PAP1) were introduced into the point-mutated DIT1t strains. As described above, the intensities of GFP fluorescence of these 183 strains were investigated 6 h after reinoculation.

### Gain-of-function analyses using the mutated *PGK1* terminators

*DIT1t*-inserted *PGK1t* was designed as follows: the 17-bp *DIT1t* sequence containing a *cis*-element between +46 and +62 from the stop codon was inserted into the *PGK1* terminator between +28 and +29 from the stop codon (Supplementary Table 2 and Supplementary Fig. 7). The *DIT1t*-substituted *PGK1t* was designed as follows: the 11-bp *PGK1t* sequence between +17 and +29 from the stop codon was substituted for the 14-bp *DIT1t* sequence (Supplementary Table 2 and Supplementary Fig. 7). Four plasmids (pGP564, pGP564-NAB6, pGP564-PAP1, or pGP564-NAB6-PAP1) were introduced into the three *PGK1t (PGK1t, DIT1t*-inserted *PGK1t*, and *DIT1t*-substituted *PGK1t*) strains. As described above, the intensities of GFP fluorescence of these 12 strains were investigated 6 h after reinoculation.

### Assay of the terminator activities of Nab6p-binding mRNA genes

Eighteen terminator regions of Nab6p-binding mRNA genes were amplified by PCR as described previously^8^. All cloning procedures were performed with an In-Fusion Advantage PCR Cloning Kit (Clontech, Mountain View, CA). Each PCR primer set was designed as 5′-AATTGTATAACTGAGGTACC-3′ plus a gene-specific forward primer, and 5′-TAATGTCGTTGGATCC-3′ plus a gene-specific reverse primer. Each cloned terminator region was inserted into the vector and identified by sequencing analysis. DNA sequences of both the gene-specific primers and the amplified regions are listed in Supplementary Table 3. Each terminator strain was constructed, and the activity of the terminator of each strain was measured as described above (Supplementary Fig. 8).

### Assay of activities of mutation-accumulated *DIT1* terminators

A DNA fragment containing *DIT1t*-d22 was synthesized (Genscript, Inc., Piscataway, NJ). These DNA sequences are described in Supplementary Table 3. Four plasmids (pGP564, pGP564-NAB6, pGP564-PAP1, or pGP564-NAB6-PAP1) were introduced into the DIT1t-d22 strain. As described above, the intensities of GFP fluorescence were investigated 6 h after reinoculation.

## Additional Information

**How to cite this article**: Ito, Y. *et al*. Enhancement of protein production via the strong *DIT1* terminator and two RNA-binding proteins in *Saccharomyces cerevisiae. Sci. Rep.* **6**, 36997; doi: 10.1038/srep36997 (2016).

**Publisher’s note**: Springer Nature remains neutral with regard to jurisdictional claims in published maps and institutional affiliations.

## Supplementary Material

Supplementary Information

## Figures and Tables

**Table 1:** Activation of the terminators of Nab6p-binding mRNA genes by overexpression of *NAB6* and *PAP1*.

Gene	Activation by *NAB6* + *PAP1* (-fold)^a^	Candidate for *cis*-elements	Score^b^	Terminator activity^c^
ASP3*	1.3	GTTCt	9.1	2.28
PIR1*	1.5	GTTCt	7.0	0.26
*FMP52*	1.6	GTTCG	5.2	1.92
*MFA1*	1.9	GTTCG	4.5	0.49
SCW4*	1.6	GTTCG	4.4	2.30
TIR2*	1.6	GTTCG	4.3	1.85
*YDR133C*	1.5	GTTCG	4.3	--
*RPL26B*	-	--	3.8	1.30
*RPL26A*	-	GTTCG	3.7	0.95
HSP150*	1.5	GTTCt	3.7	0.33
		GTTCG		
*YLR285C-A*	-	--	3.6	0.09
*SUC2*	-	--	3.5	1.04
*GPB1*	1.3	GTTCG	3.4	0.17
YGP1*	1.6	GTTCG	3.2	0.99
*SUI1*	-	--	3.1	1.73
*YDL159W-A*	-	--	2.9	0.11
*DDR2*	1.6	GTTCG	2.7	0.34
*YPR053C*	-	--	2.6	--
*DIT1*	2.9	GTTCG	--	2.22

-, no significant activation (<1.2-fold; see Extended Data Fig. 8).

--, not detected.

*Cell wall–related genes (*Saccharomyces* Genome Database); http://www.yeastgenome.org/).

^a^Relative intensity of GFP fluorescence of each terminator strain harboring pGP564-NAB6-PAP1 compared with that of the corresponding terminator control strain harboring pGP564 vector.

^b^These scores are index values for the interaction between Nab6p and the corresponding mRNA (from ref. 24).

^c^Activity relative to that of the standard *PGK1* terminator (from ref. 2).

## References

[b1] Da Silva N. A. & Srikrishnan S. Introduction and expression of genes for metabolic engineering applications in Saccharomyces cerevisiae. FEMS Yeast Res. 12, 197–214 (2012). 22129153 10.1111/j.1567-1364.2011.00769.x

[b2] Paddon C. J. . High-level semi-synthetic production of the potent antimalarial artemisinin. Nature 496, 528–532 (2013). 23575629 10.1038/nature12051

[b3] Lohr D., Venkov P. & Zlatanova J. Transcriptional regulation in the yeast GAL gene family: a complex genetic network. FASEB J. 9, 777–787 (1995). 7601342 10.1096/fasebj.9.9.7601342

[b4] Galanie S., Thodey K., Trenchard I. J., Filsinger Interrante M. & Smolke C. D. Complete biosynthesis of opioids in yeast. Science 349, 1095–1100 (2015). 26272907 10.1126/science.aac9373 PMC4924617

[b5] van Helden J., del Olmo M. & Perez-Ortin J. E. Statistical analysis of yeast genomic downstream sequences reveals putative polyadenylation signals. Nucleic Acids Res. 28, 1000–1010 (2000). 10648794 10.1093/nar/28.4.1000 PMC102588

[b6] Kuersten S. & Goodwin E. The power of the 3′ UTR: translational control and development. Nat. Rev. Genet. 4, 626–637 (2003). 12897774 10.1038/nrg1125

[b7] Babiskin A. H. & Smolke C. D. A synthetic library of RNA control modules for predictable tuning of gene expression in yeast. Mol. Syst. Biol. 7, 471 (2011). 21364573 10.1038/msb.2011.4 PMC3094065

[b8] Yamanishi M., Katahira S. & Matsuyama T. *TPS1* terminator increases mRNA and protein yield in a *Saccharomyces cerevisiae* expression system. Biosci. Biotechnol. Biochem. 75, 2234–2236 (2011). 22056446 10.1271/bbb.110246

[b9] Yamanishi M. . A Genome-Wide Activity Assessment of Terminator Regions in Saccharomyces cerevisiae Provides a “Terminatome” Toolbox. ACS Synth. Biol. 2, 337–347 (2013). 23654277 10.1021/sb300116y

[b10] Curran K. A., Karim A. S., Gupta A. & Alper H. S. Use of expression-enhancing terminators in Saccharomyces cerevisiae to increase mRNA half-life and improve gene expression control for metabolic engineering applications. Metab. Eng. 19, 88–97 (2013). 23856240 10.1016/j.ymben.2013.07.001 PMC3769427

[b11] Curran K. A. . Short Synthetic Terminators for Improved Heterologous Gene Expression in Yeast. ACS Synth. Biol. 4, 824–832 (2015). 25686303 10.1021/sb5003357

[b12] Ito Y., Yamanishi M., Ikeuchi A., Imamura C. & Matsuyama T. Combinatorial Screening for Transgenic Yeasts with High Cellulase Activities in Combination with a Tunable Expression System. PloS one 10, e0144870 (2015). 26692026 10.1371/journal.pone.0144870 PMC4687128

[b13] Parker R. RNA degradation in Saccharomyces cerevisae. Genetics 191, 671–702 (2012). 22785621 10.1534/genetics.111.137265 PMC3389967

[b14] Miller M. A. & Olivas W. M. Roles of Puf proteins in mRNA degradation and translation. Wiley Interdiscip. Rev. RNA 2, 471–492 (2011). 21957038 10.1002/wrna.69

[b15] Barnard D. C., Ryan K., Manley J. L. & Richter J. D. Symplekin and xGLD-2 are required for CPEB-mediated cytoplasmic polyadenylation. Cell 119, 641–651 (2004). 15550246 10.1016/j.cell.2004.10.029

[b16] Beckel-Mitchener A. C., Miera A., Keller R. & Perrone-Bizzozero N. I. Poly(A) tail length-dependent stabilization of GAP-43 mRNA by the RNA-binding protein HuD. J. Biol. Chem. 277, 27996–28002 (2002). 12034726 10.1074/jbc.M201982200

[b17] Uniacke J. . An oxygen-regulated switch in the protein synthesis machinery. Nature 486, 126–129 (2012). 22678294 10.1038/nature11055 PMC4974072

[b18] Fukao A. . MicroRNAs trigger dissociation of eIF4AI and eIF4AII from target mRNAs in humans. Mol. Cell 56, 79–89 (2014). 25280105 10.1016/j.molcel.2014.09.005

[b19] Ito Y. . Characterization of five terminator regions that increase the protein yield of a transgene in Saccharomyces cerevisiae. J. Biotechnol. 168, 486–492 (2013). 24126155 10.1016/j.jbiotec.2013.09.024

[b20] Ito Y., Yamanishi M., Ikeuchi A. & Matsuyama T. A highly tunable system for the simultaneous expression of multiple enzymes in Saccharomyces cerevisiae. ACS Synth. Biol. 4, 12–16 (2015). 24927017 10.1021/sb500096y

[b21] Jones G. M. . A systematic library for comprehensive overexpression screens in Saccharomyces cerevisiae. Nat. Methods 5, 239–241 (2008). 18246075 10.1038/nmeth.1181

[b22] Samanta M. P. & Liang S. Predicting protein functions from redundancies in large-scale protein interaction networks. Proc. Natl. Acad. Sci. USA 100, 12579–12583 (2003). 14566057 10.1073/pnas.2132527100 PMC240660

[b23] Lingner J., Kellermann J. & Keller W. Cloning and expression of the essential gene for poly(A) polymerase from S. cerevisiae. Nature 354, 496–498 (1991). 1840648 10.1038/354496a0

[b24] Ezeokonkwo C., Ghazy M. A., Zhelkovsky A., Yeh P. C. & Moore C. Novel interactions at the essential N-terminus of poly(A) polymerase that could regulate poly(A) addition in Saccharomyces cerevisiae. FEBS Lett. 586, 1173–1178 (2012). 22575652 10.1016/j.febslet.2012.03.036 PMC3587332

[b25] Ansari A. & Hampsey M. A role for the CPF 3′-end processing machinery in RNAP II-dependent gene looping. Genes Dev. 19, 2969–2978 (2005). 16319194 10.1101/gad.1362305 PMC1315401

[b26] Ray D. . A compendium of RNA-binding motifs for decoding gene regulation. Nature 499, 172–177 (2013). 23846655 10.1038/nature12311 PMC3929597

[b27] Hogan D. J., Riordan D. P., Gerber A. P., Herschlag D. & Brown P. O. Diverse RNA-binding proteins interact with functionally related sets of RNAs, suggesting an extensive regulatory system. PLoS Biol. 6, e255 (2008). 18959479 10.1371/journal.pbio.0060255 PMC2573929

[b28] Friesen H., Hepworth S. & Segall J. An Ssn6-Tup1-dependent negative regulatory element controls sporulation-specific expression of *DIT1* and *DIT2* in *Saccharomyces cerevisiae*. Mol. Cell. Biol. 17, 123–134 (1997). 8972192 10.1128/mcb.17.1.123 PMC231736

[b29] Briza P., Eckerstorfer M. & Breitenbach M. The sporulation-specific enzymes encoded by the *DIT1* and *DIT2* genes catalyze a two-step reaction leading to a soluble LL-dityrosine-containing precursor of the yeast spore wall. Proc. Natl. Acad. Sci. USA 91, 4524–4528 (1994). 8183942 10.1073/pnas.91.10.4524 PMC43818

[b30] Yoshikawa K. . Comprehensive phenotypic analysis of single-gene deletion and overexpression strains of Saccharomyces cerevisiae. Yeast 28, 349–361 (2011). 21341307 10.1002/yea.1843

[b31] Saitoh S., Mieno Y., Nagashima T., Kumagai C. & Kitamoto K. Breeding of a new type of baker’s yeast by δ-integration for overproduction of glucoamylase using a homothallic yeast. J. Ferment. Bioeng. 81, 98–103 (1996).

[b32] Ma H., Kunes S., Schatz P. J. & Botstein D. Plasmid construction by homologous recombination in yeast. Gene 58, 201–216 (1987). 2828185 10.1016/0378-1119(87)90376-3

[b33] Yamanishi M. & Matsuyama T. A modified Cre-lox genetic switch to dynamically control metabolic flow in Saccharomyces cerevisiae. ACS Synth. Biol. 1, 172–180 (2012). 23651155 10.1021/sb200017p

[b34] Matsuyama T., Yamanishi M. & Takahashi H. Improvement of galactose induction system in *Saccharomyces cerevisiae*. J. Biosci. Bioeng. 111, 175–177 (2011). 20947423 10.1016/j.jbiosc.2010.09.014

