# Supplementary material for: Enhancement of protein production via the strong DIT1 terminator and two RNA-binding proteins in Saccharomyces cerevisiae
Source: Sci Rep. 2016 Nov 15;6:36997. doi: 10.1038/srep36997 (PMC5109538; doi:10.1038/srep36997)
Supplement: Supplementary Information [file srep36997-s1.pdf]

Title:

Enhancement of protein production via the strong *DIT1* terminator and two RNA-binding proteins in *Saccharomyces cerevisiae*

Authors:

Yoichiro Ito, Takao Kitagawa, Mamoru Yamanishi, Satoshi Katahira, Shingo Izawa, Kenji Irie, Makoto Furutani-Seiki and Takashi Matsuyama

Supplementary Figures S1-S8 and Tables S1-S5.

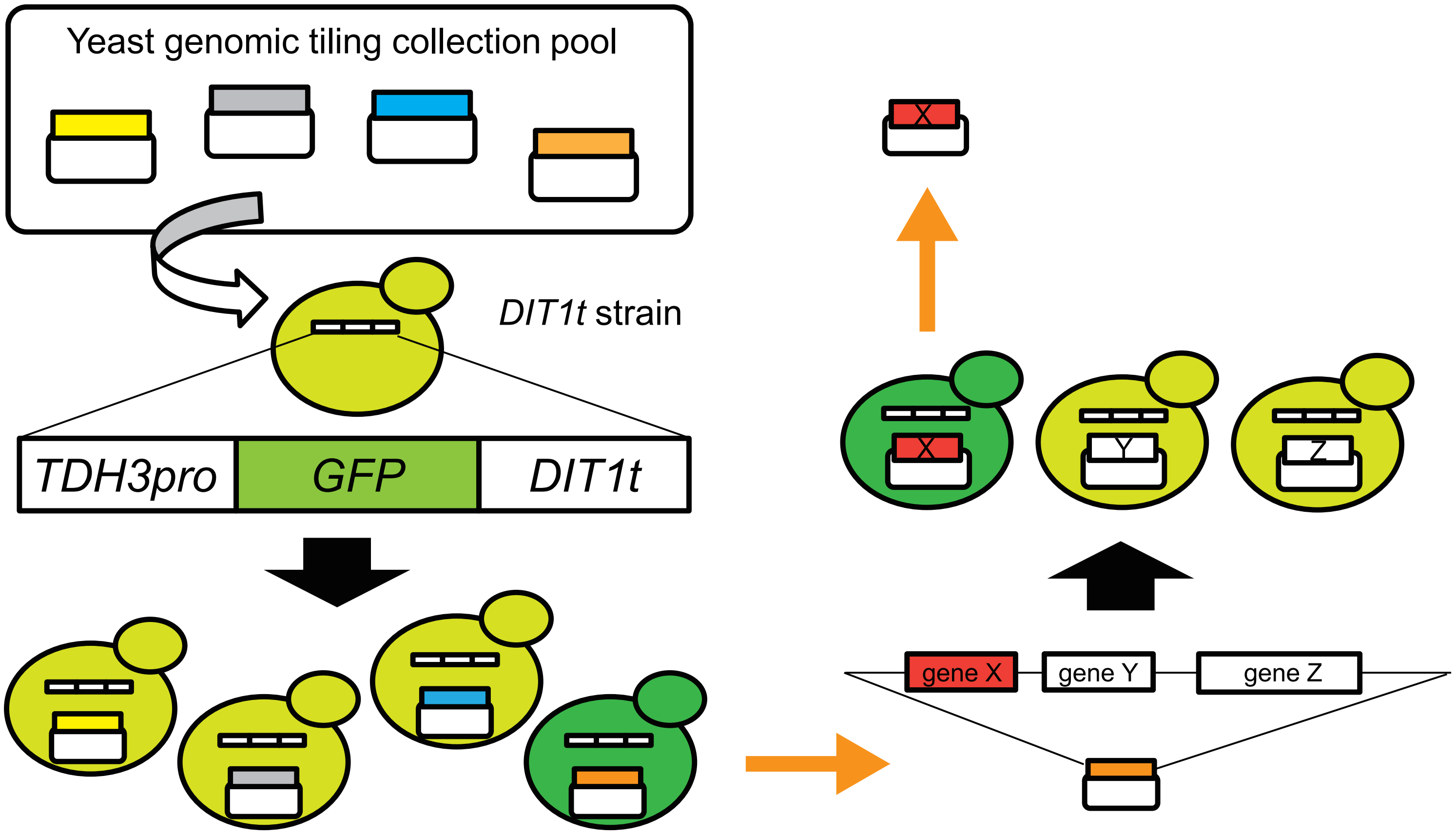

Supplymentary Figure S1. Scheme of screening for *DIT1t*-activating factors by using the Yeast Genomic Tiling Collection. The DIT1t strain, which expresses GFP under the control of the *TDH3* promoter (*TDH3pro*) and the *DIT1* terminator (*DIT1t*), was transformed with plasmid DNAs isolated from the yeast genomic tiling collection. The GFP fluorescence intensity of the transformants was then analyzed by flow cytometry and transformants with the highest GFP fluorescence intensity (marked with grey shading) were selected for further study. Plasmid DNAs containing multiple genes (marked as genes X, Y, Z) were then isolated from these transformants, and individual candidate genes (e.g., gene X) were cloned by gap-repair complementation, resulting in identification of the enhancing genes for the *DIT1* terminator.

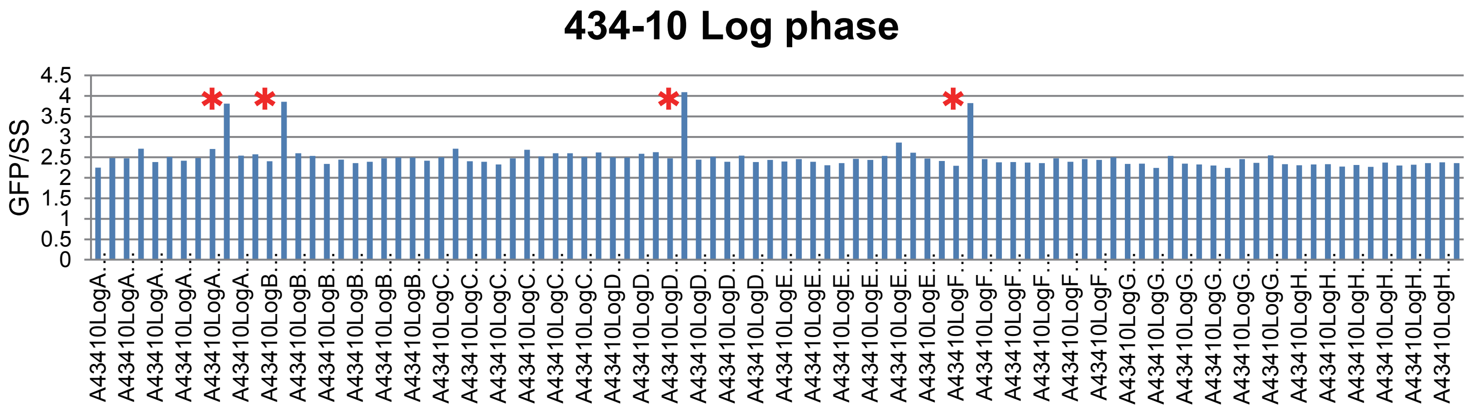

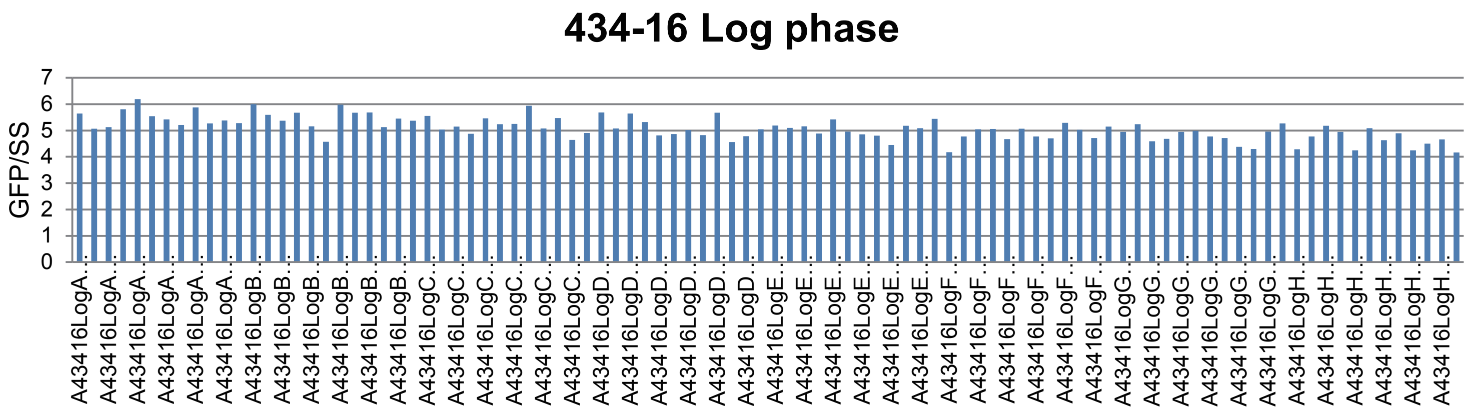

Supplementary Figure S2. Raw data used in the first screening. Candidate clones were located in the upper-row data set. No candidate clone was detected in the lower-row data set.

.

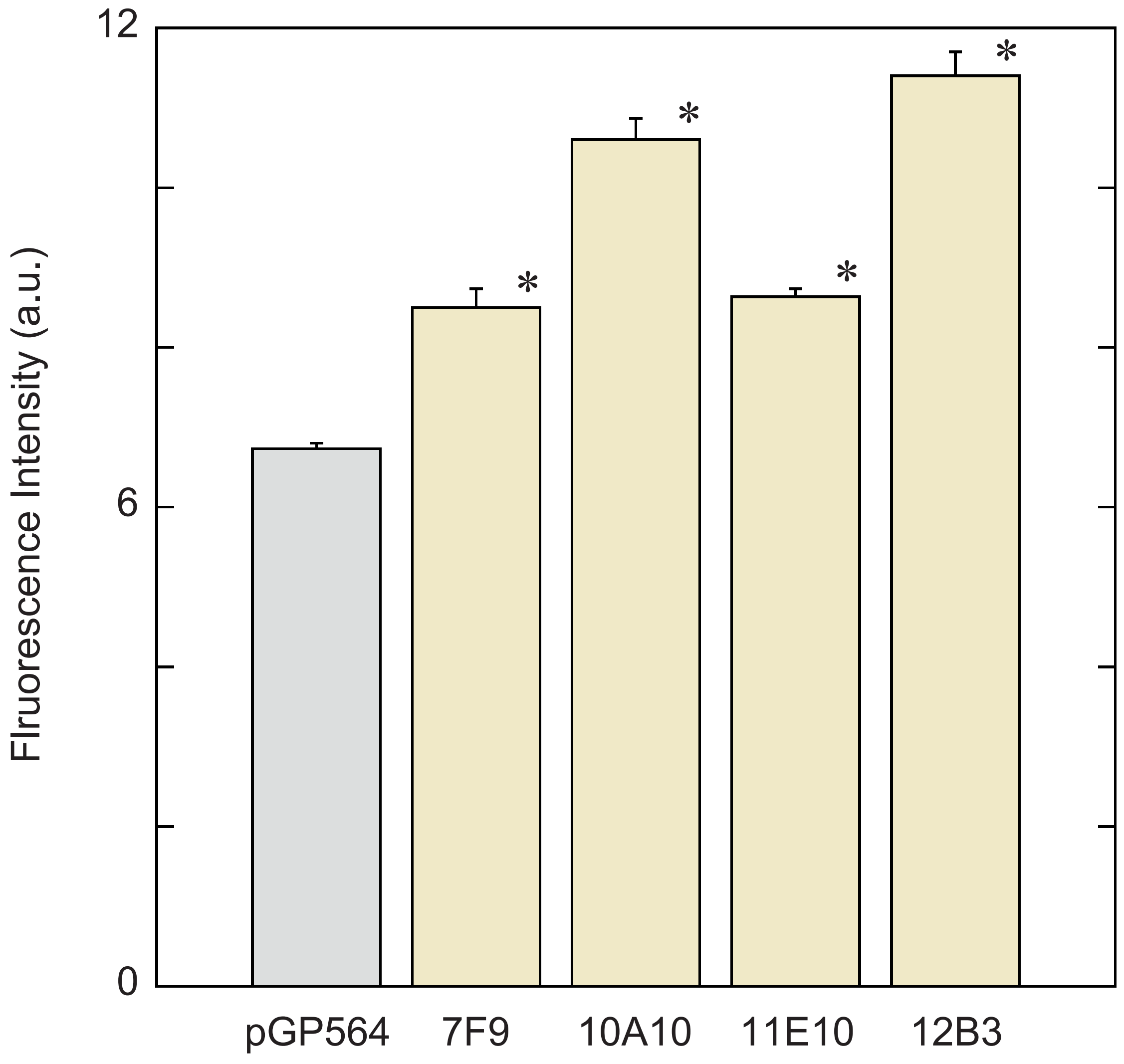

Supplementary Figure S3. Effects of plasmids on the DIT1t strain. DNA of each plasmid vector—pGP564 (Control), 7F9, 10A10, 11E10, or 12B3 (Extended Data Table 1) —was transformed into the DIT1t strain. GFP fluorescence intensities of transformants were measured by flow cytometry. Three or four independent experiments were performed. Error bars are standard deviations. *, $P < 0.01$ by Student' s t-test compared with Control.

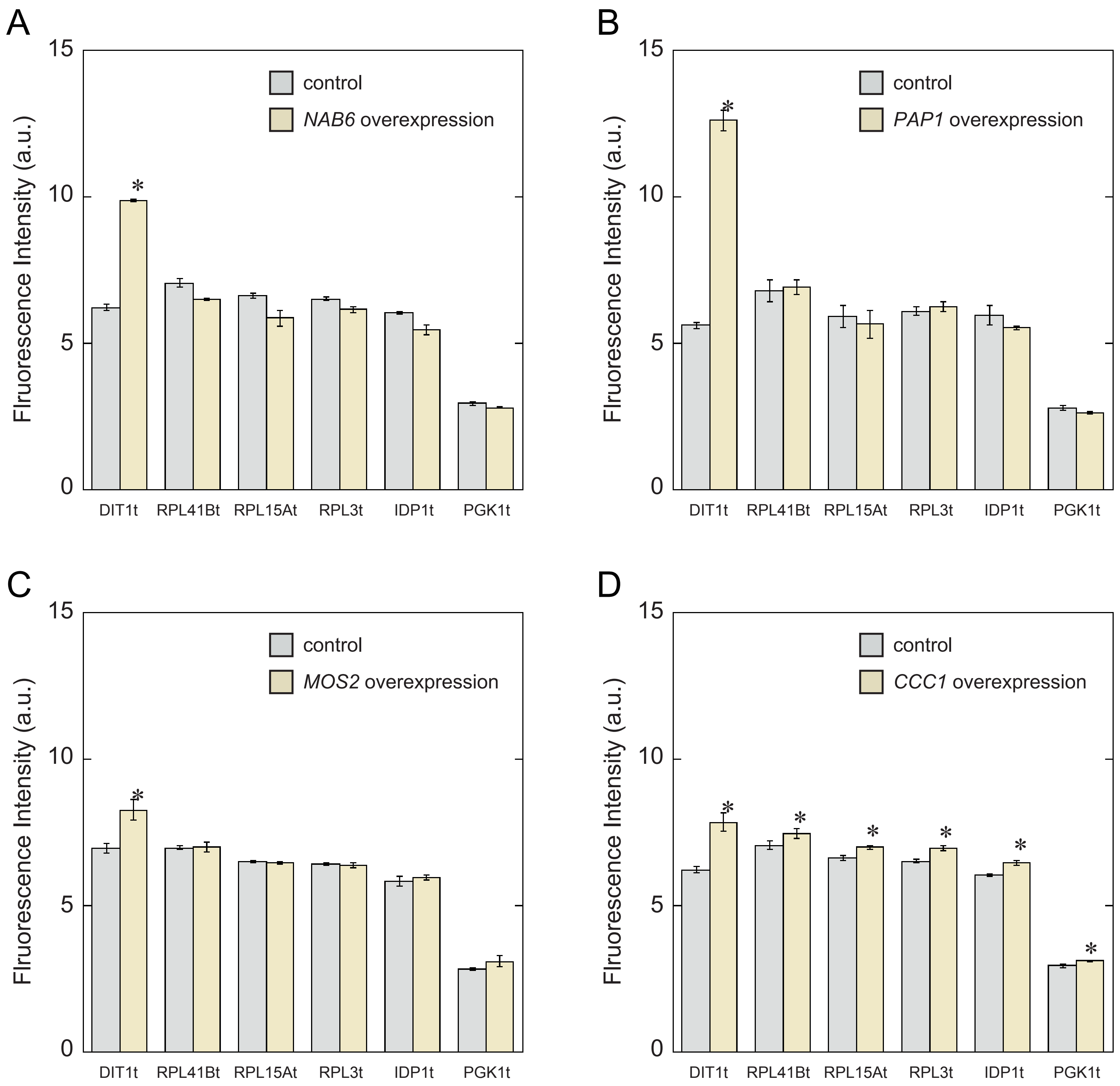

Supplementary Figure S4. Effects of enhancement of the top-five-ranked terminators. (A) Strains DIT1t, RPL41Bt, RPL15At, RPL3t, IDP1t, and PGK1t harboring GFP under the control of the *TDH3* promoter and each terminator were transformed with (A) pGP564 or pGP564-NAB6 vector; (B) pGP564 or pGP564-PAP1 vector; (C) pGP564 or pGP564-MOS2 vector; or (D) pGP564 or pGP564-CCC1 vector. The resulting GFP fluorescence intensities were measured by flow cytometry. Three or four independent experiments were performed. Error bars are standard deviations. *, P < 0.01 by Student' s t-test compared with pGP564.

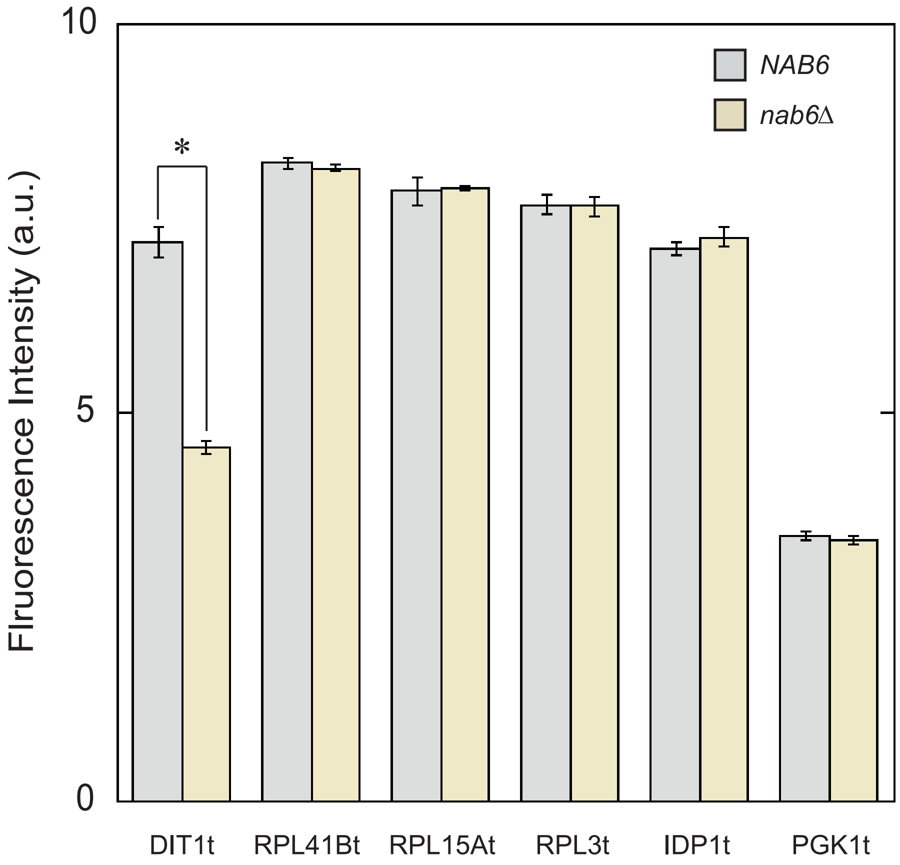

Supplementary Figure S5. Effects of *NAB6* deletion on the activities of the top-five-ranked terminators. Strains DIT1t, RPL41Bt, RPL15At, RPL3t, IDP1t, and PGK1t harboring GFP under the control of the *TDH3* promoter. The resulting GFP fluorescence intensities were measured by flow cytometry. Four independent experiments were performed. Error bars are standard deviations. *, $P < 0.01$ by Student' s t-test compared between *NAB6* and *nab6*Δ background.

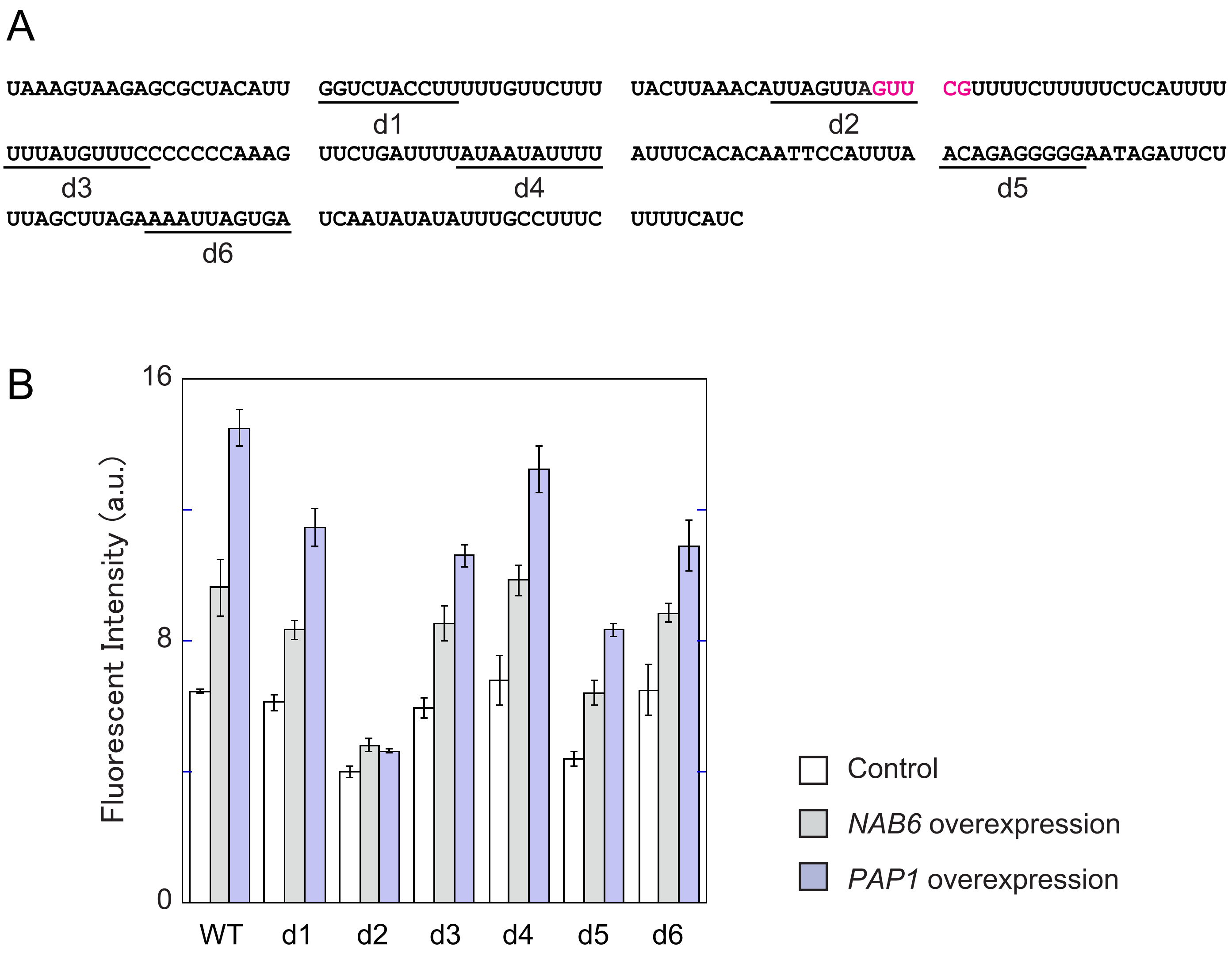

Supplementary Figure S6. Deletion analysis of the *DIT1* terminator for identification of the Pap1p- and Nab6p-recruiting region. (A) Sequence of the DIT1 3′-UTR. Deletion sites (d1 to d6) are underlined. (B) Strains harboring the DIT1 terminator region (Control) with deletion of d1 to d6 were constructed and then transformed with pGP564 (white), pGP564-NAB6 (gray), or pGP564-PAP1 (blue). The GFP fluorescence intensities were measured by flow cytometry. Three independents experiments were performed. Error bars are standard deviations.

	28		29	
PGK1t	**GATCA**			**TTTTTTTTCTT**
DIT1t-inserted *PGK1t*	**GATCA**	**AAAACATTAGTTAGTTCG**		**TTTTTTTTCTT**

	17		29	
PGK1t	**TTGAA**	**TCGATAGATCA**		**TTTTTTTTCTT**
DIT1t-substituted *PGK1t*		**CATTAGTTAGTTCG**		

Supplementary Figure S7. Terminator regions of *DIT1t*-inserted *PGK1t* (A) and *DIT1t*-substituted *PGK1t* (B). The position of insertion or substitution of the sequence of *DIT1t* is indicated above the sequence of *PGK1t*. The candidate cis sequence, AGTTCG, is indicated in red.

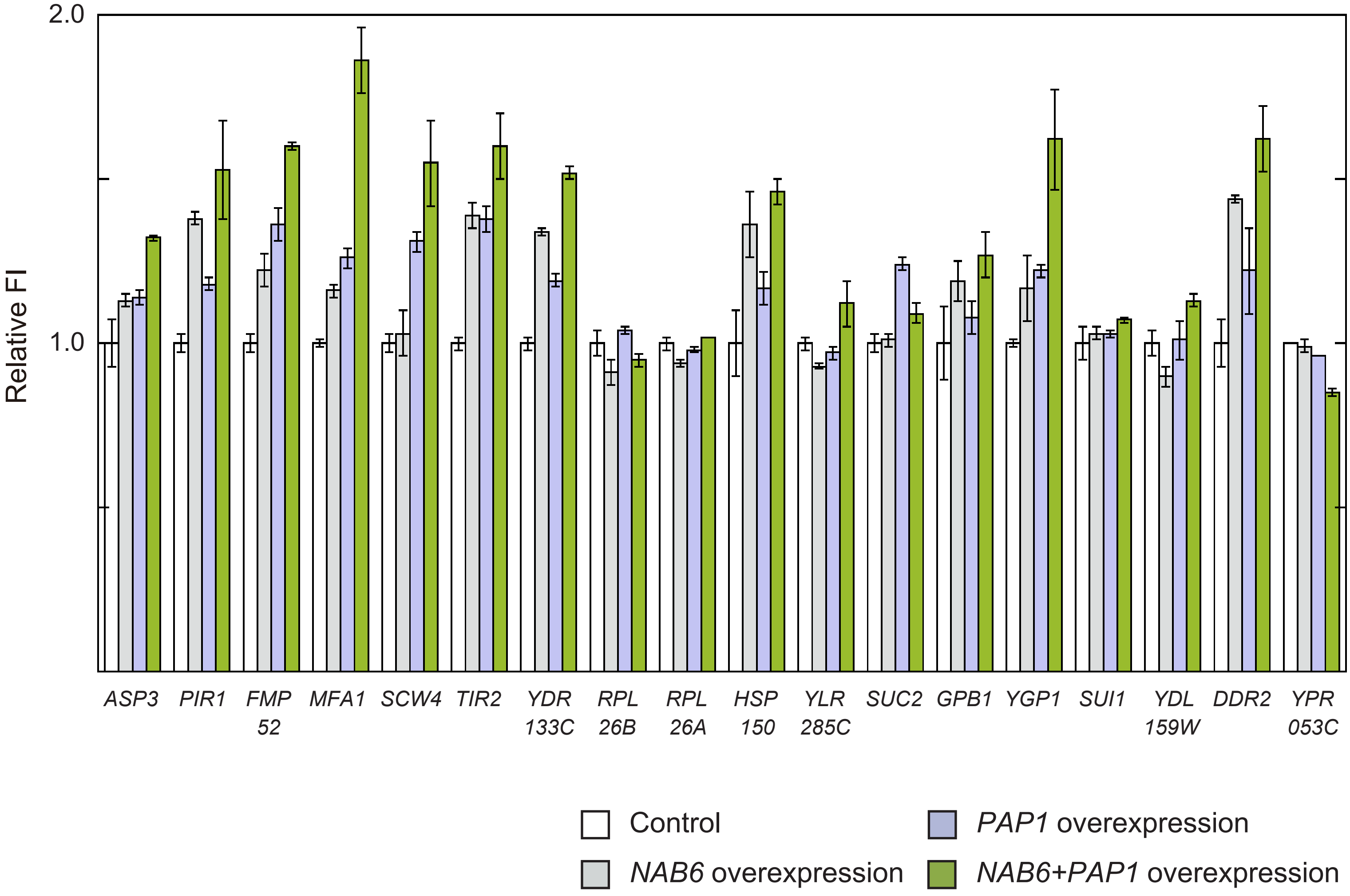

Supplementary Figure S8. Activation of terminators of Nab6p-binding mRNA genes by overexpression of *NAB6* and *PAP1*. Strains harboring each terminator region were constructed and then transformed with pGP564 (white), pGP564-NAB6 (gray), pGP564-PAP1 (blue), or pGP564-NAB6-PAP1 (green). GFP fluorescence intensities were then measured by flow cytometry. Three independent experiments were performed. Error bars are standard deviations.

Supplementary Table S1. Genes included in genomic locus

Plasmid name	Chomosome location	Genes in the genomic locus
7F9	chr. 7 950k–960k	YHB1, MOS2*, SPG1, YGR237C, KEL2, PEX2
10A10, 10B2, 10D6, 10F2	chr. 11 435k–450k	AUR1, MRP17, DID4, MET14, VPS1, PAP1*, ECM9, YKR005C
11E10	chr. 12 573k–583k	MSC3, CCC1*, RSA3, UTP13, YLR222C-A
12B3, 12B4	chr. 13 30k–38k	YML119W, NGL3, NAB6*, YML116W-A

*: Asterisk shows enhanced genes for the *DIT1* terminator

Supplementary Table S2. DNA Sequences of terminator regions.

Name	DNA sequence
d1	TAAAGTAAGAGCGCTACATTTTTGTTCTTTTACTTAAACATTAGTTAGTT<br>CGTTTTCTTTTTCTCATTTTTTTATGTTTCCCCCCCAAAGTTCTGATTTT<br>ATAATATTTTATTTCACACAATTCCATTTAACAGAGGGGGAATAGATTCT<br>TTAGCTTAGAAAATTAGTGATCAATATATATTTGCCTTTCTTTTCATCTT<br>TTCAGTGATATTAATGGTTTCGAGACACTGCAATGGCCCT
d2	TAAAGTAAGAGCGCTACATTGGTCTACCTTTTTGTTCTTTTACTTAAACA<br>CGTTTTCTTTTTCTCATTTTTTTATGTTTCCCCCCCAAAGTTCTGATTTT<br>ATAATATTTTATTTCACACAATTCCATTTAACAGAGGGGGAATAGATTCT<br>TTAGCTTAGAAAATTAGTGATCAATATATATTTGCCTTTCTTTTCATCTT<br>TTCAGTGATATTAATGGTTTCGAGACACTGCAATGGCCCT
d3	TAAAGTAAGAGCGCTACATTGGTCTACCTTTTTGTTCTTTTACTTAAACA<br>TTAGTTAGTTCGTTTTCTTTTTCTCATTTTCCCCCCAAAGTTCTGATTTT<br>ATAATATTTTATTTCACACAATTCCATTTAACAGAGGGGGAATAGATTCT<br>TTAGCTTAGAAAATTAGTGATCAATATATATTTGCCTTTCTTTTCATCTT<br>TTCAGTGATATTAATGGTTTCGAGACACTGCAATGGCCCT
d4	TAAAGTAAGAGCGCTACATTGGTCTACCTTTTTGTTCTTTTACTTAAACA<br>TTAGTTAGTTCGTTTTCTTTTTCTCATTTTTTTATGTTTCCCCCCCAAAG<br>TTCTGATTTTATTTCACACAATTCCATTTAACAGAGGGGGAATAGATTCT<br>TTAGCTTAGAAAATTAGTGATCAATATATATTTGCCTTTCTTTTCATCTT<br>TTCAGTGATATTAATGGTTTCGAGACACTGCAATGGCCCT
d5	TAAAGTAAGAGCGCTACATTGGTCTACCTTTTTGTTCTTTTACTTAAACA<br>TTAGTTAGTTCGTTTTCTTTTTCTCATTTTTTTATGTTTCCCCCCCAAAG<br>TTCTGATTTTATAATATTTTATTTCACACAATTCCATTTAAATAGATTCT<br>TTAGCTTAGAAAATTAGTGATCAATATATATTTGCCTTTCTTTTCATCTT<br>TTCAGTGATATTAATGGTTTCGAGACACTGCAATGGCCCT
d6	TAAAGTAAGAGCGCTACATTGGTCTACCTTTTTGTTCTTTTACTTAAACA<br>TTAGTTAGTTCGTTTTCTTTTTCTCATTTTTTTATGTTTCCCCCCCAAAG<br>TTCTGATTTTATAATATTTTATTTCACACAATTCCATTTAACAGAGGGGG<br>AATAGATTCTTTAGCTTAGATCAATATATATTTGCCTTTCTTTTCATCTT<br>TTCAGTGATATTAATGGTTTCGAGACACTGCAATGGCCCT
d7	TAAAGTAAGAGCGCTACATTGGTCTACCTTTTTCTTTTACTTAAACATTA<br>GTTAGTTCGTTTTCTTTTTCTCATTTTTTTATGTTTCCCCCCCAAAGTTC<br>TGATTTTATAATATTTTATTTCACACAATTCCATTTAACAGAGGGGGAAT<br>AGATTCTTTAGCTTAGAAAATTAGTGATCAATATATATTTGCCTTTCTTT<br>TCATCTTTTCAGTGATATTAATGGTTTCGAGACACTGCAATGGCCCT
d8	TAAAGTAAGAGCGCTACATTGGTCTACCTTTTTGTTTTACTTAAACATTA<br>GTTAGTTCGTTTTCTTTTTCTCATTTTTTTATGTTTCCCCCCCAAAGTTC<br>TGATTTTATAATATTTTATTTCACACAATTCCATTTAACAGAGGGGGAAT<br>AGATTCTTTAGCTTAGAAAATTAGTGATCAATATATATTTGCCTTTCTTT<br>TCATCTTTTCAGTGATATTAATGGTTTCGAGACACTGCAATGGCCCT
d9	TAAAGTAAGAGCGCTACATTGGTCTACCTTTTTGTTCTACTTAAACATTA<br>GTTAGTTCGTTTTCTTTTTCTCATTTTTTTATGTTTCCCCCCCAAAGTTC<br>TGATTTTATAATATTTTATTTCACACAATTCCATTTAACAGAGGGGGAAT<br>AGATTCTTTAGCTTAGAAAATTAGTGATCAATATATATTTGCCTTTCTTT<br>TCATCTTTTCAGTGATATTAATGGTTTCGAGACACTGCAATGGCCCT

d10

TAAAGTAAGAGCGCTACATTGGTCTACCTTTTTGTTCTTTTTAAACATTA
GTTAGTTCGTTTTCTTTTTCTCATTTTTTTATGTTTCCCCCCCAAAGTTC
TGATTTTATAATATTTTATTTCACACAATTCCATTTAACAGAGGGGGAAT
AGATTCTTTAGCTTAGAAAATTAGTGATCAATATATATTTGCCTTTCTTT
TCATCTTTTCAGTGATATTAATGGTTTCGAGACACTGCAATGGCCCT

d11

TAAAGTAAGAGCGCTACATTGGTCTACCTTTTTGTTCTTTTACTACATTA
GTTAGTTCGTTTTCTTTTTCTCATTTTTTTATGTTTCCCCCCCAAAGTTC
TGATTTTATAATATTTTATTTCACACAATTCCATTTAACAGAGGGGGAAT
AGATTCTTTAGCTTAGAAAATTAGTGATCAATATATATTTGCCTTTCTTT
TCATCTTTTCAGTGATATTAATGGTTTCGAGACACTGCAATGGCCCT

d12

TAAAGTAAGAGCGCTACATTGGTCTACCTTTTTGTTCTTTTACTTAATTA
GTTAGTTCGTTTTCTTTTTCTCATTTTTTTATGTTTCCCCCCCAAAGTTC
TGATTTTATAATATTTTATTTCACACAATTCCATTTAACAGAGGGGGAAT
AGATTCTTTAGCTTAGAAAATTAGTGATCAATATATATTTGCCTTTCTTT
TCATCTTTTCAGTGATATTAATGGTTTCGAGACACTGCAATGGCCCT

d13

TAAAGTAAGAGCGCTACATTGGTCTACCTTTTTGTTCTTTTACTTAAACA
GTTAGTTCGTTTTCTTTTTCTCATTTTTTTATGTTTCCCCCCCAAAGTTC
TGATTTTATAATATTTTATTTCACACAATTCCATTTAACAGAGGGGGAAT
AGATTCTTTAGCTTAGAAAATTAGTGATCAATATATATTTGCCTTTCTTT
TCATCTTTTCAGTGATATTAATGGTTTCGAGACACTGCAATGGCCCT

d14

TAAAGTAAGAGCGCTACATTGGTCTACCTTTTTGTTCTTTTACTTAAACA
TTAAGTTCGTTTTCTTTTTCTCATTTTTTTATGTTTCCCCCCCAAAGTTC
TGATTTTATAATATTTTATTTCACACAATTCCATTTAACAGAGGGGGAAT
AGATTCTTTAGCTTAGAAAATTAGTGATCAATATATATTTGCCTTTCTTT
TCATCTTTTCAGTGATATTAATGGTTTCGAGACACTGCAATGGCCCT

d15

TAAAGTAAGAGCGCTACATTGGTCTACCTTTTTGTTCTTTTACTTAAACA
TTAGTTTCGTTTTCTTTTTCTCATTTTTTTATGTTTCCCCCCCAAAGTTC
TGATTTTATAATATTTTATTTCACACAATTCCATTTAACAGAGGGGGAAT
AGATTCTTTAGCTTAGAAAATTAGTGATCAATATATATTTGCCTTTCTTT
TCATCTTTTCAGTGATATTAATGGTTTCGAGACACTGCAATGGCCCT

d16

TAAAGTAAGAGCGCTACATTGGTCTACCTTTTTGTTCTTTTACTTAAACA
TTAGTTAGTTTTTCTTTTTCTCATTTTTTTATGTTTCCCCCCCAAAGTTC
TGATTTTATAATATTTTATTTCACACAATTCCATTTAACAGAGGGGGAAT
AGATTCTTTAGCTTAGAAAATTAGTGATCAATATATATTTGCCTTTCTTT
TCATCTTTTCAGTGATATTAATGGTTTCGAGACACTGCAATGGCCCT

d17

TAAAGTAAGAGCGCTACATTGGTCTACCTTTTTGTTCTTTTACTTAAACA
TTAGTTAGTTCGTCTTTTTCTCATTTTTTTATGTTTCCCCCCCAAAGTTC
TGATTTTATAATATTTTATTTCACACAATTCCATTTAACAGAGGGGGAAT
AGATTCTTTAGCTTAGAAAATTAGTGATCAATATATATTTGCCTTTCTTT
TCATCTTTTCAGTGATATTAATGGTTTCGAGACACTGCAATGGCCCT

d18

TAAAGTAAGAGCGCTACATTGGTCTACCTTTTTGTTCTTTTACTTAAACA
TTAGTTAGTTCGTTTTTTTCTCATTTTTTTATGTTTCCCCCCCAAAGTTC
TGATTTTATAATATTTTATTTCACACAATTCCATTTAACAGAGGGGGAAT
AGATTCTTTAGCTTAGAAAATTAGTGATCAATATATATTTGCCTTTCTTT
TCATCTTTTCAGTGATATTAATGGTTTCGAGACACTGCAATGGCCCT

d19

TAAAGTAAGAGCGCTACATTGGTCTACCTTTTTGTTCTTTTACTTAAACA
TTAGTTAGTTCGTTTTCTTCTCATTTTTTTATGTTTCCCCCCCAAAGTTC
TGATTTTATAATATTTTATTTCACACAATTCCATTTAACAGAGGGGGAAT
AGATTCTTTAGCTTAGAAAATTAGTGATCAATATATATTTGCCTTTCTTT
TCATCTTTTCAGTGATATTAATGGTTTCGAGACACTGCAATGGCCCT

d20

TAAAGTAAGAGCGCTACATTGGTCTACCTTTTTGTTCTTTTACTTAAACA
TTAGTTAGTTCGTTTTCTTTTCATTTTTTTATGTTTCCCCCCCAAAGTTC
TGATTTTATAATATTTTATTTCACACAATTCCATTTAACAGAGGGGGAAT
AGATTCTTTAGCTTAGAAAATTAGTGATCAATATATATTTGCCTTTCTTT
TCATCTTTTCAGTGATATTAATGGTTTCGAGACACTGCAATGGCCCT

d21

TAAAGTAAGAGCGCTACATTGGTCTACCTTTTTGTTCTTTTACTTAAACA
TTAGTTAGTTCGTTTTCTTTTTCTTTTTTTATGTTTCCCCCCCAAAGTTC
TGATTTTATAATATTTTATTTCACACAATTCCATTTAACAGAGGGGGAAT
AGATTCTTTAGCTTAGAAAATTAGTGATCAATATATATTTGCCTTTCTTT
TCATCTTTTCAGTGATATTAATGGTTTCGAGACACTGCAATGGCCCT

Name	DNA sequence	Position of the mutated nucleotide
m1	TAAAGTAAGAGCGCTACATTGGTCTACCTTTTTGTTCTTTTACTTAAACA<br>TTAGTTgGTTCGTTTTCTTTTTCTCATTTTTTTATGTTTCCCCCCCAAAG<br>TTCTGATTTTATAATATTTTATTTCACACAATTCCATTTAACAGAGGGGG<br>AATAGATTCTTTAGCTTAGAAAATTAGTGATCAATATATATTTGCCTTTC<br>TTTTCATCTTTTCAGTGATATTAATGGTTTCGAGACACTGCAATGGCCCT	The 5th A to G in "AGTTAGTTCG"
m2	TAAAGTAAGAGCGCTACATTGGTCTACCTTTTTGTTCTTTTACTTAAACA<br>TTAGTTcGTTCGTTTTCTTTTTCTCATTTTTTTATGTTTCCCCCCCAAAG<br>TTCTGATTTTATAATATTTTATTTCACACAATTCCATTTAACAGAGGGGG<br>AATAGATTCTTTAGCTTAGAAAATTAGTGATCAATATATATTTGCCTTTC<br>TTTTCATCTTTTCAGTGATATTAATGGTTTCGAGACACTGCAATGGCCCT	The 5th A to C in "AGTTAGTTCG"
m3	TAAAGTAAGAGCGCTACATTGGTCTACCTTTTTGTTCTTTTACTTAAACA<br>TTAGTTtGTTCGTTTTCTTTTTCTCATTTTTTTATGTTTCCCCCCCAAAG<br>TTCTGATTTTATAATATTTTATTTCACACAATTCCATTTAACAGAGGGGG<br>AATAGATTCTTTAGCTTAGAAAATTAGTGATCAATATATATTTGCCTTTC<br>TTTTCATCTTTTCAGTGATATTAATGGTTTCGAGACACTGCAATGGCCCT	The 5th A to T in "AGTTAGTTCG"
m4	TAAAGTAAGAGCGCTACATTGGTCTACCTTTTTGTTCTTTTACTTAAACA<br>TTAGTTAaTTCGTTTTCTTTTTCTCATTTTTTTATGTTTCCCCCCCAAAG<br>TTCTGATTTTATAATATTTTATTTCACACAATTCCATTTAACAGAGGGGG<br>AATAGATTCTTTAGCTTAGAAAATTAGTGATCAATATATATTTGCCTTTC<br>TTTTCATCTTTTCAGTGATATTAATGGTTTCGAGACACTGCAATGGCCCT	The 6th G to A in "AGTTAGTTCG"
m5	TAAAGTAAGAGCGCTACATTGGTCTACCTTTTTGTTCTTTTACTTAAACA<br>TTAGTTAcTTCGTTTTCTTTTTCTCATTTTTTTATGTTTCCCCCCCAAAG<br>TTCTGATTTTATAATATTTTATTTCACACAATTCCATTTAACAGAGGGGG<br>AATAGATTCTTTAGCTTAGAAAATTAGTGATCAATATATATTTGCCTTTC<br>TTTTCATCTTTTCAGTGATATTAATGGTTTCGAGACACTGCAATGGCCCT	The 6th G to C in "AGTTAGTTCG"
m6	TAAAGTAAGAGCGCTACATTGGTCTACCTTTTTGTTCTTTTACTTAAACA<br>TTAGTTAtTTCGTTTTCTTTTTCTCATTTTTTTATGTTTCCCCCCCAAAG<br>TTCTGATTTTATAATATTTTATTTCACACAATTCCATTTAACAGAGGGGG<br>AATAGATTCTTTAGCTTAGAAAATTAGTGATCAATATATATTTGCCTTTC<br>TTTTCATCTTTTCAGTGATATTAATGGTTTCGAGACACTGCAATGGCCCT	The 6th G to T in "AGTTAGTTAG"
m7	TAAAGTAAGAGCGCTACATTGGTCTACCTTTTTGTTCTTTTACTTAAACA<br>TTAGTTAGaTCGTTTTCTTTTTCTCATTTTTTTATGTTTCCCCCCCAAAG<br>TTCTGATTTTATAATATTTTATTTCACACAATTCCATTTAACAGAGGGGG<br>AATAGATTCTTTAGCTTAGAAAATTAGTGATCAATATATATTTGCCTTTC<br>TTTTCATCTTTTCAGTGATATTAATGGTTTCGAGACACTGCAATGGCCCT	The 7th T to A in "AGTTAGTTAG"
m8	TAAAGTAAGAGCGCTACATTGGTCTACCTTTTTGTTCTTTTACTTAAACA<br>TTAGTTAGgTCGTTTTCTTTTTCTCATTTTTTTATGTTTCCCCCCCAAAG<br>TTCTGATTTTATAATATTTTATTTCACACAATTCCATTTAACAGAGGGGG<br>AATAGATTCTTTAGCTTAGAAAATTAGTGATCAATATATATTTGCCTTTC<br>TTTTCATCTTTTCAGTGATATTAATGGTTTCGAGACACTGCAATGGCCCT	The 7th T to G in "AGTTAGTTAG"
m9	TAAAGTAAGAGCGCTACATTGGTCTACCTTTTTGTTCTTTTACTTAAACA<br>TTAGTTAGcTCGTTTTCTTTTTCTCATTTTTTTATGTTTCCCCCCCAAAG<br>TTCTGATTTTATAATATTTTATTTCACACAATTCCATTTAACAGAGGGGG<br>AATAGATTCTTTAGCTTAGAAAATTAGTGATCAATATATATTTGCCTTTC<br>TTTTCATCTTTTCAGTGATATTAATGGTTTCGAGACACTGCAATGGCCCT	The 7th T to C in "AGTTAGTTCG"
m10	TAAAGTAAGAGCGCTACATTGGTCTACCTTTTTGTTCTTTTACTTAAACA<br>TTAGTTAGTaCGTTTTCTTTTTCTCATTTTTTTATGTTTCCCCCCCAAAG<br>TTCTGATTTTATAATATTTTATTTCACACAATTCCATTTAACAGAGGGGG<br>AATAGATTCTTTAGCTTAGAAAATTAGTGATCAATATATATTTGCCTTTC<br>TTTTCATCTTTTCAGTGATATTAATGGTTTCGAGACACTGCAATGGCCCT	The 8th T to A in "AGTTAGTTAG"
m11	TAAAGTAAGAGCGCTACATTGGTCTACCTTTTTGTTCTTTTACTTAAACA<br>TTAGTTAGTgCGTTTTCTTTTTCTCATTTTTTTATGTTTCCCCCCCAAAG<br>TTCTGATTTTATAATATTTTATTTCACACAATTCCATTTAACAGAGGGGG<br>AATAGATTCTTTAGCTTAGAAAATTAGTGATCAATATATATTTGCCTTTC<br>TTTTCATCTTTTCAGTGATATTAATGGTTTCGAGACACTGCAATGGCCCT	The 8th T to G in "AGTTAGTTAG"

m12	TAAAGTAAGAGCGCTACATTGGTCTACCTTTTTGTTCTTTTACTTAAACA<br>TTAGTTAGTcCGTTTTCTTTTTCTCATTTTTTTATGTTTCCCCCCCAAAG<br>TTCTGATTTTATAATATTTTATTTCACACAATTCCATTTAACAGAGGGGG<br>AATAGATTCTTTAGCTTAGAAAATTAGTGATCAATATATATATTTGCCTTTC<br>TTTTCATCTTTTCAGTGATATTAATGGTTTCGAGACACTGCAATGGCCCT	The 8th T to C in "AGTTAGTTAG"
m13	TAAAGTAAGAGCGCTACATTGGTCTACCTTTTTGTTCTTTTACTTAAACA<br>TTAGTTAGTTaGTTTTCTTTTTCTCATTTTTTTATGTTTCCCCCCCAAAG<br>TTCTGATTTTATAATATTTTATTTCACACAATTCCATTTAACAGAGGGGG<br>AATAGATTCTTTAGCTTAGAAAATTAGTGATCAATATATATATTTGCCTTTC<br>TTTTCATCTTTTCAGTGATATTAATGGTTTCGAGACACTGCAATGGCCCT	The 9th C to A in "AGTTAGTTCG"
m14	TAAAGTAAGAGCGCTACATTGGTCTACCTTTTTGTTCTTTTACTTAAACA<br>TTAGTTAGTTgGTTTTCTTTTTCTCATTTTTTTATGTTTCCCCCCCAAAG<br>TTCTGATTTTATAATATTTTATTTCACACAATTCCATTTAACAGAGGGGG<br>AATAGATTCTTTAGCTTAGAAAATTAGTGATCAATATATATATTTGCCTTTC<br>TTTTCATCTTTTCAGTGATATTAATGGTTTCGAGACACTGCAATGGCCCT	The 9th C to G in "AGTTAGTTCG"
m15	TAAAGTAAGAGCGCTACATTGGTCTACCTTTTTGTTCTTTTACTTAAACA<br>TTAGTTAGTTtGTTTTCTTTTTCTCATTTTTTTATGTTTCCCCCCCAAAG<br>TTCTGATTTTATAATATTTTATTTCACACAATTCCATTTAACAGAGGGGG<br>AATAGATTCTTTAGCTTAGAAAATTAGTGATCAATATATATATTTGCCTTTC<br>TTTTCATCTTTTCAGTGATATTAATGGTTTCGAGACACTGCAATGGCCCT	The 9th C to T in "AGTTAGTTCG"
m16	TAAAGTAAGAGCGCTACATTGGTCTACCTTTTTGTTCTTTTACTTAAACA<br>TTAGTTAGTTCaTTTTCTTTTTCTCATTTTTTTATGTTTCCCCCCCAAAG<br>TTCTGATTTTATAATATTTTATTTCACACAATTCCATTTAACAGAGGGGG<br>AATAGATTCTTTAGCTTAGAAAATTAGTGATCAATATATATATTTGCCTTTC<br>TTTTCATCTTTTCAGTGATATTAATGGTTTCGAGACACTGCAATGGCCCT	The 10th G to A in "AGTTAGTTCG"
m17	TAAAGTAAGAGCGCTACATTGGTCTACCTTTTTGTTCTTTTACTTAAACA<br>TTAGTTAGTTCcTTTTCTTTTTCTCATTTTTTTATGTTTCCCCCCCAAAG<br>TTCTGATTTTATAATATTTTATTTCACACAATTCCATTTAACAGAGGGGG<br>AATAGATTCTTTAGCTTAGAAAATTAGTGATCAATATATATATTTGCCTTTC<br>TTTTCATCTTTTCAGTGATATTAATGGTTTCGAGACACTGCAATGGCCCT	The 10th G to C in "AGTTAGTTCG"
m18	TAAAGTAAGAGCGCTACATTGGTCTACCTTTTTGTTCTTTTACTTAAACA<br>TTAGTTAGTTCtTTTTCTTTTTCTCATTTTTTTATGTTTCCCCCCCAAAG<br>TTCTGATTTTATAATATTTTATTTCACACAATTCCATTTAACAGAGGGGG<br>AATAGATTCTTTAGCTTAGAAAATTAGTGATCAATATATATATTTGCCTTTC<br>TTTTCATCTTTTCAGTGATATTAATGGTTTCGAGACACTGCAATGGCCCT	The 10th G to T in "AGTTAGTTCG"
m19	TAAAGTAAGAGCGCTACATTGGTCTACCTTTTTGTTCTTTTACTTAAACA<br>TTgGTTAGTTCGTTTTCTTTTTCTCATTTTTTTATGTTTCCCCCCCAAAG<br>TTCTGATTTTATAATATTTTATTTCACACAATTCCATTTAACAGAGGGGG<br>AATAGATTCTTTAGCTTAGAAAATTAGTGATCAATATATATATTTGCCTTTC<br>TTTTCATCTTTTCAGTGATATTAATGGTTTCGAGACACTGCAATGGCCCT	The 1st A to G in "AGTTAGTTCG"
m20	TAAAGTAAGAGCGCTACATTGGTCTACCTTTTTGTTCTTTTACTTAAACA<br>TTcGTTAGTTCGTTTTCTTTTTCTCATTTTTTTATGTTTCCCCCCCAAAG<br>TTCTGATTTTATAATATTTTATTTCACACAATTCCATTTAACAGAGGGGG<br>AATAGATTCTTTAGCTTAGAAAATTAGTGATCAATATATATATTTGCCTTTC<br>TTTTCATCTTTTCAGTGATATTAATGGTTTCGAGACACTGCAATGGCCCT	The 1st A to C in "AGTTAGTTCG"
m21	TAAAGTAAGAGCGCTACATTGGTCTACCTTTTTGTTCTTTTACTTAAACA<br>TTtGTTAGTTCGTTTTCTTTTTCTCATTTTTTTATGTTTCCCCCCCAAAG<br>TTCTGATTTTATAATATTTTATTTCACACAATTCCATTTAACAGAGGGGG<br>AATAGATTCTTTAGCTTAGAAAATTAGTGATCAATATATATATTTGCCTTTC<br>TTTTCATCTTTTCAGTGATATTAATGGTTTCGAGACACTGCAATGGCCCT	The 1st A to T in "AGTTAGTTCG"
m22	TAAAGTAAGAGCGCTACATTGGTCTACCTTTTTGTTCTTTTACTTAAACA<br>TTAaTTAGTTCGTTTTCTTTTTCTCATTTTTTTATGTTTCCCCCCCAAAG<br>TTCTGATTTTATAATATTTTATTTCACACAATTCCATTTAACAGAGGGGG<br>AATAGATTCTTTAGCTTAGAAAATTAGTGATCAATATATATATTTGCCTTTC<br>TTTTCATCTTTTCAGTGATATTAATGGTTTCGAGACACTGCAATGGCCCT	The 2nd G to A in "AGTTAGTTCG"

m23

TAAAGTAAGAGCGCTACATTGGTCTACCTTTTTGTTCTTTTACTTAAACA
TTAcTTAGTTCGTTTTCTTTTTCTCATTTTTTTATGTTTCCCCCCCAAAG
TTCTGATTTTATAATATTTTATTTCACACAATTCCATTTAACAGAGGGGG
AATAGATTCTTTAGCTTAGAAAATTAGTGATCAATATATATTTGCCTTTC
TTTTCATCTTTTCAGTGATATTAATGGTTTCGAGACACTGCAATGGCCCT

The 2nd G to C in "AGTTAGTTCG"

m24

TAAAGTAAGAGCGCTACATTGGTCTACCTTTTTGTTCTTTTACTTAAACA
TTAtTTAGTTCGTTTTCTTTTTCTCATTTTTTTATGTTTCCCCCCCAAAG
TTCTGATTTTATAATATTTTATTTCACACAATTCCATTTAACAGAGGGGG
AATAGATTCTTTAGCTTAGAAAATTAGTGATCAATATATATTTGCCTTTC
TTTTCATCTTTTCAGTGATATTAATGGTTTCGAGACACTGCAATGGCCCT

The 2nd G to T in "AGTTAGTTCG"

m25

TAAAGTAAGAGCGCTACATTGGTCTACCTTTTTGTTCTTTTACTTAAACA
TTAGaTAGTTCGTTTTCTTTTTCTCATTTTTTTATGTTTCCCCCCCAAAG
TTCTGATTTTATAATATTTTATTTCACACAATTCCATTTAACAGAGGGGG
AATAGATTCTTTAGCTTAGAAAATTAGTGATCAATATATATTTGCCTTTC
TTTTCATCTTTTCAGTGATATTAATGGTTTCGAGACACTGCAATGGCCCT

The 3rd T to A in "AGTTAGTTCG"

m26

TAAAGTAAGAGCGCTACATTGGTCTACCTTTTTGTTCTTTTACTTAAACA
TTAGgTAGTTCGTTTTCTTTTTCTCATTTTTTTATGTTTCCCCCCCAAAG
TTCTGATTTTATAATATTTTATTTCACACAATTCCATTTAACAGAGGGGG
AATAGATTCTTTAGCTTAGAAAATTAGTGATCAATATATATTTGCCTTTC
TTTTCATCTTTTCAGTGATATTAATGGTTTCGAGACACTGCAATGGCCCT

The 3rd T to G in "AGTTAGTTCG"

m27

TAAAGTAAGAGCGCTACATTGGTCTACCTTTTTGTTCTTTTACTTAAACA
TTAGcTAGTTCGTTTTCTTTTTCTCATTTTTTTATGTTTCCCCCCCAAAG
TTCTGATTTTATAATATTTTATTTCACACAATTCCATTTAACAGAGGGGG
AATAGATTCTTTAGCTTAGAAAATTAGTGATCAATATATATTTGCCTTTC
TTTTCATCTTTTCAGTGATATTAATGGTTTCGAGACACTGCAATGGCCCT

The 3rd T to C in "AGTTAGTTCG"

m28

TAAAGTAAGAGCGCTACATTGGTCTACCTTTTTGTTCTTTTACTTAAACA
TTAGTaAGTTCGTTTTCTTTTTCTCATTTTTTTATGTTTCCCCCCCAAAG
TTCTGATTTTATAATATTTTATTTCACACAATTCCATTTAACAGAGGGGG
AATAGATTCTTTAGCTTAGAAAATTAGTGATCAATATATATTTGCCTTTC
TTTTCATCTTTTCAGTGATATTAATGGTTTCGAGACACTGCAATGGCCCT

The 4th T to A in "AGTTAGTTCG"

m29

TAAAGTAAGAGCGCTACATTGGTCTACCTTTTTGTTCTTTTACTTAAACA
TTAGTgAGTTCGTTTTCTTTTTCTCATTTTTTTATGTTTCCCCCCCAAAG
TTCTGATTTTATAATATTTTATTTCACACAATTCCATTTAACAGAGGGGG
AATAGATTCTTTAGCTTAGAAAATTAGTGATCAATATATATTTGCCTTTC
TTTTCATCTTTTCAGTGATATTAATGGTTTCGAGACACTGCAATGGCCCT

The 4th T to G in "AGTTAGTTCG"

m30

TAAAGTAAGAGCGCTACATTGGTCTACCTTTTTGTTCTTTTACTTAAACA
TTAGTcAGTTCGTTTTCTTTTTCTCATTTTTTTATGTTTCCCCCCCAAAG
TTCTGATTTTATAATATTTTATTTCACACAATTCCATTTAACAGAGGGGG
AATAGATTCTTTAGCTTAGAAAATTAGTGATCAATATATATTTGCCTTTC
TTTTCATCTTTTCAGTGATATTAATGGTTTCGAGACACTGCAATGGCCCT

The 4th T to C in "AGTTAGTTCG"

Name	DNA sequence	length
DIT1t	TAAAGTAAGAGCGCTACATTGGTCTACCTTTTTGTTCTTTTACTTAAACA<br>TTAGTTAGTTCGTTTTCTTTTTCTCATTTTTTTATGTTTCCCCCCCAAAG<br>TTCTGATTTTATAATATTTTATTTCACACAATTCCATTTAACAGAGGGGG<br>AATAGATTCTTTAGCTTAGAAAATTAGTGATCAATATATATTTGCCTTTC<br>TTTTCATCTTTTCAGTGATATTAATGGTTTCGAGACACTGCAATGGCCCT	250 bp
PGK1t	ATTGAATTGAATTGAAATCGATAGATCAATTTTTTTCTTTTCTCTTTCCC<br>CATCCTTTACGCTAAAATAATAGTTTATTTTATTTTTTGAATATTTTTTA<br>TTTATATACGTATATATAGACTATTATTTATCTTTTAATGATTATTAAGA<br>TTTTTATTAAAAAAAAATTCGCTCCTCTTTTAATGCCTTTATGCAGTTTT<br>TTTTTCCCATTCGATATTTCTATGTTCGGGTTCAGCGTATTTTAAGT	247 bp
DIT1t-inserted PGK1t	ATTGAATTGAATTGAAATCGATAGATCAAAAACATTAGTTAGTTCGTTTT<br>TTTCTTTTCTCTTTCCCCATCCTTTACGCTAAAATAATAGTTTATTTTAT<br>TTTTTGAATATTTTTTATTTATATACGTATATATAGACTATTATTTATCT<br>TTTAATGATTATTAAGATTTTTATTAAAAAAAAATTCGCTCCTCTTTTAA<br>TGCCTTTATGCAGTTTTTTTTTCCCATTCGATATTTCTATGTTCGGGTTC<br>AGCGTATTTTAAGT	264 bp
DIT1t-substituted PGK1t	ATTGAATTGAATTGAAACATTAGTTAGTTCGTTTTTTTCTTTTCTCTTTC<br>CCCATCCTTTACGCTAAAATAATAGTTTATTTTATTTTTTGAATATTTTT<br>TATTTATATACGTATATATAGACTATTATTTATCTTTTAATGATTATTAA<br>GATTTTTATTAAAAAAAAATTCGCTCCTCTTTTAATGCCTTTATGCAGTT<br>TTTTTTTCCCATTCGATATTTCTATGTTCGGGTTCAGCGTATTTTAAGT	249 bp

Supplementary Table S3. List of genes, pimers and amplified terminator regions of *NAB6* -binding mRNA genes.

	Systematic Name	Gene Name	5'-primer	3'-primer	Sequence of amplified region
1	YLR155C	ASP3-1	AGCCTGATCAGTGACTTTTCATC	GCGGCCATATCTACCAGAAA	AGCCTGATCAGTGACTTTTCATCACACTGTGTTTTTATATAGTTCTTAGTAGTAAATATACTCTATAATTTCTACATCCTTTTC<br>ATGATGTGCCAGTTAAGCTATTTTTAGCAAACAAAAAGATGAATTTGAGTATTGAGCACATCATTCACTGTTCAGCTGATACAA<br>AACATATGATTTCTAATATTCACATAGGAAAAATCGCTCCAAACATCGAAGTATCATCGCTACTTTGGTCTTTTATCTGCTTCT<br>CTTCTCTTCGCTCTTTTCACTGCTCGTGCCGCTGCAACGCTCCAACTCAGTTCTATCTCAGCTCCTTCCCCTTATCCCTTGAGC<br>AGTGTCAAGGCGAGGCTGCGTGATGTATTCGTGCGCGAAAAAAAAAACAACTGCAGCACCTGAGTTTCGCGTATGGTCACCCAC<br>TACACTACTCGGTCAGGCTCTTACCAGCTTAACTACAGTTGATCGGACGGGAAACGGTGCTTTCTGGTAGATATGGCCGC
2	YKL164C	PIR1	TCATAAGTTTACCTCTCATAGTCGCA	GGCTCCACGGTACACAAAGT	TCATAAGTTTACCTCTCATAGTCGCATGAAAACATAAAGTCATCGTAGCATATTCTGTACATTAGTCATTTCTGTCTCCAAAGG<br>GCATAATTTGGGTCGGTTCATTTTTTTCTTCTTAATTCGTCTCTTCATTTCATAAAGCTTTCTTTTGAAACCTATGAAATGAGC<br>TTTACAGTTCTTCACTCTTTTTTTTAAAGTTTGTTTATTTTTTTATACTATATACATTATTATTTCTAAAAAATCATTATAAAT<br>TACGTTAAAAGCGCACCTTATACTTTGATAGAGTTTTCCCAGAAAAAAAACCAACGAATTTGATAGTACCAAGACTTCACGGGA<br>TTGAAATCGGATGCAACTTCAACTATTCCTGGGACAGAATATTTCTCAGACATTACTTTGTGTACCGTGGAGCC
3	YER004W	FMP52	ATCAACCAACGGCTTCTTCT	TGTGAGTCCTGGTTTGTTGC	ATCAACCAACGGCTTCTTCTCGCAGCTACAGTTGTATTTTACTGTGACGTACTTTTGCGCGCGTACGGGTTCAATGAATTACGG<br>ATTTTCAACAACTTCACACAATCATAGCTTTTTTTACTTTTTCGCCTCATTATTTTCTTTAAAGAAACGATGATAAACAGAGAT<br>TTATCGTCATGTTATTTCGTTCGTATATATCGTATTTGCGTTTTAGCTGTATAAAGTAAAATACTATATCTAACGTGGCTTTTT<br>TTATTTCCCCGTCTGCCCTCTTATGCTCATAGAAAACACTAATGATCGGTTTGGTATCGTCATAGATGCAGGGTCTTCGGGTTC<br>GAGAATCCATGTGTTTAAGTGGCAGGACACGGAATCGCTTCTTCATGCAACAAACCAGGACTCACA
4	YDR461W	MFA1	GTTATTGCTTAGTTTCTGCGTACA	TAGATCCGCCCTCTTACGAA	GTTATTGCTTAGTTTCTGCGTACAAAAACGTTGTTCTCCCTCCTTTATCTTCCTTTTCCGCTACACCAATATATCATGTTTGTT<br>CGTAATATTTCTTTTTAGACCTAATAATAAATATCCTAAGTAACTATATTATATAAAATATTTTGATACCCTGTACCTGCTTTT<br>GTTATCGTTGTACATCCATGCACACGCTCATATATATATATATATATATATATATGTATATGTACATATACCGCTTACCACCTA<br>CCGTGAAGTATATGTAAGGGTCTTCGCACCCGGATATCCCTTGTGGGATCTTGGATGCGGATGGTGAGTGTAAACAGGCCTCAT<br>AAAGCTGTCTCTGTGTGCGGAAGATGTCGTTTCATCGGCTTCGACCTCCTCCTTATCTTGCTTCATTGTTTCTTCGTAAGAGGG<br>CGGATCTA
5	YGR279C	SCW4	GTCTACTCAATTGTAAAAAAAAACAAA	CCAAGAGGATCGAGAAACCA	GTCTACTCAATTGTAAAAAAAAACAAAAAAAAATCTTGATTTAAAAAACGATACCCAATATTTTTAACTTTAAATTAAGGCTTTG<br>ATAATCTTCTTTTTCTCTGTTATATCAAAGGACTGCTTTCTGGTTTGCCTTCGCTAATCTTTTTTTTTTTTTTTCATTTTCCTA<br>TTTTAGTTCGTTTATGATCTCATTTGTATTTCTGTATATTTGTCTTAATACGAAATACGAGTTTTTATTTTATATTCTACCTTT<br>CTTTTTTTTTTGGCTAAAAGCGTGTACGGTTCTATGTAAATATGTACGATTACATGTGTAATGTTTGTTTGGCCGGGCTCTTCA<br>TCTATGCCGTTGCCTTCTTGCAGGCGGAGTCCTGGATCTGTTACGATGGTTTCTCGATCCTCTTGG
6	YOR010C	TIR2	TTTTAAATGGAATTTTTAATGTTCG	GCCGCCATGCTACTATGATT	TTTTAAATGGAATTTTTAATGTTCGTATATACCAATTTATCTATTGAATAGTTTTTTTAAATTTTACTGTTTCTTTTAAACCT<br>CGAATTATTGTGAGAACTGATTTCATCAACTGGAGGCTGTAGTGTACGTTTTGAAAACGGAAATGATTTACCGTTTTCTTTTAT<br>GATGATGCTAGGATAGGCACCTTCAAACATGCATGTCTTCCTGCCCACATTCTGAAAATATTTAACGATGGATGAAAGAAATCA<br>ACAATAGAAAAGGCCAGTTGTCAGTTTTTATATAATACAGAGAAGTACATATGACAGTTTAAAAGTTAAGCATTAACTGGTAAG<br>TTGAAATAATAATATAATCATAAGCGGAACGAACATTTTCGACACGTACTAAAATCATAGTAGCATGGCGGC
7	YDR133C	YDR133C	TTTCTTATTCTAATTCTAGG	CGAGAGGAGAATCCATTAG	TTTCTTATTCTAATTCTAGGAGGCAACACTCCATAACTAAATGTTTATTATAATAATATTAATATAAAGTTAATATCTCTTAGG<br>TTACACGCAACTAAACTTTTTTTATATATACTGGTTGCATTGTATTTTTATCATATATCTTAATATAGGTTGTTTAAGGTTCGT<br>TAATTCAAAAATAATGTTTTTTTGTTTGCCCAGTCGGTTTACGCTAATCAAGAACATGGATTCAATATCTTACATAATAAGCGG<br>ACGATGAGTCTATCTCGGTTTCCTCTTTTCTGAAATATAATAGTTTTATATTAGTGATTCATTATTACGATCTGGCCGGCTTAT<br>CATTGCTTCTTTTACAAACGTATCCTTTTAAGATTTTATCCGGACCACTGAGCTGGCATTTTTCTTAGCTTGCTTTTGTCTTTG<br>TTGCACGGGCGACTGAACTTCTTGGAAAATCATGTAAGCCGCAATTGCTTACTAAGTCAAAATTCTAATGGATTCTCCTCTCG
8	YGR034W	RPL26B	TTGGAATAAACTAGCTCGACTTTAAC	TGTGGCCAATACTGCTTCAG	TTGGAATAAACTAGCTCGACTTTAACATAGATCATACAAAAAATACAATAAATAAATCCTGTGTTTCTTTTATGTATATTTTTT<br>ATCAATTTCAATTTAGTTTAGTTTAGTTTCTTTAAAAATAAAATTGTAATTTCTCACATTCTGCCTTCGGGTCTCAAGGGCTGG<br>TTGGTAGACGAATGCGCTTGTTTCTAGCTGAGCGGCAGATGCATGAATTCAATGCAAGAAAATGAGAAAACTTCCAATATTAAA<br>ATGTGATTGTTAATTATTAGTATATTTATGACAATATATATATGTACATAGGGTGATTATGAAGATGAGTAAGATGCGAATGGA<br>TATGAGGATATGAATCTTTTTAGAGACAATGAAGATGCAAAGAAGTAGTTAAGCACATTGACCTCAGGGTTGCTATATGCTACT<br>TTTAGTCCGTTGGATTTCTGAAGCAGTATTGGCCACA
9	YLR344W	RPL26A	ATTATGTAATTCCCTGAAAACTTCC	TTTACCCACCTTTTGACGGT	ATTATGTAATTCCCTGAAAACTTCCTCGAAATTTATTGCACTTTTTTTACTCAAACCATTTAATAATCATATGTAAAACGTATA<br>AAATAGTATCCATTCCACTGTACTCAGTGTCTTACGTAAAGGGCTGTATACTGGCACTACCTTTTACTATGGATATGTGCTAGC<br>TTTTTCGGAGAAGAAATGTTCGCCTTACGCTAGAAACCGGGGTTAAGGGGGCGTGAGAATGAATATTGGCAGACAAGAACCGAG<br>ATAGAATCTTAACACAAAAACAGTTCATCCTACTTCTACGCCTTTATACAGTTATACTGAAAGATCTTTGCATAGGGGCATAGT<br>TTAAAATAAAATATAGGGTTGTCATAGCAAACATGCCAAATGTGCTTTCTGATGATGAAGAGCTCCTTAATGGACTAGGAAGTG<br>AGATTATGAAACCCTCTAGACAAGGTAACCATATGGCAAGGACCGTCAAAAGGTGGGTAAA
10	YJL159W	HSP150	TAGTTCTTTTATCCTGATGACTTTTTC	AATAGACGTAGCCACATGCAG	TAGTTCTTTTATCCTGATGACTTTTTCTCATTTGCATTGATTAGAAAGGAAAAAAAGAAGTGTCCTTTTCTACTACTACTCTAG<br>TCGCATCCATTCCTTTGCATTTATCTTTTCTGCGGTTGGCCAATCCATTCTTCCGAGAATTTGGCTAGCCATACTTGATGTTTT<br>CCCATTATTGGTTCGTTTGGCAATGCTAATTTTCTTAATTGCCCCTTATATACTCTTCCATAAAATGTTTTTTTTATAACTAAT<br>TTTCTGTATATCATTATCTAATAATCTTATAAAATGTTAAAAAGACTTGGAAAGCAACGAGTGATCGTGACCACATAATTGCCT<br>CGCTACACGGCAAAAATAAGCCAGTCCTAATGTGTATATTAAAGGCTGCATGTGGCTACGTCTATT
11	YLR285C-A	YLR285C-A	TGAGAATGCCAATTCCGC	TGGTTGTTTCAACTGATTATCCA	TGAGAATGCCAATTCCGCCCACTTACTACTTTTCATGTGTTAAATAGTACGCATAAACACGAGATCTGACTTCGATTGTTTCCT<br>CGTCTTCGTCAAACATCGGTTTCCAATTAACCATTTCAATTAGCTGAGGAACCAAATGGAACTCGTTCTTAGCCAATTCGAAGA<br>ATTCTAAATCCTTCTCCAACAATTTTGGTCTATGCGGTGAAAATACTACCAACGCCTGACCTTTCTCAGCTAATAAATCCTTTG<br>TTGTTTGAAGTAATTTGTGATGTTCCGTATGATTGAAGACTAAATCACTTAAAATGATTAAGTCAAACTTTCCATTATTGTTAC<br>CTATTTTTTCGATATGTGCCAACAATGGAGAATAATCGTTTCCCCAAATATAACCCTCCGTACTGACATTATTAAAATCTTCAG<br>GAACGTTAGACTTTATATTATAATCGATGTTCTGCATTAAATCAGGATCTGGATAATCAGTTGAAACAACCA
12	YIL162W	SUC2	AGGTTATAAAACTTATTGTCTTTTTT	GCTTGGTATCCATTTCCCTC	AGGTTATAAAACTTATTGTCTTTTTTATTTTTTTCAAAAGCCATTCTAAAGGGCTTTAGCTAACGAGTGACGAATGTAAAACTT<br>TATGATTTCAAAGAATACCTCCAAACCATTGAAAATGTATTTTTATTTTTATTTTCTCCCGACCCCAGTTACCTGGAATTTGTT<br>CTTTATGTACTTTATATAAGTATAATTCTCTTAAAAATTTTTACTACTTTGCAATAGACATCATTTTTTCACGTAATAAACCCA<br>CAATCGTAATGTAGTTGCCTTACACTACTAGGATGGACCTTTTTGCCTTTATCTGTTTTGTTACTGACACAATGAAACCGGGTA<br>AAGTATTAGTTATGTGAAAATTTAAAAGCATTAAGTAGAAGTATACCATATTGTAAAAAAAAAAAGCGTTGTCTTCTACGTAAA<br>AGTGTTCTCAAAAAGAAGTAGTGAGGGAAATGGATACCAAGC
13	YOR371C	GPB1	TAATTAATTCCGCGGGGC	TGAAATGTTGCTGTTAAACCCTT	TAATTAATTCCGCGGGGCGGGGTTCGGATAATCCTTATGTGACAAGAGTGACTAAAGGAAAACGAGAAAATTTTTTCGTGGAAC<br>TTGAAAAAAAAAAAAAAAAAAAAAAAAGAAAACAGAACAAGTGAAGAAGATAAAAAAAGGTTCTATATCGCACTATACAACAATA<br>GTAGCGTTTCCCGTGTTCTTTTCCCGTCATTAAATATATTCAAAAGTTGTTGCACATTTGATTTTACAGAAGTAATATACACTG<br>ATTGTGCGTACGGTTAATTCGCAACTAAATTCAATCAGTACAAGAAAAATTTTTATTCCGTTCCATCATTATTGAAAGATAAAC<br>TATCACATTGCGATAATAAACTAAAAGACACTAGGATACTGCCTTCGGATATAAACATTTAAGTTTAATTTATTACTGTTTTTT<br>TAAGGGTTTAACAGCAACATTTCA
14	YNL160W	YGP1	AGATATTAAATCTAGCGAAGCATAGAG	GACACAAAAGAAGGGGGTGA	AGATATTAAATCTAGCGAAGCATAGAGATTCTTTTTTTACCAGTCATCATAACCATTATTTTACAAAATTTCCACGCAACAGCT<br>TTTATTTTCGTTCGTAATAGGAACCATACTTCCCATTAAAGCAGTATGATTGTTTTATACACCTTTTTATTGTTATTTAAGACA<br>TACCCCAAATAATTAATTAATTAATTGATGAAAGAATCATATAAATTCTTGGCCATACATATTTTTTTATTATCTTTTGCTACA<br>TTGACCATAGGTGAATATCTTCCCGGGGTGACACTCCACTGAAGTGGGAAAAAAAGAAAAGTTTTAAAACATTGCTCACCTTAA<br>GTCTCGAAGGTCTAGCATTCACTACCTGTAAGTGTCAAGACCCCACATGTATACATGGATACAATAAAGAAACAATGTCCGTAT<br>AATTTGTATACATTTTACTACAGATAAGGGATCTCACCCCCTTCTTTTGTGTC
15	YNL244C	SUI1	GTTCAAGGCTTACGCCGAGT	CATTCTTGAGAGTCTCCGGC	GTTCAAGGCTTACGCCGAGTCGCTTACTCGCTGCTCGCTTTCTTTCTTAGTGTTGTTATCCTATATAAAACGTAACTGTTTAGT<br>TATTTAAGTCCAAGTGCATATTTACACTATAATATATAATATAACATCTCATCTTTTTTTATTTCTTTTTCTTCTTCTCCCGCC<br>GCGGGCTTACATACAGAGATATAAGAACAAGTGGTACGTAGCATGCAGCTTCTCAAAAGAGATGAGCGTTCAGTTTTGAGTACA<br>GGGAAGAAAGGCCAAATCGAGAGTACATTATCATGGGGTCGGGCGATTTGAATCTGTTGAAATCATGGAATCCCAAGCTTATGA<br>AAAATAGAAAAAAAGTATGGGAGACCGAACAGGATCTAATTACCGAACAGCAGAAGCTTAACACGCGATTGAAAGAAATTGAAA<br>AAGAACGAGAACTAAATGAACTGCTGAACGAATCGAGCAAGGATAAGCCGGAGACTCTCAAGAATG
16	YDL159W-A	YDL159W-A	TCACTGTCCTCGTTATAAGTGATTC	AAAACAGGTTCATTTTGCGG	TCACTGTCCTCGTTATAAGTGATTCGTGGGGTAACGGCGATATCATACTATTATTGGCCCATCTGATGGATTAGAAGACCTACA<br>GGTTAATGTGAAGCGACACTAATGATAAGAAAACTGCATCGGATCATATCTGTTTTTGCAGCGTGGTATATTGGTTGTTGGTCA<br>TGTTTCAACGAAAGACTTTACAGAGAAGGAACTTGAAAGGGCTCAATCTTAACCTGCACCCAGATGTGGGCAATAATGGCCAAT<br>TGCAGGAAAAGACAGAGACTCACCAGGGACAATCTCGAATAGAAGGCCACGTGATGTCTAACATTAATGCAATACAGAATAATA<br>GCAACCTGTTTTTGCGAAGAGGCATAAAAAAAAAACTGACGTTGGATGCGTTTGGTGATGACCAAGCTATATCGAAACCAAACA<br>CTGTGGTAATACAGCAACCGCAAAATGAACCTGTTTT
17	YOL052C-A	DDR2	GAAAAACGCCGCTTACTGC	CTTTGGTTTCATGGCGAAAT	GAAAAACGCCGCTTACTGCCACGATGATGATACCCTATTGACGTTTCTGAAATGTATAATTTCTTTTCTCATCTTCCCCTTTGA<br>TATTCCATCTATAGGCCCCAGAGTAGTAAATTTGGTGCTTTAATTTTTTTTCTCTTCTTCCAGTTCGTCTCTATTTTTTCATTC<br>CATTATATTTATTTATCAGTTTTACTTTCTCAAATATTCTTATATAACACTATTTCATCTACGTAACCGAAAATAAGTAGTTTA<br>ATAACGATAATAATCATAATTGTGATAAAAATATTTACATTTTTCTTACTTGTCGCCCGCTTCGTTTTTGTATAGAACGTACTG<br>TATTAGTTGTCCGTTCTTTTCACGTCTGAAGAGGTCGATTTCTTGCAATTTAACATCAACGTGGTATTTTCCATTATATACAAT<br>CAAAGAATCATCGTCCAGTTCGATTTCGCCATGAAACCAAAG

18	YPR053C	YPR053C	CGCCAGAGGAAAAGCAGCC	AACATTGCGATGAGATCATG	CGCCAGAGGAAAAGCAGCCTTACGAGGCCAAGGCCCAGGCCGATAAGAAGAGATATGAATCCGAAAAGGAGTTATATAACGCCA<br>CTTTGGCTTAGGGATACTTCCTCCCCGCGTTTTTGTCTGTGTGATTTAATTTCTTTTCCTCTTGTTCTTTTTGAAACTTGCTTT<br>ATTATCTCTCTCCTTTCTATTCCTCTTTTCTCTACTGCCCTTTTTCTATATCTATACATATGATTAAATAAATAAATAAAAAAA<br>TTGTATTATTAATATATATTTTATCATCATCGAGTGTTTTTCCTTTTATGTGGCCAGCCGGCCATGCATTAGGAACGTAGACCG<br>AGTGCAGCTCTTTTCAGTCAACGGCAATATCAGTTTAAAGGCGTCCCCTTCATTCAAGTAGTAGCGGAACATCCGTTTCATTCT<br>GATGAAACCCATTCCTTCATATAAGTTTAGAGCCGCCGAATTTTCCACCTCTGTTTCTAACATGATCTCATCGCAATGTT

Supplementary Table S4. Raw data sets of Figures 1 & 2.

Population	Genetic Background	Overexpression	Average log10 value (a.u.) of fluorecent intensity (FI) from 3000 cells	full width at half maximum	Average value of FI	Mean value of Average FI	Standard Deviatoin of Average FI
1	NAB6	–	0.842965	0.125835	6.965703749	6.925901465	0.114721995
	NAB6	–	0.833358	0.125227	6.813307656		
	NAB6	–	0.837103	0.135244	6.872314088		
	NAB6	–	0.837069	0.127771	6.87177609		
	NAB6	–	0.85165	0.12956	7.106405742		
2	NAB6	NAB6	1.01983	0.136569	10.46718741	10.3178616	0.163472469
	NAB6	NAB6	1.00889	0.142878	10.20680928		
	NAB6	NAB6	1.00455	0.14102	10.10531836		
	NAB6	NAB6	1.02045	0.136174	10.48214107		
	NAB6	NAB6	1.01401	0.13718	10.32785186		
3	NAB6	PAP1	1.15279	0.228742	14.21641196	14.6387081	0.648318794
	NAB6	PAP1	1.18566	0.236302	15.33416034		
	NAB6	PAP1	1.13801	0.231115	13.74073614		
	NAB6	PAP1	1.17193	0.232109	14.85696157		
	NAB6	PAP1	1.1774	0.234559	15.04527049		
4	NAB6	NAB6+PAP1	1.28918	0.199016	19.46166533	18.26973542	1.722512728
	NAB6	NAB6+PAP1	1.279	0.197937	19.0107828		
	NAB6	NAB6+PAP1	1.27661	0.206423	18.90645041		
	NAB6	NAB6+PAP1	1.18257	0.126377	15.22544518		
	NAB6	NAB6+PAP1	1.27287	0.201509	18.74433339		
5	nab6	–	0.631576	0.117721	4.281303343	4.219615019	0.036366425
	nab6	–	0.625274	0.119521	4.219626392		
	nab6	–	0.623585	0.124065	4.203247855		
	nab6	–	0.621926	0.118068	4.187222124		
	nab6	–	0.623939	0.120924	4.206675382		
6	nab6	PAP1	0.671667	0.126049	4.695339503	4.704439966	0.031042685
	nab6	PAP1	0.677361	0.128507	4.757305046		
	nab6	PAP1	0.669769	0.132461	4.674864204		
	nab6	PAP1	0.671937	0.123977	4.698259494		
	nab6	PAP1	0.671768	0.130794	4.696431583		

t-Test: Paired Two Sample for Means (p<0.01)

Population	1	2	significant
Mean	6.925901465	10.3178616	
Variance	0.013161136	0.026723248	
Observations	5	5	
Pearson Correlation	0.303251039		
Hypothesized Mean Differer	0		
df	4		
t Stat	-44.91964098		
P(T<=t) one-tail	7.34417E-07		
t Critical one-tail	2.131846786		
P(T<=t) two-tail	1.46883E-06		
t Critical two-tail	2.776445105		

Population	1	3	significant
Mean	6.925901465	14.6387081	
Variance	0.013161136	0.420317259	
Observations	5	5	
Pearson Correlation	0.049011476		
Hypothesized Mean Differer	0		
df	4		
t Stat	-26.41781775		
P(T<=t) one-tail	6.10093E-06		
t Critical one-tail	3.746947388		
P(T<=t) two-tail	1.22019E-05		
t Critical two-tail	4.604094871		

Population	1	4	significant
Mean	6.925901465	18.26973542	
Variance	0.013161136	2.967050097	
Observations	5	5	
Pearson Correlation	0.22813183		
Hypothesized Mean Differe	0		
df	4		
t Stat	-14.92080538		
P(T<=t) one-tail	5.87568E-05		
t Critical one-tail	3.746947388		
P(T<=t) two-tail	0.000117514		
t Critical two-tail	4.604094871		

Population	1	5	significant
Mean	6.925901465	4.219615019	
Variance	0.013161136	0.001322517	
Observations	5	5	
Pearson Correlation	0.164712844		
Hypothesized Mean Differe	0		
df	4		
t Stat	52.85296446		
P(T<=t) one-tail	3.83538E-07		
t Critical one-tail	3.746947388		
P(T<=t) two-tail	7.67075E-07		
t Critical two-tail	4.604094871		

	1	6	significant
Mean	6.925901465	4.704439966	
Variance	0.013161136	0.000963648	
Observations	5	5	
Pearson Correlation	-0.410009984		
Hypothesized Mean Differe	0		
df	4		
t Stat	38.04731044		
P(T<=t) one-tail	1.42504E-06		
t Critical one-tail	3.746947388		
P(T<=t) two-tail	2.85009E-06		
t Critical two-tail	4.604094871		

Population	Genetic Background	Overexpression	Ct value of GFP primer set (Sample)		Ct value of TUB primer set (Standard)	
			repetition 1	repetition 2	repetition 1	repetition 2
1	NAB6	–	14.29	14.3	19.02	18.73
	NAB6	–	13.99	14.02	17.06	17.03
	NAB6	–	14.15	14.16	18.28	18.35
2	NAB6	NAB6	14.06	14.05	18.8	18.64
	NAB6	NAB6	13.87	13.86	17.46	17.39
	NAB6	NAB6	14.08	14.09	18.53	18.64
3	NAB6	PAP1	13.99	13.96	18.5	18.37
	NAB6	PAP1	13.75	13.74	17.2	17.19
	NAB6	PAP1	13.98	14	17.97	18.02
4	NAB6	NAB6+PAP1	13.72	13.7	18.27	18.16
	NAB6	NAB6+PAP1	13.69	13.72	18.1	18.04
	NAB6	NAB6+PAP1	13.67	13.69	17.83	17.9
5	nab6	–	14.61	14.64	19.02	18.88
	nab6	–	14.4	14.43	18.1	18.11
	nab6	–	14.13	14.12	17.24	17.3
6	nab6	PAP1	14.28	14.26	18.13	18.02
	nab6	PAP1	14.33	14.31	18.07	18.12
	nab6	PAP1	13.72	13.71	17.92	18.07

Relative GFP mRNA level to TUB1 = exp(−CtTUB ln2)/exp(−CtGFP ln2)

Population	Genetic Background	Overexpression	Mean of relative mRNA level	SD of relative mRNA level
1	NAB6	–	16.6	0.8
2	NAB6	NAB6	19.9	0.6
3	NAB6	PAP1	16.3	0.4
4	NAB6	NAB6+PAP1	20.5	0.4
5	nab6	–	16.3	0.5
6	nab6	PAP1	15.7	0.4

t-test (P<0.01)

Population 1 vs 2		significant
Pooled variance	0.416666667	
Test statistic	8.854829191	
P value (two-sided)	4.7884E-06	

Population 1 vs 3		no difference
Pooled variance	0.333333333	
Test statistic	0.9	
P value (two-sided)	0.389279262	

Population 1 vs 4		significant
Pooled variance	0.333333333	
Test statistic	11.7	
P value (two-sided)	3.70543E-07	

Population 1 vs 5		no difference
Pooled variance	0.370833333	
Test statistic	0.853281834	
P value (two-sided)	0.413474502	

Population 1 vs 6		no difference
Pooled variance	0.333333333	
Test statistic	2.7	
P value (two-sided)	0.022313365	

Population	Genetic Background	Overexpression	Average log10 value (a.u.) of fluorecent intensity (FI) from 3000 cells	Average value of FI	Mean value of Average FI	Standard Deviatoin of Average FI	Relative FI (DIT1t-- Mean value as a standard)	Mean value of relative FI	SD of relative FI
1	DIT1t	–	0.208579	1.61651225	1.593645778	0.035345049	1.014348528	1.00	0.02
	DIT1t	–	0.211901	1.628924667			1.022137221		
	DIT1t	–	0.191041	1.552533572			0.974202418		
	DIT1t	–	0.197725	1.576612625			0.989311832		
2	DIT1t	NAB6	0.382478	2.412559317	2.485083602	0.056933824	1.513861706	1.56	0.04
	DIT1t	NAB6	0.406602	2.550363005			1.60033242		
	DIT1t	NAB6	0.394329	2.479299541			1.555740664		
	DIT1t	NAB6	0.397612	2.498112543			1.567545672		
3	DIT1t	PAP1	0.621917	4.187135352	3.989157483	0.144353204	2.627393997	2.50	0.09
	DIT1t	PAP1	0.598734	3.96948349			2.490819192		
	DIT1t	PAP1	0.58441	3.840696592			2.410006442		
	DIT1t	PAP1	0.59762	3.959314496			2.484438229		
4	DIT1t-d2	–	-0.0620789	0.866804386	0.877200128	0.00729627	0.54391283	0.55	0.00
	DIT1t-d2	–	-0.0558065	0.879414253			0.551825421		
	DIT1t-d2	–	-0.0561516	0.878715728			0.551387103		
	DIT1t-d2	–	-0.0536135	0.883866146			0.554618949		
5	DIT1t-d2	NAB6	0.0275559	1.065506001	1.077305757	0.016675694	0.668596507	0.68	0.01
	DIT1t-d2	NAB6	0.0418151	1.101070431			0.690912903		
	DIT1t-d2	NAB6	0.0277195	1.065907456			0.668848417		
	DIT1t-d2	NAB6	0.0321105	1.07673914			0.675645212		
6	DIT1t-d2	PAP1	0.0607755	1.150205659	1.118111961	0.024033048	0.721744866	0.70	0.02
	DIT1t-d2	PAP1	0.0391829	1.094417175			0.686738038		
	DIT1t-d2	PAP1	0.0440028	1.106630919			0.694402065		
	DIT1t-d2	PAP1	0.0496808	1.12119409			0.703540338		
7	DIT1t-d7	–	0.246878	1.765541783	1.768654536	0.020868424	1.10786337	1.11	0.01
	DIT1t-d7	–	0.250582	1.780664088			1.117352496		
	DIT1t-d7	–	0.252318	1.787796159			1.121827814		
	DIT1t-d7	–	0.240703	1.740616115			1.092222713		
8	DIT1t-d7	NAB6	0.413258	2.589750944	2.549153998	0.06924128	1.625048037	1.60	0.04
	DIT1t-d7	NAB6	0.388463	2.446036875			1.534868607		
	DIT1t-d7	NAB6	0.413132	2.588999699			1.624576637		
	DIT1t-d7	NAB6	0.410242	2.571828472			1.613801829		
9	DIT1t-d7	PAP1	0.630878	4.27442794	4.384849967	0.099986902	2.682169399	2.75	0.06
	DIT1t-d7	PAP1	0.636531	4.330429773			2.717310102		
	DIT1t-d7	PAP1	0.647604	4.442260269			2.787482846		
	DIT1t-d7	PAP1	0.652467	4.492281886			2.818871011		
10	DIT1t-d8	–	0.18636	1.535889601	1.589017425	0.036029422	0.96375846	1.00	0.02
	DIT1t-d8	–	0.206778	1.609822526			1.01015078		
	DIT1t-d8	–	0.203453	1.597544631			1.002446499		
	DIT1t-d8	–	0.207584	1.612812943			1.012027243		
11	DIT1t-d8	NAB6	0.330385	2.139858223	2.387901302	0.180053597	1.342743947	1.50	0.11
	DIT1t-d8	NAB6	0.398343	2.502320881			1.570186371		
	DIT1t-d8	NAB6	0.404409	2.537517231			1.592271799		
	DIT1t-d8	NAB6	0.375098	2.371908874			1.488353878		
12	DIT1t-d8	PAP1	0.600775	3.988182284	3.936937856	0.231101549	2.502552536	2.47	0.15
	DIT1t-d8	PAP1	0.595017	3.935654809			2.469591964		
	DIT1t-d8	PAP1	0.622375	4.191553367			2.630166266		
	DIT1t-d8	PAP1	0.560189	3.632360966			2.2792775		
13	DIT1t-d9	–	0.220346	1.660909618	1.664798766	0.003561766	1.042207522	1.04	0.00
	DIT1t-d9	–	0.222535	1.669302332			1.047473883		
	DIT1t-d9	–	0.220992	1.663382009			1.043758928		
	DIT1t-d9	–	0.221571	1.665601104			1.045151392		
14	DIT1t-d9	NAB6	0.383665	2.419162267	2.404052996	0.079257947	1.518005005	1.51	0.05
	DIT1t-d9	NAB6	0.360151	2.291664304			1.438001051		
	DIT1t-d9	NAB6	0.385186	2.427649588			1.523330731		
	DIT1t-d9	NAB6	0.394055	2.477735824			1.554759443		
15	DIT1t-d9	PAP1	0.569284	3.709232018	3.741199386	0.049025813	2.327513472	2.35	0.03
	DIT1t-d9	PAP1	0.568004	3.69831586			2.32066367		
	DIT1t-d9	PAP1	0.58052	3.806448865			2.388516267		
	DIT1t-d9	PAP1	0.574124	3.7508008			2.35359755		
16	DIT1t-d10	–	0.215018	1.640657771	1.48221441	0.2224637	1.02949965	0.93	0.14
	DIT1t-d10	–	0.209987	1.621761551			1.017642423		
	DIT1t-d10	–	0.0647375	1.160746813			0.728359356		
	DIT1t-d10	–	0.177736	1.505691504			0.944809395		
17	DIT1t-d10	NAB6	0.438174	2.742672803	2.662321763	0.131173728	1.721005283	1.67	0.08
	DIT1t-d10	NAB6	0.424542	2.657920581			1.66782394		
	DIT1t-d10	NAB6	0.442434	2.769708093			1.737969711		
	DIT1t-d10	NAB6	0.394274	2.478985577			1.555543654		
18	DIT1t-d10	PAP1	0.588488	3.87693036	3.971594651	0.072302113	2.432742842	2.49	0.05
	DIT1t-d10	PAP1	0.600076	3.98176844			2.4985279		
	DIT1t-d10	PAP1	0.599315	3.974797427			2.494153645		
	DIT1t-d10	PAP1	0.607764	4.052882379			2.543151329		
19	DIT1t-d11	–	0.133283	1.359198855	1.416446374	0.039665692	0.852886428	0.89	0.02
	DIT1t-d11	–	0.154199	1.426260977			0.894967374		
	DIT1t-d11	–	0.161574	1.450687938			0.910295097		
	DIT1t-d11	–	0.155226	1.429637726			0.897086257		
20	DIT1t-d11	NAB6	0.353059	2.254545477	2.204380878	0.048936504	1.414709283	1.38	0.03
	DIT1t-d11	NAB6	0.338202	2.178722909			1.367131227		
	DIT1t-d11	NAB6	0.349315	2.235192852			1.402565666		
	DIT1t-d11	NAB6	0.332249	2.149062272			1.348519415		
21	DIT1t-d11	PAP1	0.497903	3.147045341	3.131019911	0.106205383	1.974745821	1.96	0.07
	DIT1t-d11	PAP1	0.479438	3.016046271			1.89254495		

21	DIT1t-d11	PAP1	0.51434	3.26843611			2.050917559		
	DIT1t-d11	PAP1	0.490317	3.092551923			1.940551636		
22	DIT1t-d12	–	0.198892	1.580854865	1.589319594	0.041851289	0.991973804	1.13	0.26
	DIT1t-d12	–	0.213453	1.634756226			1.025796477		
	DIT1t-d12	–	0.190989	1.552347691			0.97408578		
23	DIT1t-d12	NAB6	0.383624	2.418933895	2.45509173	0.030840885	1.517861703	1.54	0.02
	DIT1t-d12	NAB6	0.387704	2.441765763			1.532188518		
	DIT1t-d12	NAB6	0.395907	2.48832441			1.561403697		
	DIT1t-d12	NAB6	0.392933	2.471342854			1.550747906		
24	DIT1t-d12	PAP1	0.5751	3.75923954	3.802520431	0.079891624	2.358892792	2.39	0.05
	DIT1t-d12	PAP1	0.587643	3.869394411			2.428014094		
	DIT1t-d12	PAP1	0.587695	3.869857738			2.428304828		
	DIT1t-d12	PAP1	0.56956	3.711590035			2.328993108		

	1	4	significant
Mean	1	0.550436076	
Variance	0.000491896	2.09613E-05	
Observations	4	4	
Pearson Correlation	-0.43359438		
Hypothesized Mean Differe	0		
df	3		
t Stat	36.6788226		
P(T<=t) one-tail	2.22861E-05		
t Critical one-tail	4.540702859		
P(T<=t) two-tail	4.45722E-05		
t Critical two-tail	5.84090931		

	2	5	significant
Mean	1.559370115	0.67600076	
Variance	0.001276313	0.000109492	
Observations	4	4	
Pearson Correlation	0.865680083		
Hypothesized Mean Differe	0		
df	3		
t Stat	65.00918366		
P(T<=t) one-tail	4.01002E-06		
t Critical one-tail	4.540702859		
P(T<=t) two-tail	8.02004E-06		
t Critical two-tail	5.84090931		

	3	6	significant
Mean	2.503164465	0.701606327	
Variance	0.008204824	0.000227423	
Observations	4	4	
Pearson Correlation	0.810215939		
Hypothesized Mean Differe	0		
df	3		
t Stat	45.69065301		
P(T<=t) one-tail	1.15401E-05		
t Critical one-tail	4.540702859		
P(T<=t) two-tail	2.30802E-05		
t Critical two-tail	5.84090931		

Population	Genetic Background	Overexpression	Average log10 value (a.u.) of fluorecent intensity (FI) from 3000 cells	Average value of FI	Mean value of Average FI	Standard Deviatoin of Average FI	Relative FI (DIT1t-- Mean value as a standard)	Mean value of relative FI	SD of relative FI
1	DIT1t	–	0.208579	1.61651225	1.593645778	0.035345049	1.014348528	1.00	0.02
	DIT1t	–	0.211901	1.628924667			1.022137221		
	DIT1t	–	0.191041	1.552533572			0.974202418		
	DIT1t	–	0.197725	1.576612625			0.989311832		
2	DIT1t	NAB6	0.382478	2.412559317	2.485083602	0.056933824	1.513861706	1.56	0.04
	DIT1t	NAB6	0.406602	2.550363005			1.60033242		
	DIT1t	NAB6	0.394329	2.479299541			1.555740664		
	DIT1t	NAB6	0.397612	2.498112543			1.567545672		
3	DIT1t	PAP1	0.621917	4.187135352	3.989157483	0.144353204	2.627393997	2.50	0.09
	DIT1t	PAP1	0.598734	3.96948349			2.490819192		
	DIT1t	PAP1	0.58441	3.840696592			2.410006442		
	DIT1t	PAP1	0.59762	3.959314496			2.484438229		
4	DIT1t-d2	–	-0.0554647	0.880106645	0.85532395	0.021929232	0.552259892	0.54	0.01
	DIT1t-d2	–	-0.0761072	0.839252802			0.526624432		
	DIT1t-d2	–	-0.0618398	0.867281735			0.544212363		
	DIT1t-d2	–	-0.0784932	0.834654617			0.523739107		
5	DIT1t-d2	NAB6	0.0293185	1.069839183	1.102364382	0.037898534	0.671315544	0.69	0.02
	DIT1t-d2	NAB6	0.0386463	1.093065784			0.685890051		
	DIT1t-d2	NAB6	0.0372029	1.089438954			0.683614244		
	DIT1t-d2	NAB6	0.063376	1.157113605			0.726079547		
6	DIT1t-d2	PAP1	0.061249	1.151460382	1.059846626	0.062298025	0.722532195	0.67	0.04
	DIT1t-d2	PAP1	0.00546878	1.012671949			0.635443561		
	DIT1t-d2	PAP1	0.0178423	1.041939014			0.653808411		
	DIT1t-d2	PAP1	0.0142328	1.033315157			0.64839701		
7	DIT1t-d13	–	0.182472	1.52220099	1.608322056	0.259170865	0.955168966	1.01	0.16
	DIT1t-d13	–	0.159246	1.442932449			0.905428589		
	DIT1t-d13	–	0.299723	1.99399011			1.251212871		
	DIT1t-d13	–	0.168546	1.474164675			0.925026561		
8	DIT1t-d13	NAB6	0.341039	2.19300186	2.05922842	0.094647704	1.376091155	1.29	0.06
	DIT1t-d13	NAB6	0.302213	2.005455363			1.258407226		
	DIT1t-d13	NAB6	0.296935	1.981230477			1.243206303		
	DIT1t-d13	NAB6	0.313282	2.057225979			1.290892874		
9	DIT1t-d13	PAP1	0.508664	3.225997303	3.176454356	0.330723531	2.024287547	1.99	0.21
	DIT1t-d13	PAP1	0.474836	2.98425548			1.872596483		
	DIT1t-d13	PAP1	0.558827	3.620987287			2.272140607		
	DIT1t-d13	PAP1	0.458574	2.874577355			1.803774335		
10	DIT1t-d14	–	0.0976372	1.252094766	1.236915006	0.011472474	0.785679467	0.78	0.01
	DIT1t-d14	–	0.0921541	1.23638606			0.77582238		
	DIT1t-d14	–	0.0916388	1.234919931			0.774902395		
	DIT1t-d14	–	0.0878734	1.224259267			0.768212914		
11	DIT1t-d14	NAB6	0.26563	1.843444216	1.836342392	0.016785048	1.156746525	1.15	0.01
	DIT1t-d14	NAB6	0.258351	1.812804623			1.137520425		
	DIT1t-d14	NAB6	0.267601	1.851829508			1.162008228		
	DIT1t-d14	NAB6	0.264178	1.837291221			1.15288557		
12	DIT1t-d14	PAP1	0.438202	2.742849636	2.603011677	0.196152708	1.721116243	1.63	0.12
	DIT1t-d14	PAP1	0.445085	2.786666521			1.748610989		
	DIT1t-d14	PAP1	0.399905	2.511337028			1.575843931		
	DIT1t-d14	PAP1	0.374967	2.371193523			1.487905001		
13	DIT1t-d15	–	-0.013561	0.96925712	0.988897155	0.018810492	0.608201103	0.62	0.01
	DIT1t-d15	–	0.00426522	1.009869417			0.633684995		
	DIT1t-d15	–	-0.00040324	0.999071927			0.626909656		
	DIT1t-d15	–	-0.00993204	0.977390155			0.613304517		
14	DIT1t-d15	NAB6	0.132995	1.358297809	1.373896232	0.029764874	0.852321028	0.86	0.02
	DIT1t-d15	NAB6	0.13507	1.364803099			0.856403046		
	DIT1t-d15	NAB6	0.131749	1.354406408			0.849879206		
	DIT1t-d15	NAB6	0.1517	1.41807761			0.889832376		
15	DIT1t-d15	PAP1	0.139308	1.378186527	1.36116291	0.02726688	0.86480104	0.85	0.02
	DIT1t-d15	PAP1	0.143005	1.389968634			0.872194218		
	DIT1t-d15	PAP1	0.124745	1.332738673			0.836282875		
	DIT1t-d15	PAP1	0.128321	1.343757806			0.843197293		
16	DIT1t-d16	–	0.0330577	1.07909008	1.060662438	0.017271954	0.677120408	0.67	0.01
	DIT1t-d16	–	0.0160969	1.037759935			0.651186073		
	DIT1t-d16	–	0.0278095	1.066128369			0.668987038		
	DIT1t-d16	–	0.0251712	1.059671368			0.664935321		
17	DIT1t-d16	NAB6	0.1754	1.497614375	1.43300022	0.046015254	0.939741061	0.90	0.03
	DIT1t-d16	NAB6	0.143298	1.390906703			0.872782849		
	DIT1t-d16	NAB6	0.155473	1.430451047			0.897596609		
	DIT1t-d16	NAB6	0.150151	1.413028756			0.886664261		
18	DIT1t-d16	PAP1	0.171229	1.483300011	1.43328627	0.034099037	0.930758912	0.90	0.02
	DIT1t-d16	PAP1	0.149252	1.410106778			0.884830743		
	DIT1t-d16	PAP1	0.15429	1.426559861			0.895154921		
	DIT1t-d16	PAP1	0.150197	1.41317843			0.88675818		
19	DIT1t-d17	–	0.164625	1.460915174	1.440745157	0.074750327	0.916712606	0.90	0.05
	DIT1t-d17	–	0.182754	1.52318972			0.955789386		
	DIT1t-d17	–	0.157086	1.43577372			0.900936544		
	DIT1t-d17	–	0.128109	1.343102013			0.842785788		
20	DIT1t-d17	NAB6	0.364205	2.313156412	2.210675944	0.135924872	1.451487177	1.39	0.09
	DIT1t-d17	NAB6	0.34406	2.208309801			1.385696766		
	DIT1t-d17	NAB6	0.305214	2.019361165			1.267133006		
	DIT1t-d17	NAB6	0.362082	2.301876398			1.444409058		
21	DIT1t-d17	PAP1	0.45924	2.878988963	3.046004832	0.11184802	1.806542584	1.91	0.07
	DIT1t-d17	PAP1	0.491672	3.102215761			1.946615618		

DIT1t-d17	PAP1	0.493373	3.114390027	1.954254872
DIT1t-d17	PAP1	0.489737	3.088424578	1.93796176

	1	13	significant
Mean	1	0.620525068	
Variance	0.000491896	0.000139321	
Observations	4	4	
Pearson Correlation	0.034329623		
Hypothesized Mean Differe	0		
df	3		
t Stat	30.64761293		
P(T<=t) one-tail	3.81583E-05		
t Critical one-tail	4.540702859		
P(T<=t) two-tail	7.63166E-05		
t Critical two-tail	5.84090931		

	2	14	significant
Mean	1.559370115	0.862108914	
Variance	0.001276313	0.000348839	
Observations	4	4	
Pearson Correlation	0.241161488		
Hypothesized Mean Differe	0		
df	3		
t Stat	38.62780348		
P(T<=t) one-tail	1.90851E-05		
t Critical one-tail	4.540702859		
P(T<=t) two-tail	3.81702E-05		
t Critical two-tail	5.84090931		

	3	15	significant
Mean	2.503164465	0.854118856	
Variance	0.008204824	0.000292744	
Observations	4	4	
Pearson Correlation	0.638784382		
Hypothesized Mean Differe	0		
df	3		
t Stat	40.8525808		
P(T<=t) one-tail	1.61379E-05		
t Critical one-tail	4.540702859		
P(T<=t) two-tail	3.22757E-05		
t Critical two-tail	5.84090931		

	1	16	significant
Mean	1	0.66555721	
Variance	0.000491896	0.000117463	
Observations	4	4	
Pearson Correlation	-0.324573582		
Hypothesized Mean Differe	0		
df	3		
t Stat	24.17734302		
P(T<=t) one-tail	7.75438E-05		
t Critical one-tail	4.540702859		
P(T<=t) two-tail	0.000155088		
t Critical two-tail	5.84090931		

	2	17	significant
Mean	1.559370115	0.899196195	
Variance	0.001276313	0.00083372	
Observations	4	4	
Pearson Correlation	-0.977088073		
Hypothesized Mean Differe	0		
df	3		
t Stat	20.55565773		
P(T<=t) one-tail	0.000125881		
t Critical one-tail	4.540702859		
P(T<=t) two-tail	0.000251761		
t Critical two-tail	5.84090931		

	3	18	significant
Mean	2.503164465	0.899375689	
Variance	0.008204824	0.000457826	
Observations	4	4	
Pearson Correlation	0.809670346		
Hypothesized Mean Differe	0		
df	3		
t Stat	43.1563521		
P(T<=t) one-tail	1.3692E-05		
t Critical one-tail	4.540702859		
P(T<=t) two-tail	2.73841E-05		
t Critical two-tail	5.84090931		

	13	16	no difference
Mean	0.620525068	0.66555721	
Variance	0.000139321	0.000117463	
Observations	4	4	
Pearson Correlation	-0.795354857		
Hypothesized Mean Differe	0		
df	3		
t Stat	-4.198008188		
P(T<=t) one-tail	0.012331518		
t Critical one-tail	4.540702859		
P(T<=t) two-tail	0.024663036		
t Critical two-tail	5.84090931		

	14	17	no difference
Mean	0.862108914	0.899196195	
Variance	0.000348839	0.00083372	
Observations	4	4	
Pearson Correlation	-0.354789759		
Hypothesized Mean Differe	0		
df	3		
t Stat	-1.874848915		
P(T<=t) one-tail	0.078747797		
t Critical one-tail	4.540702859		
P(T<=t) two-tail	0.157495595		
t Critical two-tail	5.84090931		

	15	18	no difference
Mean	0.854118856	0.899375689	
Variance	0.000292744	0.000457826	
Observations	4	4	
Pearson Correlation	0.259878411		
Hypothesized Mean Differe	0		
df	3		
t Stat	-3.823912962		
P(T<=t) one-tail	0.015744905		
t Critical one-tail	4.540702859		
P(T<=t) two-tail	0.03148981		
t Critical two-tail	5.84090931		

Population	Genetic Background	Overexpression	Average log10 value (a.u.) of fluorecent intensity (FI) from 3000 cells	Average value of FI	Mean value of Average FI	Standard Deviatoin of Average FI	Relative FI (DIT1t-- Mean value as a standard)	Mean value of relative FI	SD of relative FI
1	DIT1t	–	0.212343	1.630583337	1.583269315	0.054430177	1.029883749	1.00	0.03
	DIT1t	–	0.208628	1.616694646			1.021111589		
	DIT1t	–	0.178744	1.509190282			0.95321135		
	DIT1t	–	0.197724	1.576608995			0.995793312		
4	DIT1t-d2	–	-0.0472242	0.896965626	0.898016964	0.004337292	0.566527512	0.57	0.00
	DIT1t-d2	–	-0.0437386	0.90419354			0.571092695		
	DIT1t-d2	–	-0.048647	0.894031869			0.564674538		
	DIT1t-d2	–	-0.0472672	0.896876821			0.566471422		
5	DIT1t-d2	NAB6	0.0210695	1.0497104	1.078077133	0.019636439	0.663001796	0.68	0.01
	DIT1t-d2	NAB6	0.0391978	1.094454724			0.691262512		
	DIT1t-d2	NAB6	0.0360383	1.086521439			0.686251813		
	DIT1t-d2	NAB6	0.0340755	1.08162197			0.683157287		
6	DIT1t-d2	PAP1	0.0494476	1.120592212	1.100330675	0.019198711	0.707771067	0.69	0.01
	DIT1t-d2	PAP1	0.0311188	1.074283239			0.678522112		
	DIT1t-d2	PAP1	0.0426119	1.103092419			0.696718119		
	DIT1t-d2	PAP1	0.0427152	1.103354829			0.696883858		
7	DIT1t-d18	–	0.21418	1.637495066	1.599030783	0.035859818	1.034249227	1.01	0.02
	DIT1t-d18	–	0.195828	1.569740994			0.991455452		
	DIT1t-d18	–	0.209952	1.621630858			1.024229323		
	DIT1t-d18	–	0.19514	1.567256212			0.989886053		
8	DIT1t-d18	NAB6	0.38648	2.434893666	2.402703731	0.061611899	1.53788976	1.52	0.04
	DIT1t-d18	NAB6	0.386642	2.435802097			1.538463528		
	DIT1t-d18	NAB6	0.385561	2.429746695			1.534638909		
	DIT1t-d18	NAB6	0.363682	2.310372465			1.45924161		
9	DIT1t-d18	PAP1	0.530797	3.394665604	3.395195021	0.119769225	2.14408602	2.14	0.08
	DIT1t-d18	PAP1	0.531729	3.401958411			2.148692189		
	DIT1t-d18	PAP1	0.511282	3.245502889			2.04987418		
	DIT1t-d18	PAP1	0.548838	3.538653181			2.235029219		
10	DIT1t-d19	–	0.218406	1.653506856	1.567319437	0.074166908	1.044362346	0.99	0.05
	DIT1t-d19	–	0.201727	1.59120817			1.005014216		
	DIT1t-d19	–	0.189559	1.547244687			0.977246683		
	DIT1t-d19	–	0.169474	1.477318036			0.933080697		
11	DIT1t-d19	NAB6	0.382061	2.410243942	2.420675599	0.117412482	1.522320883	1.53	0.07
	DIT1t-d19	NAB6	0.403118	2.529985313			1.59795007		
	DIT1t-d19	NAB6	0.394752	2.481715539			1.567462664		
	DIT1t-d19	NAB6	0.354254	2.260757603			1.427904641		
12	DIT1t-d19	PAP1	0.504746	3.197024762	3.316550125	0.195493241	2.019255178	2.09	0.12
	DIT1t-d19	PAP1	0.556951	3.605379624			2.27717394		
	DIT1t-d19	PAP1	0.504574	3.195758848			2.018455621		
	DIT1t-d19	PAP1	0.514287	3.268037264			2.064106993		
13	DIT1t-d20	–	0.177807	1.50593768	1.553562814	0.049596413	0.951156993	0.98	0.03
	DIT1t-d20	–	0.180628	1.515751477			0.957355431		
	DIT1t-d20	–	0.204194	1.600272713			1.010739423		
	DIT1t-d20	–	0.202022	1.592289385			1.005697117		
14	DIT1t-d20	NAB6	0.362671	2.305000373	2.319596902	0.048787711	1.455848573	1.47	0.03
	DIT1t-d20	NAB6	0.378454	2.390308743			1.509729722		
	DIT1t-d20	NAB6	0.362638	2.304825233			1.455737954		
	DIT1t-d20	NAB6	0.357602	2.278253258			1.438954975		
15	DIT1t-d20	PAP1	0.515418	3.276559056	3.085089419	0.166289978	2.069489395	1.95	0.11
	DIT1t-d20	PAP1	0.501317	3.171881837			2.003374793		
	DIT1t-d20	PAP1	0.468499	2.941026922			1.857565794		
	DIT1t-d20	PAP1	0.469953	2.95088986			1.86379527		
16	DIT1t-d21	–	0.218854	1.655213425	1.683257476	0.032790533	1.045440222	1.06	0.02
	DIT1t-d21	–	0.226273	1.683732132			1.063452766		
	DIT1t-d21	–	0.221403	1.664956917			1.051594256		
	DIT1t-d21	–	0.237827	1.729127429			1.092124639		
17	DIT1t-d21	NAB6	0.396395	2.491122016	2.45016898	0.05519152	1.573403838	1.55	0.03
	DIT1t-d21	NAB6	0.374522	2.368765123			1.496122675		
	DIT1t-d21	NAB6	0.392262	2.467527492			1.55850143		
	DIT1t-d21	NAB6	0.39327	2.473261289			1.562122922		
18	DIT1t-d21	PAP1	0.558249	3.616171341	3.674886427	0.203575061	2.283990037	2.32	0.13
	DIT1t-d21	PAP1	0.586134	3.85597314			2.435449928		
	DIT1t-d21	PAP1	0.581432	3.814450645			2.409224135		
	DIT1t-d21	PAP1	0.53313	3.412950583			2.155634894		

Population	Genetic Background	Overexpression	Average log10 value (a.u.) of fluorecent intensity (FI) from 3000 cells	Average value of FI	Mean value of Average FI	Standard Deviatoin of Average FI	Relative FI (DIT1t−-Mean value as a standard)	Mean value of relative FI	SD of relative FI
1	PGK1t	−	0.360468	2.293337645	2.205630953	0.079600563	1.039764899	1.00	0.04
	PGK1t	−	0.32334	2.105426088			0.954568617		
	PGK1t	−	0.339931	2.187414064			0.991740735		
	PGK1t	−	0.349539	2.236346015			1.013925749		
2	PGK1t	NAB6	0.324054	2.108890352	2.089803887	0.013750111	0.956139262	0.95	0.01
	PGK1t	NAB6	0.320065	2.089608855			0.947397321		
	PGK1t	NAB6	0.317408	2.076863721			0.941618868		
	PGK1t	NAB6	0.318867	2.083852618			0.944787529		
3	PGK1t	PAP1	0.327622	2.126287568	2.144175832	0.067707271	0.964026899	0.97	0.03
	PGK1t	PAP1	0.321888	2.098398659			0.951382486		
	PGK1t	PAP1	0.351069	2.244238456			1.017504063		
	PGK1t	PAP1	0.323825	2.107778644			0.955635231		
4	PGK1t	NAB6+PAP1	0.27317	1.875728599	1.890628346	0.023276427	0.850427219	0.86	0.01
	PGK1t	NAB6+PAP1	0.27116	1.867067417			0.84650037		
	PGK1t	NAB6+PAP1	0.282674	1.917229046			0.8692429		
	PGK1t	NAB6+PAP1	0.279322	1.902488324			0.862559678		
5	DIT1t-inserted PGK1t	−	0.390199	2.455833956	2.45470374	0.017263231	1.11343829	1.11	0.01
	DIT1t-inserted PGK1t	−	0.39211	2.466664026			1.118348481		
	DIT1t-inserted PGK1t	−	0.39206	2.466380057			1.118219734		
	DIT1t-inserted PGK1t	−	0.385595	2.429936922			1.101696963		
6	DIT1t-inserted PGK1t	NAB6	0.482955	3.040569957	2.935288641	0.079029212	1.378548824	1.33	0.04
	DIT1t-inserted PGK1t	NAB6	0.468137	2.938576492			1.332306517		
	DIT1t-inserted PGK1t	NAB6	0.455064	2.85143844			1.292799431		
	DIT1t-inserted PGK1t	NAB6	0.463978	2.910569674			1.319608645		
7	DIT1t-inserted PGK1t	PAP1	0.406562	2.550128119	2.581024289	0.039551173	1.156189849	1.17	0.02
	DIT1t-inserted PGK1t	PAP1	0.420315	2.632176455			1.193389334		
	DIT1t-inserted PGK1t	PAP1	0.413686	2.592304418			1.175311951		
	DIT1t-inserted PGK1t	PAP1	0.406453	2.549488163			1.155899703		
8	DIT1t-inserted PGK1t	NAB6+PAP1	0.488099	3.076798109	2.986717205	0.110532832	1.394974125	1.35	0.05
	DIT1t-inserted PGK1t	NAB6+PAP1	0.487994	3.076054317			1.394636901		
	DIT1t-inserted PGK1t	NAB6+PAP1	0.454811	2.849777807			1.292046524		
	DIT1t-inserted PGK1t	NAB6+PAP1	0.468973	2.944238585			1.334873625		
9	DIT1t-substituted PGK1t	−	0.409363	2.566628427	2.628866403	0.132551193	1.163670841	1.19	0.06
	DIT1t-substituted PGK1t	−	0.451425	2.827645752			1.282012182		
	DIT1t-substituted PGK1t	−	0.408454	2.561261964			1.161237767		
	DIT1t-substituted PGK1t	−	0.408228	2.55992947			1.160633635		
10	DIT1t-substituted PGK1t	NAB6	0.515859	3.279887895	3.219816414	0.073110497	1.487051989	1.46	0.03
	DIT1t-substituted PGK1t	NAB6	0.510699	3.241149025			1.469488366		
	DIT1t-substituted PGK1t	NAB6	0.49671	3.13841232			1.422909085		
11	DIT1t-substituted PGK1t	PAP1	0.430805	2.696528408	2.748696323	0.037047958	1.222565545	1.25	0.02
	DIT1t-substituted PGK1t	PAP1	0.439753	2.752662713			1.248015997		
	DIT1t-substituted PGK1t	PAP1	0.444553	2.783255013			1.261886087		
	DIT1t-substituted PGK1t	PAP1	0.441277	2.762339159			1.252403152		
12	DIT1t-substituted PGK1t	NAB6+PAP1	0.527027	3.365324909	3.438270923	0.154968268	1.52578785	1.56	0.07
	DIT1t-substituted PGK1t	NAB6+PAP1	0.518471	3.299673736			1.496022592		
	DIT1t-substituted PGK1t	NAB6+PAP1	0.535538	3.431926672			1.55598409		
	DIT1t-substituted PGK1t	NAB6+PAP1	0.563025	3.656158375			1.657647382		

t-Test: Two-Sample Assuming Equal Variances (P<0.01)

Population	1	2	no difference
Mean	2.205630953	2.089803887	
Variance	0.00633625	0.000189066	
Observation	4	4	
Pooled Varia	0.003262658		
Hypothesize	0		
df	6		
t Stat	2.867736914		
P(T<=t) one	0.014255702		
t Critical on	3.142668403		
P(T<=t) two	0.028511403		
t Critical tw	3.707428021		

Population	1	3	no difference
Mean	2.205630953	2.144175832	
Variance	0.00633625	0.004584275	
Observation	4	4	
Pooled Varia	0.005460262		
Hypothesize	0		
df	6		
t Stat	1.176159804		
P(T<=t) one	0.142039336		
t Critical on	3.142668403		
P(T<=t) two	0.284078673		
t Critical tw	3.707428021		

Population	1	4	significant
Mean	2.205630953	1.890628346	
Variance	0.00633625	0.000541792	
Observation	4	4	
Pooled Varia	0.003439021		
Hypothesize	0		
df	6		
t Stat	7.59646852		
P(T<=t) one	0.000135424		
t Critical on	3.142668403		

P(T<=t) two	0.000270848
t Critical tw	3.707428021

Population	5	6	significant
Mean	2.45470374	2.935288641	
Variance	0.000298019	0.006245616	
Observation	4	4	
Pooled Varia	0.003271818		
Hypothesize	0		
df	6		
t Stat	-11.88202792		
P(T<=t) one	1.07512E-05		
t Critical one	3.142668403		
P(T<=t) two	2.15024E-05		
t Critical tw	3.707428021		

Population	5	7	significant
Mean	2.45470374	2.581024289	
Variance	0.000298019	0.001564295	
Observation	4	4	
Pooled Varia	0.000931157		
Hypothesize	0		
df	6		
t Stat	-5.85433392		
P(T<=t) one	0.000548431		
t Critical one	3.142668403		
P(T<=t) two	0.001096862		
t Critical tw	3.707428021		

Population	5	8	significant
Mean	2.45470374	2.986717205	
Variance	0.000298019	0.012217507	
Observation	4	4	
Pooled Varia	0.006257763		
Hypothesize	0		
df	6		
t Stat	-9.511041234		
P(T<=t) one	3.85153E-05		
t Critical one	3.142668403		
P(T<=t) two	7.70306E-05		
t Critical tw	3.707428021		

Population	6	8	no difference
Mean	1.330815854	1.354132794	
Variance	0.001283836	0.002511406	
Observation	4	4	
Pooled Varia	0.851332303		
Hypothesize	0		
df	3		
t Stat	-1.71671953		
P(T<=t) one	0.092266363		
t Critical one	4.540702859		
P(T<=t) two	0.184532726		
t Critical tw	5.84090931		

Population	9	10	significant
Mean	2.628866403	3.219816414	
Variance	0.017569819	0.005345145	
Observation	4	3	
Pooled Varia	0.012679949		
Hypothesize	0		
df	5		
t Stat	-6.871215853		
P(T<=t) one	0.000499203		
t Critical one	3.364929999		
P(T<=t) two	0.000998406		
t Critical tw	4.032142984		

Population	9	11	no difference
Mean	2.628866403	2.748696323	
Variance	0.017569819	0.001372551	
Observation	4	4	
Pooled Varia	0.009471185		
Hypothesize	0		
df	6		
t Stat	-1.741318079		
P(T<=t) one	0.066134555		
t Critical one	3.142668403		
P(T<=t) two	0.132269109		
t Critical tw	3.707428021		

Population	9	12	significant
Mean	2.628866403	3.438270923	
Variance	0.017569819	0.024015164	
Observation	4	4	
Pooled Varia	0.020792491		
Hypothesize	0		

df	6
t Stat	−7.938297255
P(T<=t) one·	0.000106196
t Critical on	3.142668403
P(T<=t) two·	0.000212391
t Critical tw	3.707428021

Population	10	12	no difference
Mean	1.45981648	1.525931511	
Variance	0.001098737	0.000898861	
Observation	3	3	
Pooled Varia	−0.705559358		
Hypothesize	0		
df	2		
t Stat	−1.963923896		
P(T<=t) one·	0.094251283		
t Critical on	6.964556734		
P(T<=t) two·	0.188502566		
t Critical tw	9.924843201		

Population	Genetic Background	Overexpression	Average log10 value (a.u.) of fluorecent intensity (FI) from 3000 cells	Average value of FI	Mean value of Average FI	Standard Deviatoin of Average FI	Relative FI (DIT1t-- Mean value as a standard)	Mean value of relative FI	SD of relative FI
1	DIT1t	–	0.279053	1.901310296	1.916657802	0.019635042	0.991992568	1.00	0.01
	DIT1t	–	0.285204	1.928430537			1.006142325		
	DIT1t	–	0.287386	1.938143817			1.011210146		
	DIT1t	–	0.278467	1.89874656			0.990654961		
2	m1	–	-0.0126832	0.971218173	0.965604578	0.055348029	0.506724868	0.50	0.03
	m1	–	0.0163975	1.038478477			0.541817363		
	m1	–	-0.024129	0.945956139			0.493544616		
	m1	–	-0.042505	0.906765525			0.473097245		
3	m1	NAB6	0.104618	1.272383416	1.72431916	0.467721955	0.663855287	0.90	0.24
	m1	NAB6	0.136477	1.369231873			0.714385151		
	m1	NAB6	0.325366	2.115270925			1.103624717		
	m1	NAB6	0.330493	2.140390427			1.116730605		
4	m1	PAP1	0.390887	2.459727519	2.457939957	0.010580681	1.283342032	1.28	0.01
	m1	PAP1	0.392715	2.470102641			1.288755164		
	m1	PAP1	0.390507	2.457576242			1.282219622		
	m1	PAP1	0.388164	2.444353425			1.275320729		
5	m1	NAB6+PAP1	0.540655	3.472601912	3.298348848	0.150874439	1.811800681	1.72	0.08
	m1	NAB6+PAP1	0.501829	3.175623448			1.656854679		
	m1	NAB6+PAP1	0.528448	3.376354196			1.76158425		
	m1	NAB6+PAP1	0.500897	3.168815838			1.653302866		
6	m2	–	0.259128	1.81605083	1.867250217	0.038162616	0.947509163	0.97	0.02
	m2	–	0.278238	1.89774563			0.990132734		
	m2	–	0.277603	1.894972885			0.988686078		
	m2	–	0.269567	1.860231525			0.970560067		
7	m2	NAB6	0.46689	2.93015099	2.834125937	0.084186029	1.5287815	1.48	0.04
	m2	NAB6	0.447481	2.80208303			1.461963125		
	m2	NAB6	0.457745	2.869095472			1.4969263		
	m2	NAB6	0.436985	2.735174255			1.427054037		
8	m2	PAP1	0.632464	4.290066261	4.280789838	0.127397649	2.238305792	2.23	0.07
	m2	PAP1	0.612716	4.099359442			2.138806122		
	m2	PAP1	0.642238	4.387710851			2.289251032		
	m2	PAP1	0.638092	4.346022796			2.267500641		
9	m2	NAB6+PAP1	0.702502	5.040829405	4.89882034	0.404830009	2.630010114	2.56	0.21
	m2	NAB6+PAP1	0.688039	4.875722725			2.5438671		
	m2	NAB6+PAP1	0.639263	4.357756908			2.273622815		
	m2	NAB6+PAP1	0.725991	5.320972324			2.776172313		
10	m3	–	0.193537	1.561482064	1.588628289	0.050690262	0.814690062	0.83	0.03
	m3	–	0.203049	1.596059215			0.832730398		
	m3	–	0.219181	1.656460179			0.86424409		
	m3	–	0.187665	1.540511696			0.80374895		
11	m3	NAB6	0.397596	2.498020511	2.504619192	0.034872418	1.303321077	1.31	0.02
	m3	NAB6	0.406831	2.551708146			1.331332146		
	m3	NAB6	0.398152	2.501220618			1.304990706		
	m3	NAB6	0.392262	2.467527492			1.287411602		
12	m3	PAP1	0.546867	3.522629762	3.482113173	0.07281143	1.837902289	1.82	0.04
	m3	PAP1	0.547938	3.531327529			1.842440275		
	m3	PAP1	0.52824	3.37473752			1.760740762		
	m3	PAP1	0.544038	3.49975788			1.825969078		
13	m3	NAB6+PAP1	0.625393	4.22078276	4.355087689	0.096929132	2.202157712	2.27	0.05
	m3	NAB6+PAP1	0.638928	4.354396778			2.271869696		
	m3	NAB6+PAP1	0.643436	4.399831048			2.295574641		
	m3	NAB6+PAP1	0.647905	4.44534017			2.319318641		
14	m4	–	0.067717	1.168737557	1.165612558	0.012826476	0.609778937	0.61	0.01
	m4	–	0.0684984	1.170842289			0.610877063		
	m4	–	0.0595321	1.14691729			0.598394397		
	m4	–	0.07039	1.175953096			0.613543584		
15	m4	NAB6	0.242456	1.747656192	1.706747929	0.051047257	0.911824839	0.89	0.03
	m4	NAB6	0.217288	1.649255724			0.860485227		
	m4	NAB6	0.243482	1.751789827			0.913981528		
	m4	NAB6	0.224867	1.678289973			0.875633601		
16	m4	PAP1	0.235442	1.719657666	1.684431201	0.037154674	0.897216845	0.88	0.02
	m4	PAP1	0.216778	1.647320109			0.859475336		
	m4	PAP1	0.21953	1.657791849			0.864938878		
	m4	PAP1	0.233746	1.712955181			0.89371988		
17	m4	NAB6+PAP1	0.3651	2.317928311	2.314522769	0.004113678	1.209359495	1.21	0.00
	m4	NAB6+PAP1	0.363603	2.309952237			1.205198045		
	m4	NAB6+PAP1	0.36468	2.315687759			1.208190506		

Population	Genetic Background	Overexpression	Average log10 value (a.u.) of fluorecent intensity (FI) from 3000 cells	Average value of FI	Mean value of Average FI	Standard Deviatoin of Average FI	Relative FI (DIT1t--Mean value as a standard)	Mean value of relative FI	SD of relative FI
1	DIT1t	−	0.248352	1.771544231	1.83977389	0.055368251	0.962914106	1.00	0.03
	DIT1t	−	0.280303	1.90679059			1.036426596		
	DIT1t	−	0.266082	1.845363813			1.003038375		
	DIT1t	−	0.26373	1.835396926			0.997620923		
2	m5	−	0.0234964	1.055592753	1.044441464	0.015416633	0.573762221	0.57	0.01
	m5	−	0.0139022	1.032528861			0.561225957		
	m5	−	0.0127871	1.029881128			0.559786794		
	m5	−	0.0252088	1.059763115			0.576029001		
3	m5	NAB6	0.182037	1.520677079	1.455388691	0.067592004	0.826556507	0.79	0.04
	m5	NAB6	0.134924	1.36434436			0.74158263		
	m5	NAB6	0.160764	1.44798479			0.787044972		
	m5	NAB6	0.172763	1.488548536			0.809093196		
4	m5	PAP1	0.139941	1.380196749	1.371109223	0.056994991	0.750199118	0.75	0.03
	m5	PAP1	0.122475	1.325790797			0.720627031		
	m5	PAP1	0.160824	1.44818485			0.787153714		
	m5	PAP1	0.123938	1.330264495			0.723058688		
5	m5	NAB6+PAP1	0.241838	1.74517105	1.74432494	0.012705821	0.948579094	0.95	0.01
	m5	NAB6+PAP1	0.237774	1.728916424			0.939743973		
	m5	NAB6+PAP1	0.245507	1.759977031			0.956626812		
	m5	NAB6+PAP1	0.241356	1.743235253			0.947526901		
6	m6	−	0.0684708	1.170767883	1.119861659	0.039069435	0.636365093	0.61	0.02
	m6	−	0.053181	1.130266875			0.614350971		
	m6	−	0.0371269	1.089248323			0.59205554		
	m6	−	0.0370931	1.089163553			0.592009463		
7	m6	NAB6	0.212269	1.630305524	1.628725106	0.001508178	0.886144505	0.89	0.00
	m6	NAB6	0.211806	1.628568386			0.885200293		
	m6	NAB6	0.211468	1.627301407			0.884511633		
8	m6	PAP1	0.201546	1.590545144	1.53792791	0.03988242	0.864532947	0.84	0.02
	m6	PAP1	0.186902	1.53780759			0.835867711		
	m6	PAP1	0.18451	1.529360961			0.831276587		
	m6	PAP1	0.17435	1.493997945			0.812055195		
9	m6	NAB6+PAP1	0.311588	2.049217236	1.99535378	0.064579265	1.113841895	1.08	0.04
	m6	NAB6+PAP1	0.299826	1.994463074			1.084080541		
	m6	NAB6+PAP1	0.279852	1.904811481			1.035350861		
	m6	NAB6+PAP1	0.308121	2.03292333			1.104985423		
10	m7	−	0.0546957	1.134215819	1.133734346	0.029783294	0.616497399	0.62	0.02
	m7	−	0.0536578	1.131508446			0.61502582		
	m7	−	0.0685708	1.171037493			0.636511638		
	m7	−	0.0406718	1.098175625			0.59690793		
11	m7	NAB6	0.136898	1.370559834	1.395820834	0.040659272	0.74496102	0.76	0.02
	m7	NAB6	0.154152	1.426106634			0.775153208		
	m7	NAB6	0.131018	1.352128603			0.734942816		
	m7	NAB6	0.156697	1.434488266			0.779709003		
12	m7	PAP1	0.169695	1.478069992	1.457823058	0.030564302	0.803397635	0.79	0.02
	m7	PAP1	0.157014	1.435535709			0.780278336		
	m7	PAP1	0.173074	1.489614873			0.809672798		
	m7	PAP1	0.15475	1.428071658			0.776221288		
13	m7	NAB6+PAP1	0.21822	1.652798842	1.601014362	0.068567936	0.898370638	0.87	0.04
	m7	NAB6+PAP1	0.19764	1.576304081			0.856792288		
	m7	NAB6+PAP1	0.180472	1.515207112			0.823583333		
	m7	NAB6+PAP1	0.220042	1.659747411			0.902147498		

Population	Genetic Background	Overexpression	Average log10 value (a.u.) of fluorecent intensity (FI) from 3000 cells	Average value of FI	Mean value of Average FI	Standard Deviatoin of Average FI	Relative FI (DIT1t-- Mean value as a standard)	Mean value of relative FI	SD of relative FI
1	DIT1t	−	0.282035	1.914410202	1.871742625	0.053100284	1.022795643	1.00	0.03
	DIT1t	−	0.254056	1.794965063			0.958980706		
	DIT1t	−	0.278249	1.897793697			1.013918085		
	DIT1t	−	0.274112	1.879801535			1.004305566		
2	m8	−	0.0864767	1.220328348	1.191304074	0.030107048	0.651974439	0.64	0.02
	m8	−	0.0809495	1.204895826			0.643729437		
	m8	−	0.0754726	1.189796262			0.635662321		
	m8	−	0.0607718	1.15019586			0.614505352		
3	m8	NAB6	0.173127	1.489796673	1.493260489	0.017299207	0.795940987	0.80	0.01
	m8	NAB6	0.17442	1.494238768			0.798314228		
	m8	NAB6	0.168352	1.47350631			0.787237674		
	m8	NAB6	0.180556	1.515500207			0.809673396		
4	m8	PAP1	0.176512	1.501453892	1.454441475	0.059982599	0.802168991	0.78	0.03
	m8	PAP1	0.14896	1.409159004			0.752859387		
	m8	PAP1	0.145019	1.396429452			0.746058477		
	m8	PAP1	0.179185	1.510723552			0.807121413		
5	m8	NAB6+PAP1	0.246784	1.765159685	1.719735458	0.076671924	0.94305684	0.92	0.04
	m8	NAB6+PAP1	0.234835	1.717255832			0.917463656		
	m8	NAB6+PAP1	0.207552	1.612694111			0.861600356		
	m8	NAB6+PAP1	0.251354	1.783832204			0.953032848		
6	m9	−	0.0856805	1.218093147	1.209520087	0.031937357	0.650780258	0.65	0.02
	m9	−	0.0737026	1.184957025			0.633076904		
	m9	−	0.0733277	1.183934565			0.632530643		
	m9	−	0.0972905	1.251095609			0.668412202		
7	m9	NAB6	0.232772	1.709117811	1.684162947	0.018741063	0.913115825	0.90	0.01
	m9	NAB6	0.221377	1.664857244			0.889469108		
	m9	NAB6	0.226841	1.685935673			0.900730501		
	m9	NAB6	0.224466	1.676741062			0.895818175		
8	m9	PAP1	0.232749	1.709027299	1.652764818	0.047146251	0.913067468	0.88	0.03
	m9	PAP1	0.214453	1.638524728			0.87540066		
	m9	PAP1	0.203302	1.596989277			0.853209868		
	m9	PAP1	0.22181	1.666517966			0.890356369		
9	m9	NAB6+PAP1	0.341348	2.194562733	2.145578855	0.111402749	1.172470352	1.15	0.06
	m9	NAB6+PAP1	0.349128	2.234230623			1.193663377		
	m9	NAB6+PAP1	0.336521	2.170306145			1.159510991		
	m9	NAB6+PAP1	0.29737	1.98321592			1.059555889		
10	m10	−	0.0615638	1.152295325	1.151434444	0.050222982	0.615627015	0.62	0.03
	m10	−	0.0400535	1.096613278			0.585878242		
	m10	−	0.085582	1.21781691			0.650632674		
	m10	−	0.0565284	1.139012263			0.608530387		
11	m10	NAB6	0.191266	1.553338119	1.606966381	0.036523513	0.829888735	0.86	0.02
	m10	NAB6	0.20799	1.614321385			0.862469746		
	m10	NAB6	0.212422	1.630879974			0.871316362		
	m10	NAB6	0.212008	1.629326046			0.870486158		
12	m10	PAP1	0.206147	1.607485263	1.606766086	0.066011532	0.858817469	0.86	0.04
	m10	PAP1	0.182064	1.520771622			0.81248971		
	m10	PAP1	0.225658	1.681349504			0.898280288		
	m10	PAP1	0.208833	1.617457954			0.864145494		
13	m10	NAB6+PAP1	0.260666	1.82249355	1.792044157	0.033493994	0.973688116	0.96	0.02
	m10	NAB6+PAP1	0.259729	1.818565718			0.971589627		
	m10	NAB6+PAP1	0.24823	1.771046647			0.946202017		
	m10	NAB6+PAP1	0.244542	1.756070713			0.938200952		
14	m11	−	0.0857189	1.218200855	1.187416729	0.038772382	0.650837802	0.63	0.02
	m11	−	0.0754654	1.189776537			0.635651783		
	m11	−	0.0826498	1.209622343			0.646254633		
	m11	−	0.0538722	1.13206718			0.604819896		
15	m11	NAB6	0.221924	1.666955476	1.620602487	0.031666111	0.890590113	0.87	0.02
	m11	NAB6	0.203408	1.597379108			0.853418139		
	m11	NAB6	0.205177	1.603898939			0.856901434		
	m11	NAB6	0.207951	1.614176424			0.862392299		
16	m11	PAP1	0.210738	1.624568395	1.614822026	0.02661294	0.867944328	0.86	0.01
	m11	PAP1	0.204477	1.601315841			0.855521384		
	m11	PAP1	0.200404	1.586368216			0.847535444		
	m11	PAP1	0.216703	1.647035652			0.879947718		
17	m11	NAB6+PAP1	0.34309	2.203383029	2.133732684	0.066524478	1.177182696	1.14	0.04
	m11	NAB6+PAP1	0.316149	2.070851705			1.106376314		
	m11	NAB6+PAP1	0.32776	2.126963318			1.136354587		

Population	Genetic Background	Overexpression	Average log10 value (a.u.) of fluorecent intensity (FI) from 3000 cells	Average value of FI	Mean value of Average FI	Standard Deviatoin of Average FI	Relative FI (DIT1t−−Mean value as a standard)	Mean value of relative FI	SD of relative FI
1	DIT1t	–	0.257869	1.810793805	1.820494746	0.028981158	0.994671261	1.00	0.02
	DIT1t	–	0.268932	1.857513591			1.020334497		
	DIT1t	–	0.261335	1.825303136			1.002641255		
	DIT1t	–	0.252457	1.788368452			0.982352987		
2	m12	–	0.0744888	1.187104086	1.157197442	0.040823643	0.652077733	0.64	0.02
	m12	–	0.0431982	1.104582606			0.606748582		
	m12	–	0.0761962	1.191780294			0.654646379		
	m12	–	0.0589279	1.145322783			0.629127212		
3	m12	NAB6	0.131885	1.354830609	1.371588586	0.057853963	0.744210118	0.75	0.03
	m12	NAB6	0.163298	1.456458116			0.800034232		
	m12	NAB6	0.129832	1.348441158			0.740700385		
	m12	NAB6	0.122748	1.326624459			0.728716445		
4	m12	PAP1	0.180753	1.516187808	1.493104322	0.045974013	0.832843825	0.82	0.03
	m12	PAP1	0.153613	1.4243378			0.782390503		
	m12	PAP1	0.181892	1.520169448			0.835030945		
	m12	PAP1	0.179472	1.511722232			0.830390879		
5	m12	NAB6+PAP1	0.306096	2.023466413	1.926145444	0.083655494	1.111492586	1.06	0.05
	m12	NAB6+PAP1	0.283842	1.922392219			1.055972407		
	m12	NAB6+PAP1	0.259987	1.819646389			0.999533997		
	m12	NAB6+PAP1	0.287595	1.939076754			1.065137243		
6	m13	–	0.103121	1.268005099	1.244498745	0.038746738	0.696516758	0.68	0.02
	m13	–	0.0943847	1.242752656			0.68264556		
	m13	–	0.106088	1.276697477			0.701291492		
	m13	–	0.0757439	1.190539749			0.653964947		
7	m13	NAB6	0.205749	1.606012791	1.655776123	0.084197702	0.882184799	0.91	0.05
	m13	NAB6	0.206099	1.607307607			0.882896042		
	m13	NAB6	0.25069	1.781106957			0.978364239		
	m13	NAB6	0.211835	1.628677137			0.894634352		
8	m13	PAP1	0.247476	1.767974513	1.675417838	0.112431328	0.971150572	0.92	0.06
	m13	PAP1	0.249721	1.777137372			0.976183742		
	m13	PAP1	0.200457	1.586561824			0.871500359		
	m13	PAP1	0.195899	1.569997642			0.862401633		
9	m13	NAB6+PAP1	0.343478	2.205352417	2.198311097	0.046643363	1.211402792	1.21	0.03
	m13	NAB6+PAP1	0.329153	2.133796508			1.172097043		
	m13	NAB6+PAP1	0.351267	2.245261865			1.2333251		
	m13	NAB6+PAP1	0.344163	2.2088336			1.213315009		
10	m14	–	0.0262864	1.062395934	1.095142109	0.024241976	0.583575392	0.60	0.01
	m14	–	0.0393338	1.094797508			0.601373616		
	m14	–	0.0426657	1.103229078			0.606005087		
	m14	–	0.0492746	1.120145917			0.615297528		
11	m14	NAB6	0.180484	1.515248979	1.455833975	0.079014872	0.832328125	0.80	0.04
	m14	NAB6	0.158175	1.439378462			0.790652357		
	m14	NAB6	0.130549	1.350669211			0.741924256		
	m14	NAB6	0.181283	1.518039248			0.833860823		
12	m14	PAP1	0.158376	1.440044789	1.468300992	0.048413346	0.791018371	0.81	0.03
	m14	PAP1	0.152057	1.419243781			0.779592352		
	m14	PAP1	0.18396	1.527425371			0.839016632		
	m14	PAP1	0.172162	1.486490028			0.816530798		
13	m14	NAB6+PAP1	0.272797	1.874118294	1.696426418	0.231101685	1.029455481	0.93	0.13
	m14	NAB6+PAP1	0.133276	1.359176947			0.746597567		
	m14	NAB6+PAP1	0.257497	1.809243412			0.993819629		
	m14	NAB6+PAP1	0.241339	1.743167017			0.957523784		
14	m15	–	0.0687263	1.17145686	1.145315444	0.036701169	0.643482692	0.63	0.02
	m15	–	0.0422447	1.102160139			0.605417918		
	m15	–	0.0718111	1.179807357			0.64806963		
	m15	–	0.0522465	1.127837421			0.61952248		
15	m15	NAB6	0.177187	1.503789333	1.571527491	0.07693162	0.826033328	0.86	0.04
	m15	NAB6	0.225863	1.682143338			0.924003402		
	m15	NAB6	0.189674	1.547654447			0.850128489		
	m15	NAB6	0.191038	1.552522847			0.852802707		
16	m15	PAP1	0.244196	1.754672218	1.808664783	0.065428623	0.963843605	0.99	0.04
	m15	PAP1	0.243328	1.751168756			0.961919149		
	m15	PAP1	0.273851	1.878672162			1.031956926		
	m15	PAP1	0.267206	1.850145995			1.016287468		
17	m15	NAB6+PAP1	0.357731	2.278930076	2.258279776	0.026887033	1.25181909	1.24	0.01
	m15	NAB6+PAP1	0.35618	2.270805827			1.24735643		
	m15	NAB6+PAP1	0.346146	2.218942253			1.218867705		
	m15	NAB6+PAP1	0.354961	2.26444095			1.243860195		

Population	Genetic Background	Overexpression	Average log10 value (a.u.) of fluorecent intensity (FI) from 3000 cells	Average value of FI	Mean value of Average FI	Standard Deviatoin of Average FI	Relative FI (DIT1t-- Mean value as a standard)	Mean value of relative FI	SD of relative FI
1	DIT1t	-	0.284047	1.92329986	1.91540776	0.049359415	1.004120324	1.00	0.03
	DIT1t	-	0.265686	1.843681934			0.962553234		
	DIT1t	-	0.290706	1.953016894			1.019635053		
	DIT1t	-	0.288167	1.941632353			1.013691389		
2	m16	-	0.0835585	1.212155957	1.213941904	0.00794276	0.63284486	0.63	0.00
	m16	-	0.0806102	1.203954848			0.628563209		
	m16	-	0.0873364	1.22274642			0.638373951		
	m16	-	0.0852586	1.216910392			0.635327066		
3	m16	NAB6	0.27663	1.890732111	1.853092269	0.046193409	0.987117287	0.97	0.02
	m16	NAB6	0.269202	1.858668762			0.970377588		
	m16	NAB6	0.25204	1.786652124			0.932778994		
	m16	NAB6	0.273306	1.876316078			0.979590935		
4	m16	PAP1	0.239646	1.736384898	1.665050267	0.05934788	0.906535378	0.87	0.03
	m16	PAP1	0.225954	1.682495843			0.87840087		
	m16	PAP1	0.202988	1.595835052			0.833156827		
	m16	PAP1	0.216294	1.645485274			0.859078316		
5	m16	NAB6+PAP1	0.359562	2.288558403	2.249381055	0.117463131	1.194815251	1.17	0.06
	m16	NAB6+PAP1	0.364109	2.312645149			1.207390508		
	m16	NAB6+PAP1	0.316903	2.074450136			1.083033169		
	m16	NAB6+PAP1	0.365838	2.321870533			1.212206916		
6	m17	-	0.093401	1.239940941	1.202014083	0.036518356	0.647350902	0.63	0.02
	m17	-	0.0886679	1.226500981			0.64033414		
	m17	-	0.0694993	1.173543791			0.612686142		
	m17	-	0.0674691	1.168070619			0.609828697		
7	m17	NAB6	0.229719	1.697145199	1.720272973	0.028440282	0.886049036	0.90	0.01
	m17	NAB6	0.232887	1.70957044			0.892536031		
	m17	NAB6	0.233667	1.712643616			0.894140481		
	m17	NAB6	0.24594	1.761732637			0.919768978		
8	m17	PAP1	0.219983	1.659521946	1.609390549	0.041446774	0.866406611	0.84	0.02
	m17	PAP1	0.193454	1.56118367			0.815065963		
	m17	PAP1	0.203002	1.595886497			0.833183685		
	m17	PAP1	0.209775	1.620970085			0.846279376		
9	m17	NAB6+PAP1	0.353614	2.257428481	2.208354381	0.056074456	1.178562877	1.15	0.03
	m17	NAB6+PAP1	0.342735	2.20158268			1.149406787		
	m17	NAB6+PAP1	0.350705	2.242358256			1.170694984		
	m17	NAB6+PAP1	0.328797	2.132048108			1.113104036		
10	m18	-	0.162178	1.452706902	1.458994838	0.025565782	0.75843219	0.76	0.01
	m18	-	0.154871	1.428469592			0.745778326		
	m18	-	0.173154	1.489889296			0.77784445		
	m18	-	0.165812	1.464913563			0.764805068		
11	m18	NAB6	0.410356	2.572503652	2.537382929	0.073406729	1.343057967	1.32	0.04
	m18	NAB6	0.397828	2.499355309			1.304868531		
	m18	NAB6	0.418457	2.620939524			1.368345466		
	m18	NAB6	0.390358	2.456733229			1.282616308		
12	m18	PAP1	0.444108	2.780404611	2.879854266	0.079586357	1.451599324	1.50	0.04
	m18	PAP1	0.462757	2.902398226			1.515290001		
	m18	PAP1	0.457151	2.86517399			1.495855895		
	m18	PAP1	0.472967	2.971440237			1.551335595		
13	m18	NAB6+PAP1	0.600003	3.981099206	3.730696354	0.382907955	2.078460414	1.95	0.20
	m18	NAB6+PAP1	0.499683	3.159970294			1.649763753		
	m18	NAB6+PAP1	0.589172	3.883041213			2.027266096		
	m18	NAB6+PAP1	0.590917	3.898674702			2.03542806		
14	m19	-	0.180987	1.517004957	1.484350649	0.064611418	0.79200105	0.77	0.03
	m19	-	0.168505	1.474025511			0.769562253		
	m19	-	0.145719	1.398682046			0.730226782		
	m19	-	0.189684	1.547690084			0.808021204		
15	m19	NAB6	0.366926	2.327694605	2.28207118	0.063323376	1.215247559	1.19	0.03
	m19	NAB6	0.341758	2.19663551			1.146823959		
	m19	NAB6	0.356315	2.271511815			1.185915533		
	m19	NAB6	0.367811	2.332442789			1.217726501		
16	m19	PAP1	0.460091	2.884635873	2.752803349	0.117760889	1.506016595	1.44	0.06
	m19	PAP1	0.424017	2.654709476			1.385976151		
	m19	PAP1	0.450202	2.819694129			1.472111676		
	m19	PAP1	0.423602	2.652173919			1.384652383		
17	m19	NAB6+PAP1	0.585534	3.850649594	3.623143864	0.229869519	2.010355014	1.89	0.12
	m19	NAB6+PAP1	0.564287	3.66679812			1.91436946		
	m19	NAB6+PAP1	0.564912	3.672078865			1.917126443		
	m19	NAB6+PAP1	0.518915	3.303048875			1.724462511		

Population	Genetic Background	Overexpression	Average log10 value (a.u.) of fluorecent intensity (FI) from 3000 cells	Average value of FI	Mean value of Average FI	Standard Deviatoin of Average FI	Relative FI (DIT1t-- Mean value as a standard)	Mean value of relative FI	SD of relative FI
1	DIT1t	–	0.296059	1.977238235	1.972461771	0.04614697	1.002421575	1.00	0.02
	DIT1t	–	0.287714	1.939608149			0.983343848		
	DIT1t	–	0.308769	2.035958869			1.032191801		
	DIT1t	–	0.287139	1.937041833			0.982042776		
2	m20	–	0.337803	2.176722167	2.191927637	0.078138359	1.103556073	1.11	0.04
	m20	–	0.348501	2.231007345			1.131077609		
	m20	–	0.356063	2.270194149			1.150944562		
	m20	–	0.320102	2.089786888			1.059481567		
3	m20	NAB6	0.487159	3.070145797	2.97653633	0.158473041	1.556504588	1.51	0.08
	m20	NAB6	0.495535	3.129932704			1.586815395		
	m20	NAB6	0.467197	2.932223027			1.486580409		
	m20	NAB6	0.443082	2.77384379			1.406285197		
4	m20	PAP1	0.586213	3.856674621	3.781690344	0.092769044	1.955259502	1.92	0.05
	m20	PAP1	0.585719	3.852290237			1.953036704		
	m20	PAP1	0.563584	3.660867412			1.855989031		
	m20	PAP1	0.574833	3.756929107			1.904690454		
5	m20	NAB6+PAP1	0.686387	4.857211337	4.807218641	0.314061198	2.462512282	2.44	0.16
	m20	NAB6+PAP1	0.716638	5.207604577			2.640154883		
	m20	NAB6+PAP1	0.648963	4.45618282			2.259198574		
	m20	NAB6+PAP1	0.672825	4.707875832			2.386802066		
6	m21	–	0.294712	1.971115168	1.897520855	0.050094558	0.999317298	0.96	0.03
	m21	–	0.275661	1.886518203			0.956428272		
	m21	–	0.270027	1.862202906			0.944100886		
	m21	–	0.271899	1.870247143			0.948179159		
7	m21	NAB6	0.502532	3.180768047	3.010336316	0.114458209	1.612587931	1.53	0.06
	m21	NAB6	0.471093	2.958645964			1.499976328		
	m21	NAB6	0.472361	2.96729688			1.504362175		
	m21	NAB6	0.467554	2.934634373			1.487802915		
8	m21	PAP1	0.647001	4.436096654	4.334441748	0.214190797	2.249015275	2.20	0.11
	m21	PAP1	0.636982	4.334929112			2.197725287		
	m21	PAP1	0.65614	4.530436004			2.296843503		
	m21	PAP1	0.605984	4.036305224			2.046328746		
9	m21	NAB6+PAP1	0.745592	5.56662544	5.487948389	0.187583881	2.822171522	2.78	0.10
	m21	NAB6+PAP1	0.747698	5.593684922			2.835890157		
	m21	NAB6+PAP1	0.716594	5.207077001			2.639887412		
	m21	NAB6+PAP1	0.746977	5.584406192			2.83118602		
10	m22	–	0.311471	2.048665246	2.014129499	0.042534281	1.038633689	1.02	0.02
	m22	–	0.309238	2.038158714			1.03330708		
	m22	–	0.304493	2.016011476			1.022078859		
	m22	–	0.290854	1.953682561			0.990479303		
11	m22	NAB6	0.490357	3.09283677	3.211819175	0.083516398	1.568008473	1.63	0.04
	m22	NAB6	0.514563	3.270114806			1.657885011		
	m22	NAB6	0.507116	3.214519022			1.629699023		
	m22	NAB6	0.514522	3.269806102			1.657728504		
12	m22	PAP1	0.670812	4.686104833	4.621008958	0.089350402	2.375764591	2.34	0.05
	m22	PAP1	0.663255	4.605268968			2.334782369		
	m22	PAP1	0.653291	4.500813319			2.281825374		
	m22	PAP1	0.671344	4.691848713			2.378676627		
13	m22	NAB6+PAP1	0.772666	5.924695027	5.888974258	0.128529741	3.003705883	2.99	0.07
	m22	NAB6+PAP1	0.761712	5.777128133			2.92889232		
	m22	NAB6+PAP1	0.76334	5.798824966			2.939892195		
	m22	NAB6+PAP1	0.782132	6.055248908			3.069894178		
14	m23	–	0.243024	1.749943391	1.719009265	0.057457638	0.887187482	0.87	0.03
	m23	–	0.235185	1.718640335			0.871317437		
	m23	–	0.247689	1.76884183			0.896768625		
	m23	–	0.214476	1.638611506			0.830744367		
15	m23	NAB6	0.449886	2.817643218	2.793058135	0.117059194	1.42849066	1.42	0.06
	m23	NAB6	0.418672	2.622237356			1.329423664		
	m23	NAB6	0.459988	2.883951815			1.462107838		
	m23	NAB6	0.454601	2.84840015			1.44408383		
16	m23	PAP1	0.611576	4.088612951	3.974077498	0.092590293	2.072847753	2.01	0.05
	m23	PAP1	0.592953	3.916994845			1.98584069		
	m23	PAP1	0.602859	4.007365916			2.031657076		
	m23	PAP1	0.589205	3.883336278			1.968776447		
17	m23	NAB6+PAP1	0.67977	4.783766795	4.826345532	0.119591925	2.425277318	2.45	0.06
	m23	NAB6+PAP1	0.698641	4.996213632			2.532983759		
	m23	NAB6+PAP1	0.673686	4.717218575			2.391538657		
	m23	NAB6+PAP1	0.681981	4.808183126			2.437655926		

Population	Genetic Background	Overexpression	Average log10 value (a.u.) of fluorecent intensity (FI) from 3000 cells	Average value of FI	Mean value of Average FI	Standard Deviatoin of Average FI	Relative FI (DIT1t–– Mean value as a standard)	Mean value of relative FI	SD of relative FI
1	DIT1t	–	0.310582	2.044475921	2.001666453	0.029853822	1.021386914	1.00	0.01
	DIT1t	–	0.300777	1.998835251			0.998585578		
	DIT1t	–	0.297935	1.985797685			0.992072222		
	DIT1t	–	0.296129	1.977556953			0.987955286		
2	m24	–	0.223309	1.672280016	1.626920645	0.045256907	0.835443894	0.81	0.02
	m24	–	0.213	1.633051948			0.815846189		
	m24	–	0.214335	1.638079593			0.818357919		
	m24	–	0.194312	1.564271022			0.781484357		
3	m24	NAB6	0.518564	3.300380405	3.22984618	0.098412653	1.648816365	1.61	0.05
	m24	NAB6	0.490264	3.09217454			1.544800102		
	m24	NAB6	0.508599	3.225514511			1.611414582		
	m24	NAB6	0.518687	3.301315265			1.649283406		
4	m24	PAP1	0.589538	3.88631501	3.859493251	0.02709489	1.941539763	1.93	0.01
	m24	PAP1	0.58838	3.875966368			1.93636975		
	m24	PAP1	0.58276	3.82613245			1.911473535		
	m24	PAP1	0.585411	3.849559176			1.923177146		
5	m24	NAB6+PAP1	0.70489	5.068623117	4.971608507	0.079049109	2.532201662	2.48	0.04
	m24	NAB6+PAP1	0.695767	4.9632597			2.479563812		
	m24	NAB6+PAP1	0.697135	4.978918301			2.487386595		
	m24	NAB6+PAP1	0.688031	4.875632912			2.435786894		
6	m25	–	0.264709	1.839539001	1.808104851	0.085841295	0.919003762	0.90	0.04
	m25	–	0.229116	1.694790418			0.846689725		
	m25	–	0.255132	1.799417749			0.898959838		
	m25	–	0.27845	1.898672237			0.948545765		
7	m25	NAB6	0.48828	3.078080687	3.045685147	0.043161732	1.537759042	1.52	0.02
	m25	NAB6	0.489321	3.085467669			1.541449458		
	m25	NAB6	0.47659	2.996332466			1.496918961		
	m25	NAB6	0.480418	3.022859764			1.510171568		
8	m25	PAP1	0.636904	4.334150622	4.196705785	0.111114387	2.16527115	2.10	0.06
	m25	PAP1	0.616803	4.138119233			2.067337057		
	m25	PAP1	0.626695	4.233455509			2.11496551		
	m25	PAP1	0.610777	4.081097778			2.038850065		
9	m25	NAB6+PAP1	0.736955	5.457013146	5.453932023	0.10230568	2.726235002	2.72	0.05
	m25	NAB6+PAP1	0.747651	5.593079597			2.794211588		
	m25	NAB6+PAP1	0.733498	5.413747548			2.704620213		
	m25	NAB6+PAP1	0.728507	5.351887799			2.673716089		
10	m26	–	0.0796832	1.201387752	1.164079445	0.036654878	0.600193779	0.58	0.02
	m26	–	0.0706676	1.176705003			0.587862679		
	m26	–	0.0659303	1.163939214			0.581485098		
	m26	–	0.0469966	1.11428581			0.556679065		
11	m26	NAB6	0.183149	1.524575723	1.5329462	0.011501723	0.761653232	0.77	0.01
	m26	NAB6	0.182421	1.522022245			0.760377556		
	m26	NAB6	0.187331	1.5393274			0.76902293		
	m26	NAB6	0.18917	1.545859432			0.772286227		
12	m26	PAP1	0.18805	1.541877958	1.515336706	0.03133526	0.770297147	0.76	0.02
	m26	PAP1	0.167403	1.470289988			0.734532962		
	m26	PAP1	0.184438	1.529107435			0.763917202		
	m26	PAP1	0.181864	1.520071442			0.759402967		
13	m26	NAB6+PAP1	0.249785	1.77739928	1.769729425	0.016857502	0.887959769	0.88	0.01
	m26	NAB6+PAP1	0.250049	1.778480059			0.888499708		
	m26	NAB6+PAP1	0.250074	1.778582439			0.888550856		
	m26	NAB6+PAP1	0.24166	1.74445592			0.871501802		
14	m27	–	0.292133	1.959444649	1.92789987	0.031772055	0.978906674	0.96	0.02
	m27	–	0.279244	1.902146664			0.950281533		
	m27	–	0.290261	1.951016759			0.974696237		
	m27	–	0.278523	1.898991409			0.948705218		
15	m27	NAB6	0.457601	2.868144317	2.982577901	0.076423003	1.432878247	1.49	0.04
	m27	NAB6	0.480815	3.025624303			1.511552686		
	m27	NAB6	0.479246	3.014713183			1.506101668		
	m27	NAB6	0.48027	3.021829802			1.509657015		
16	m27	PAP1	0.60517	4.028747045	3.916480131	0.171189384	2.012696491	1.96	0.09
	m27	PAP1	0.610224	4.075904502			2.036255589		
	m27	PAP1	0.568207	3.700044949			1.84848227		
	m27	PAP1	0.586725	3.861224027			1.929004716		
17	m27	NAB6+PAP1	0.735274	5.435931808	5.024895574	0.734684183	2.715703109	2.51	0.37
	m27	NAB6+PAP1	0.732338	5.399306729			2.697405815		
	m27	NAB6+PAP1	0.727531	5.339873883			2.667714132		
	m27	NAB6+PAP1	0.593781	3.924469878			1.960601314		

Population	Genetic Background	Overexpression	Average log10 value (a.u.) of fluorecent intensity (FI) from 3000 cells	Average value of FI	Mean value of Average FI	Standard Deviatoin of Average FI	Relative FI (DIT1t-- Mean value as a standard)	Mean value of relative FI	SD of relative FI
1	DIT1t	–	0.232674	1.708732186	1.752882269	0.035214897	0.974812865	1.00	0.02
	DIT1t	–	0.240861	1.741249481			0.993363623		
	DIT1t	–	0.252147	1.787092368			1.019516484		
	DIT1t	–	0.249065	1.77445504			1.012307028		
2	m28	–	0.183158	1.524607318	1.518935473	0.034542712	0.869771658	0.87	0.02
	m28	–	0.174168	1.493371985			0.851952246		
	m28	–	0.19468	1.56559707			0.89315586		
	m28	–	0.173817	1.492165519			0.85126397		
3	m28	NAB6	0.385783	2.430989035	2.41808136	0.020019832	1.386852431	1.38	0.01
	m28	NAB6	0.38727	2.439326872			1.391609074		
	m28	NAB6	0.380254	2.400236303			1.369308336		
	m28	NAB6	0.380532	2.401773231			1.370185136		
4	m28	PAP1	0.55431	3.583521381	3.57037122	0.102209379	2.044359421	2.04	0.06
	m28	PAP1	0.565735	3.679044154			2.09885411		
	m28	PAP1	0.554697	3.586716081			2.046181963		
	m28	PAP1	0.535573	3.432203264			1.958034105		
5	m28	NAB6+PAP1	0.615106	4.121981137	4.271372587	0.140456134	2.351544773	2.44	0.08
	m28	NAB6+PAP1	0.645423	4.420007436			2.52156549		
	m28	NAB6+PAP1	0.621779	4.185805073			2.387955625		
	m28	NAB6+PAP1	0.639257	4.357696704			2.486017904		
6	m29	–	0.107634	1.281250359	1.279977992	0.025232936	0.730939197	0.73	0.01
	m29	–	0.109014	1.285328093			0.7332655		
	m29	–	0.116324	1.307145704			0.745712206		
	m29	–	0.0955835	1.246187814			0.710936402		
7	m29	NAB6	0.290582	1.952459347	1.9442805	0.042774863	1.113856522	1.11	0.02
	m29	NAB6	0.274919	1.883297804			1.074400624		
	m29	NAB6	0.297348	1.983115459			1.131345496		
	m29	NAB6	0.291868	1.95824939			1.117159678		
8	m29	PAP1	0.433464	2.713088747	2.694912958	0.05788153	1.547787205	1.54	0.03
	m29	PAP1	0.431261	2.699361193			1.539955787		
	m29	PAP1	0.439699	2.752320469			1.570168469		
	m29	PAP1	0.417452	2.614881425			1.491761011		
9	m29	NAB6+PAP1	0.521513	3.322867316	3.429170456	0.08042714	1.895659153	1.96	0.05
	m29	NAB6+PAP1	0.546275	3.517831229			2.006883915		
	m29	NAB6+PAP1	0.535462	3.43132615			1.957533721		
	m29	NAB6+PAP1	0.537146	3.444657129			1.965138897		
10	m30	–	0.130856	1.351624328	1.310919206	0.050018031	0.771086771	0.75	0.03
	m30	–	0.110777	1.290556433			0.73624821		
	m30	–	0.09678	1.249625848			0.712897763		
	m30	–	0.130935	1.351870216			0.771227047		
11	m30	NAB6	0.358233	2.281565809	2.227551667	0.107461304	1.301608128	1.27	0.06
	m30	NAB6	0.317037	2.075090299			1.183816127		
	m30	NAB6	0.34911	2.234138023			1.2745511		
	m30	NAB6	0.365378	2.319412535			1.323199268		
12	m30	PAP1	0.39237	2.468141191	2.494703476	0.031398884	1.408047326	1.42	0.02
	m30	PAP1	0.397708	2.498664807			1.425460712		
	m30	PAP1	0.393493	2.474531576			1.41169297		
	m30	PAP1	0.404402	2.537476331			1.447602259		
13	m30	NAB6+PAP1	0.541254	3.477394797	3.336281098	0.401251537	1.983815376	1.90	0.23
	m30	NAB6+PAP1	0.438579	2.745231667			1.566124386		
	m30	NAB6+PAP1	0.541839	3.482082047			1.9864894		
	m30	NAB6+PAP1	0.561151	3.640415879			2.076817105		

	Control_1	Control_2	Control_3	Control_AV	Control_SD	nab6Δ_1	nab6Δ_2	nab6Δ_3	nab6Δ_AV	nab6Δ_SD
0	0.003	0.111	0	0.038	0.063	0.002	-0.016	-0.006	-0.007	0.009
0.5	-0.004	-0.018	-0.009	-0.010	0.007	-0.009	-0.020	-0.017	-0.015	0.006
1	-0.009	-0.024	-0.009	-0.014	0.009	-0.010	-0.018	-0.018	-0.015	0.005
1.5	-0.006	-0.023	-0.012	-0.014	0.009	-0.006	-0.011	-0.016	-0.011	0.005
2	-0.003	-0.019	-0.009	-0.010	0.008	-0.004	-0.003	-0.011	-0.006	0.004
2.5	-0.002	-0.018	-0.008	-0.009	0.008	0.000	0.002	-0.009	-0.002	0.006
3	0.01	-0.006	-0.001	0.001	0.008	0.005	0.013	0.001	0.006	0.006
3.5	0.013	0.001	0.009	0.008	0.006	0.019	0.035	0.005	0.020	0.015
4	0.026	0.013	0.022	0.020	0.007	0.033	0.054	0.019	0.035	0.018
4.5	0.045	0.033	0.033	0.037	0.007	0.049	0.076	0.037	0.054	0.020
5	0.066	0.061	0.049	0.059	0.009	0.071	0.109	0.057	0.079	0.027
5.5	0.093	0.081	0.068	0.081	0.013	0.095	0.144	0.080	0.106	0.033
6	0.122	0.114	0.098	0.111	0.012	0.135	0.190	0.114	0.146	0.039
6.5	0.164	0.162	0.127	0.151	0.021	0.176	0.238	0.146	0.187	0.047
7	0.216	0.211	0.163	0.197	0.029	0.218	0.291	0.185	0.231	0.054
7.5	0.264	0.26	0.215	0.246	0.027	0.264	0.360	0.243	0.289	0.062
8	0.343	0.33	0.26	0.311	0.045	0.329	0.441	0.298	0.356	0.075
8.5	0.41	0.404	0.32	0.378	0.050	0.396	0.545	0.365	0.435	0.096
9	0.507	0.488	0.391	0.462	0.062	0.478	0.655	0.466	0.533	0.106
9.5	0.606	0.603	0.478	0.562	0.073	0.575	0.780	0.547	0.634	0.127
10	0.73	0.707	0.578	0.672	0.082	0.684	0.905	0.670	0.753	0.132
10.5	0.863	0.829	0.672	0.788	0.102	0.800	1.033	0.782	0.872	0.140
11	0.983	0.953	0.793	0.910	0.102	0.930	1.161	0.905	0.999	0.141
11.5	1.097	1.059	0.877	1.011	0.118	1.050	1.265	1.035	1.117	0.129
12	1.215	1.16	0.993	1.123	0.116	1.180	1.384	1.151	1.238	0.127
12.5	1.311	1.253	1.092	1.219	0.113	1.289	1.450	1.267	1.335	0.100
13	1.417	1.341	1.195	1.318	0.113	1.399	1.502	1.369	1.423	0.070
13.5	1.505	1.424	1.284	1.404	0.112	1.491	1.574	1.456	1.507	0.061
14	1.545	1.491	1.36	1.465	0.095	1.563	1.630	1.509	1.567	0.061
14.5	1.604	1.534	1.431	1.523	0.087	1.628	1.688	1.579	1.632	0.055
15	1.678	1.596	1.492	1.589	0.093	1.667	1.732	1.621	1.673	0.056
15.5	1.697	1.622	1.522	1.614	0.088	1.717	1.751	1.666	1.711	0.043
16	1.726	1.662	1.559	1.649	0.084	1.755	1.795	1.716	1.755	0.040
16.5	1.774	1.708	1.595	1.692	0.091	1.790	1.817	1.773	1.793	0.022
17	1.792	1.747	1.632	1.724	0.083	1.842	1.863	1.789	1.831	0.038
17.5	1.818	1.775	1.672	1.755	0.075	1.856	1.901	1.821	1.859	0.040
18	1.833	1.799	1.682	1.771	0.079	1.901	1.925	1.865	1.897	0.030
18.5	1.889	1.822	1.729	1.813	0.080	1.918	1.963	1.890	1.924	0.037
19	1.903	1.863	1.741	1.836	0.084	1.964	2.000	1.940	1.968	0.030
19.5	1.928	1.89	1.777	1.865	0.079	1.993	2.007	1.962	1.987	0.023
20	1.95	1.904	1.824	1.893	0.064	2.016	2.035	1.985	2.012	0.025
20.5	1.963	1.93	1.84	1.911	0.064	2.049	2.073	2.020	2.047	0.027
21	2.024	1.97	1.867	1.954	0.080	2.072	2.087	2.049	2.069	0.019
21.5	2.037	1.979	1.907	1.974	0.065	2.110	2.115	2.061	2.095	0.030
22	2.056	2.036	1.927	2.006	0.069	2.131	2.141	2.102	2.125	0.020
22.5	2.079	2.028	1.954	2.020	0.063	2.160	2.152	2.129	2.147	0.016
23	2.102	2.049	1.964	2.038	0.070	2.169	2.188	2.162	2.173	0.013
23.5	2.137	2.072	1.987	2.065	0.075	2.201	2.185	2.156	2.181	0.023
24	2.156	2.092	2.013	2.087	0.072	2.218	2.206	2.191	2.205	0.014

	ov_NAB6_1	ov_NAB6_2	ov_NAB6_3	ov_NAB6_AV	ov_NAB6_SD	ov_PAP1_1	ov_PAP1_2	ov_PAP1_3	ov_PAP1_AV	ov_PAP1_SD	ov_NAB6 +PAP1_1	ov_NAB6 +PAP1_2	ov_NAB6 +PAP1_3	ov_NAB6 +PAP1_AV	ov_NAB6 +PAP1_SD
0	0.000	-0.006	-0.001	(0.002)	0.003	0.000	-0.005	-0.001	(0.002)	0.003	0.000	0.005	-0.516	(0.170)	0.299
0.5	-0.006	-0.010	-0.004	(0.007)	0.003	-0.004	-0.010	-0.008	(0.007)	0.003	0.001	0.002	0.000	0.001	0.001
1	0.000	-0.011	-0.011	(0.007)	0.006	-0.009	-0.015	-0.015	(0.013)	0.003	0.004	0.009	0.008	0.007	0.003
1.5	-0.012	-0.016	-0.013	(0.014)	0.002	-0.008	-0.012	-0.017	(0.012)	0.005	0.009	0.011	0.004	0.008	0.004
2	-0.011	-0.013	-0.012	(0.012)	0.001	-0.003	-0.005	-0.015	(0.008)	0.006	0.013	0.011	0.006	0.010	0.004
2.5	-0.010	-0.006	-0.009	(0.008)	0.002	-0.003	-0.002	-0.012	(0.006)	0.006	0.020	0.020	0.013	0.018	0.004
3	-0.002	-0.004	-0.003	(0.003)	0.001	0.001	0.005	-0.003	0.001	0.004	0.028	0.027	0.018	0.024	0.006
3.5	0.004	0.003	0.008	0.005	0.003	0.012	0.011	0.002	0.008	0.006	0.034	0.032	0.026	0.031	0.004
4	0.006	0.011	0.013	0.010	0.004	0.024	0.026	0.018	0.023	0.004	0.040	0.038	0.034	0.037	0.003
4.5	0.024	0.023	0.023	0.023	0.001	0.030	0.041	0.035	0.035	0.006	0.049	0.047	0.045	0.047	0.002
5	0.032	0.033	0.034	0.033	0.001	0.044	0.057	0.048	0.050	0.007	0.059	0.059	0.054	0.057	0.003
5.5	0.047	0.052	0.053	0.051	0.003	0.059	0.073	0.069	0.067	0.007	0.074	0.060	0.060	0.065	0.008
6	0.070	0.067	0.078	0.072	0.006	0.071	0.100	0.092	0.088	0.015	0.077	0.069	0.071	0.072	0.004
6.5	0.089	0.093	0.092	0.091	0.002	0.099	0.124	0.119	0.114	0.013	0.096	0.082	0.080	0.086	0.009
7	0.118	0.111	0.118	0.116	0.004	0.127	0.155	0.151	0.144	0.015	0.106	0.091	0.081	0.093	0.013
7.5	0.142	0.137	0.144	0.141	0.004	0.154	0.195	0.192	0.180	0.023	0.122	0.104	0.097	0.108	0.013
8	0.168	0.172	0.178	0.173	0.005	0.184	0.228	0.224	0.212	0.024	0.144	0.119	0.112	0.125	0.017
8.5	0.211	0.209	0.213	0.211	0.002	0.222	0.267	0.275	0.255	0.029	0.156	0.129	0.122	0.136	0.018
9	0.249	0.247	0.256	0.251	0.005	0.271	0.329	0.310	0.303	0.030	0.177	0.143	0.136	0.152	0.022
9.5	0.300	0.293	0.299	0.297	0.004	0.306	0.371	0.365	0.347	0.036	0.195	0.169	0.153	0.172	0.021
10	0.335	0.339	0.361	0.345	0.014	0.373	0.437	0.410	0.407	0.032	0.222	0.187	0.163	0.191	0.030
10.5	0.391	0.413	0.415	0.406	0.013	0.439	0.503	0.466	0.469	0.032	0.247	0.209	0.180	0.212	0.034
11	0.453	0.456	0.484	0.464	0.017	0.489	0.574	0.525	0.529	0.043	0.272	0.230	0.199	0.234	0.037
11.5	0.503	0.536	0.551	0.530	0.025	0.546	0.647	0.584	0.592	0.051	0.307	0.257	0.221	0.262	0.043
12	0.560	0.590	0.636	0.595	0.038	0.616	0.730	0.663	0.670	0.057	0.336	0.282	0.238	0.285	0.049
12.5	0.615	0.657	0.702	0.658	0.044	0.705	0.801	0.723	0.743	0.051	0.374	0.314	0.260	0.316	0.057
13	0.684	0.747	0.778	0.736	0.048	0.772	0.883	0.790	0.815	0.060	0.390	0.340	0.287	0.339	0.052
13.5	0.751	0.811	0.862	0.808	0.056	0.850	0.961	0.865	0.892	0.060	0.440	0.380	0.311	0.377	0.065
14	0.812	0.879	0.937	0.876	0.063	0.918	1.027	0.932	0.959	0.059	0.461	0.409	0.346	0.405	0.058
14.5	0.874	0.954	1.046	0.958	0.086	1.000	1.121	1.000	1.040	0.070	0.492	0.448	0.364	0.435	0.065
15	0.946	1.029	1.127	1.034	0.091	1.066	1.175	1.078	1.106	0.060	0.535	0.499	0.400	0.478	0.070
15.5	1.013	1.121	1.182	1.105	0.086	1.151	1.254	1.141	1.182	0.063	0.575	0.518	0.433	0.509	0.071
16	1.087	1.177	1.255	1.173	0.084	1.226	1.325	1.204	1.252	0.064	0.613	0.570	0.462	0.548	0.078
16.5	1.129	1.219	1.340	1.229	0.106	1.274	1.391	1.284	1.316	0.065	0.658	0.616	0.494	0.589	0.085
17	1.178	1.275	1.383	1.279	0.103	1.354	1.437	1.341	1.377	0.052	0.707	0.650	0.541	0.633	0.084
17.5	1.241	1.332	1.446	1.340	0.103	1.393	1.500	1.413	1.435	0.057	0.750	0.702	0.592	0.681	0.081
18	1.291	1.400	1.502	1.398	0.106	1.448	1.575	1.469	1.497	0.068	0.783	0.735	0.622	0.713	0.083
18.5	1.333	1.465	1.575	1.458	0.121	1.508	1.620	1.551	1.560	0.057	0.824	0.776	0.658	0.753	0.085
19	1.381	1.512	1.646	1.513	0.133	1.566	1.686	1.616	1.623	0.060	0.887	0.822	0.702	0.804	0.094
19.5	1.420	1.557	1.702	1.560	0.141	1.624	1.734	1.668	1.675	0.055	0.906	0.861	0.761	0.843	0.074
20	1.475	1.616	1.768	1.620	0.147	1.687	1.784	1.719	1.730	0.049	0.942	0.897	0.788	0.876	0.079
20.5	1.530	1.661	1.817	1.669	0.144	1.734	1.827	1.762	1.774	0.048	0.990	0.941	0.823	0.918	0.086
21	1.587	1.709	1.869	1.722	0.141	1.783	1.863	1.803	1.816	0.042	1.022	0.974	0.869	0.955	0.078
21.5	1.616	1.740	1.906	1.754	0.146	1.834	1.918	1.858	1.870	0.043	1.059	1.017	0.937	1.004	0.062
22	1.676	1.803	1.955	1.811	0.140	1.879	1.942	1.893	1.905	0.033	1.092	1.056	0.956	1.035	0.070
22.5	1.723	1.836	1.998	1.852	0.138	1.921	1.977	1.928	1.942	0.031	1.130	1.098	1.015	1.081	0.059
23	1.754	1.848	2.045	1.882	0.149	1.966	2.013	1.971	1.983	0.026	1.175	1.128	1.044	1.116	0.066
23.5	1.798	1.874	2.058	1.910	0.134	1.993	2.054	2.010	2.019	0.031	1.197	1.159	1.090	1.149	0.054
24	1.831	1.903	2.101	1.945	0.140	2.034	2.069	2.035	2.046	0.020	1.224	1.200	1.127	1.184	0.051

Population	Genetic Background	Overexpression	Average log10 value (a.u.) of fluorecent intensity (FI) from 3000 cells	Average value of FI	Mean value of Average FI	Standard Deviatoin of Average FI	Relative FI (DIT1t-- Mean value as a standard)	Mean value of relative FI	SD of relative FI
1	DIT1t	–	0.256748	1.806125815	1.824958715	0.02444383	0.989680369	1.00	0.01
	DIT1t	–	0.257426	1.808947655			0.991226618		
	DIT1t	–	0.261366	1.825433431			1.000260124		
	DIT1t	–	0.269356	1.859327959			1.018832889		
2	DIT1t	NAB6	0.471387	2.960649528	2.946937643	0.083190944	1.622310413	1.61	0.05
	DIT1t	NAB6	0.477915	3.005488012			1.646880002		
	DIT1t	NAB6	0.47655	2.996056506			1.641711937		
	DIT1t	NAB6	0.451104	2.825556527			1.548285177		
3	DIT1t	PAP1	0.629821	4.264037349	4.152092724	0.18440626	2.336511677	2.28	0.10
	DIT1t	PAP1	0.608156	4.056542216			2.222813142		
	DIT1t	PAP1	0.595895	3.943619451			2.160936255		
	DIT1t	PAP1	0.637907	4.344171879			2.380422003		
4	DIT1t	NAB6+PAP1	0.704635	5.065647902	5.208113898	0.173008247	2.775760273	2.85	0.09
	DIT1t	NAB6+PAP1	0.714107	5.177343739			2.836964856		
	DIT1t	NAB6+PAP1	0.737062	5.458357792			2.990948643		
	DIT1t	NAB6+PAP1	0.710211	5.131106159			2.811628623		
5	DIT1t-d7	–	0.305467	2.020537895	2.072520533	0.048722828	1.107169098	1.14	0.03
	DIT1t-d7	–	0.328542	2.130796624			1.167586207		
	DIT1t-d7	–	0.311112	2.046972461			1.12165412		
	DIT1t-d7	–	0.320515	2.091775153			1.146204095		
6	DIT1t-d7	NAB6	0.452379	2.833863973	2.936273176	0.080693688	1.552837305	1.61	0.04
	DIT1t-d7	NAB6	0.481281	3.028872555			1.65969374		
	DIT1t-d7	NAB6	0.466366	2.926617735			1.603662434		
	DIT1t-d7	NAB6	0.470666	2.955738442			1.619619347		
7	DIT1t-d7	PAP1	0.674956	4.731033247	4.747777333	0.099306155	2.592405629	2.60	0.05
	DIT1t-d7	PAP1	0.673418	4.714308511			2.583241184		
	DIT1t-d7	PAP1	0.668093	4.65685805			2.551760767		
	DIT1t-d7	PAP1	0.689212	4.888909523			2.678915135		
8	DIT1t-d7	NAB6+PAP1	0.757721	5.724281728	5.410778299	0.567223007	3.136663685	2.96	0.31
	DIT1t-d7	NAB6+PAP1	0.660983	4.581239536			2.510325027		
	DIT1t-d7	NAB6+PAP1	0.741758	5.517698933			3.023465072		
	DIT1t-d7	NAB6+PAP1	0.764915	5.819892998			3.189054607		
9	DIT1t-d21	–	0.269272	1.858968368	1.952453876	0.069706914	1.018635848	1.07	0.04
	DIT1t-d21	–	0.287682	1.939465238			1.06274472		
	DIT1t-d21	–	0.302377	2.006212814			1.099319561		
	DIT1t-d21	–	0.302151	2.005169084			1.098747642		
10	DIT1t-d21	NAB6	0.494399	3.121756323	3.081039797	0.139262222	1.710590106	1.69	0.08
	DIT1t-d21	NAB6	0.466268	2.925957408			1.603300603		
	DIT1t-d21	NAB6	0.504526	3.195405659			1.75094682		
11	DIT1t-d21	PAP1	0.513764	3.264104093	4.230597956	0.681430546	1.788590649	2.32	0.15
	DIT1t-d21	PAP1	0.682986	4.819322617			2.640784461		
	DIT1t-d21	PAP1	0.631072	4.27633776			2.343251781		
	DIT1t-d21	PAP1	0.659215	4.562627356			2.500126341		
12	DIT1t-d21	NAB6+PAP1	0.675654	4.738643098	5.2832804	0.441026781	2.596575505	2.90	0.24
	DIT1t-d21	NAB6+PAP1	0.708802	5.114486069			2.802521518		
	DIT1t-d21	NAB6+PAP1	0.754666	5.68415615			3.11467657		
	DIT1t-d21	NAB6+PAP1	0.747865	5.595836285			3.066281028		

	1	5	significant
Mean	1	1.13565338	
Variance	0.000179404	0.000712786	
Observations	4	4	
Pearson Correlation	0.000446095		
Hypothesized Mean Difference	0		
df	6		
t Stat	-9.083056619		
P(T<=t) one-tail	4.99779E-05		
t Critical one-tail	3.142668403		
P(T<=t) two-tail	9.99559E-05		
t Critical two-tail	3.707428021		

	1	9	significant
Mean	1	1.069861943	
Variance	0.000179404	0.001458968	
Observations	4	4	
Pearson Correlation	0.000819186		
Hypothesized Mean Difference	0		
df	6		
t Stat	-3.451949576		
P(T<=t) one-tail	0.006800255		
t Critical one-tail	3.142668403		
P(T<=t) two-tail	0.01360051		
t Critical two-tail	3.707428021		

	2	6	no difference
Mean	1.614796882	1.608953206	
Variance	0.002078002	0.001955118	
Observations	4	4	
Pearson Correlation	0.00201656		
Hypothesized Mean Difference	0		
df	6		
t Stat	0.184032915		
P(T<=t) one-tail	0.430024914		
t Critical one-tail	3.142668403		
P(T<=t) two-tail	0.860049828		
t Critical two-tail	3.707428021		

	2	10	no difference
Mean	1.614796882	1.688279176	
Variance	0.002078002	0.005823185	
Observations	4	3	
Pearson Correlation	0.003576075		
Hypothesized Mean Difference	0		
df	5		
t Stat	-1.608870136		
P(T<=t) one-tail	0.084278973		
t Critical one-tail	3.364929999		
P(T<=t) two-tail	0.168557946		
t Critical two-tail	4.032142984		

	3	7	significant
Mean	2.275170769	2.601580679	
Variance	0.010210458	0.002961053	
Observations	4	4	
Pearson Correlation	0.006585756		
Hypothesized Mean Difference	0		
df	6		
t Stat	-5.68820868		
P(T<=t) one-tail	0.000636898		
t Critical one-tail	3.142668403		
P(T<=t) two-tail	0.001273796		
t Critical two-tail	3.707428021		

	3	11	no difference
Mean	2.275170769	2.318188308	
Variance	0.010210458	0.139423861	
Observations	4	4	
Pearson Correlation	0.07481716		
Hypothesized Mean Difference	0		
df	6		
t Stat	-0.222412888		
P(T<=t) one-tail	0.41568507		
t Critical one-tail	3.142668403		
P(T<=t) two-tail	0.83137014		
t Critical two-tail	3.707428021		

	4	8	no difference
Mean	2.853825599	2.964877098	
Variance	0.008987265	0.096605441	
Observations	4	4	
Pearson Correlation	0.052796353		
Hypothesized Mean Difference	0		
df	6		
t Stat	-0.683498336		
P(T<=t) one-tail	0.259899903		
t Critical one-tail	3.142668403		
P(T<=t) two-tail	0.519799806		
t Critical two-tail	3.707428021		

	4	12	no difference
Mean	2.853825599	2.895013655	
Variance	0.008987265	0.058401478	
Observations	4	4	
Pearson Correlation	0.033694371		
Hypothesized Mean Difference	0		
df	6		
t Stat	-0.317327433		
P(T<=t) one-tail	0.380873685		
t Critical one-tail	3.142668403		
P(T<=t) two-tail	0.761747371		
t Critical two-tail	3.707428021		

Population	Genetic Background	Overexpression	Average log10 value (a.u.) of fluorecent intensity (FI) from 3000 cells	Average value of FI	Mean value of Average FI	Standard Deviatoin of Average FI	Relative FI (DIT1t-- Mean value as a standard)	Mean value of relative FI	SD of relative FI
1	DIT1t	-	0.240695	1.740584052	1.732573757	0.007409583	1.00462335	1.00	0.00
	DIT1t	-	0.236196	1.722645841			0.994269845		
	DIT1t	-	0.238866	1.733269121			1.000401348		
	DIT1t	-	0.238998	1.733796013			1.000705457		
2	DIT1t	NAB6	0.471387	2.960649528	2.946937643	0.083190944	1.708815868	1.70	0.05
	DIT1t	NAB6	0.477915	3.005488012			1.734695565		
	DIT1t	NAB6	0.47655	2.996056506			1.729251926		
	DIT1t	NAB6	0.451104	2.825556527			1.630843429		
3	DIT1t	PAP1	0.629821	4.264037349	4.152092724	0.18440626	2.461100043	2.40	0.11
	DIT1t	PAP1	0.608156	4.056542216			2.341338832		
	DIT1t	PAP1	0.595895	3.943619451			2.276162521		
	DIT1t	PAP1	0.637907	4.344171879			2.507351772		
4	DIT1t	NAB6+PAP1	0.704635	5.065647902	5.208113898	0.173008247	2.923770421	3.01	0.10
	DIT1t	NAB6+PAP1	0.714107	5.177343739			2.988238577		
	DIT1t	NAB6+PAP1	0.737062	5.458357792			3.150433146		
	DIT1t	NAB6+PAP1	0.710211	5.131106159			2.961551356		
5	DIT1t-m22	-	0.265561	1.843151356	1.814292706	0.027460426	1.063822737	1.05	0.02
	DIT1t-m22	-	0.255669	1.801644084			1.039865736		
	DIT1t-m22	-	0.262461	1.83004176			1.056256192		
	DIT1t-m22	-	0.250989	1.782333623			1.028720201		
6	DIT1t-m22	NAB6	0.451796	2.830062326	2.73942229	0.157620705	1.633444068	1.58	0.09
	DIT1t-m22	NAB6	0.414917	2.599662682			1.500462922		
	DIT1t-m22	NAB6	0.417143	2.613021602			1.50817337		
	DIT1t-m22	NAB6	0.46463	2.914942552			1.682434898		
7	DIT1t-m22	PAP1	0.670767	4.685619301	4.656727863	0.033360959	2.704427031	2.69	0.02
	DIT1t-m22	PAP1	0.665378	4.627836425			2.671076141		
	DIT1t-m22	PAP1	0.670767	4.685619301			2.704427031		
	DIT1t-m22	PAP1	0.665378	4.627836425			2.671076141		
8	DIT1t-m22	NAB6+PAP1	0.822768	6.649178624	6.569293175	0.235494893	3.837746357	3.79	0.14
	DIT1t-m22	NAB6+PAP1	0.823015	6.652961343			3.839929652		
	DIT1t-m22	NAB6+PAP1	0.794021	6.223303768			3.591941609		
	DIT1t-m22	NAB6+PAP1	0.829415	6.751728964			3.896935953		
9	DIT1t-d22	-	0.276089	1.888378295	1.885302315	0.037340756	1.089926641	1.09	0.02
	DIT1t-d22	-	0.286223	1.932960592			1.115658473		
	DIT1t-d22	-	0.265383	1.842396076			1.063386807		
	DIT1t-d22	-	0.273574	1.877474296			1.083633115		
10	DIT1t-d22	NAB6	0.44486	2.785223174	2.658360291	0.09914	1.607563986	1.53	0.06
	DIT1t-d22	NAB6	0.411186	2.577424782			1.48762774		
	DIT1t-d22	NAB6	0.41189	2.581606227			1.49004117		
	DIT1t-d22	NAB6	0.429621	2.689186981			1.552134199		
11	DIT1t-d22	PAP1	0.676717	4.750255834	4.810043946	0.061987772	2.741733687	2.78	0.04
	DIT1t-d22	PAP1	0.686029	4.853209064			2.801155821		
	DIT1t-d22	PAP1	0.687788	4.872905621			2.812524201		
	DIT1t-d22	PAP1	0.677954	4.763805264			2.749554093		
12	DIT1t-d22	NAB6+PAP1	0.831184	6.779286686	6.567414279	0.165649979	3.912841609	3.79	0.10
	DIT1t-d22	NAB6+PAP1	0.812389	6.492156793			3.747117124		
	DIT1t-d22	NAB6+PAP1	0.805718	6.393195719			3.689999167		
	DIT1t-d22	NAB6+PAP1	0.819874	6.605017916			3.812257856		

	1	5	significant
Mean	1	1.047166216	
Variance	1.82896E-05	0.000251207	
Observations	4	4	
Pearson Correlation	0.000134748		
Hypothesized Mean Difference	0		
df	6		
t Stat	-5.746252464		
P(T<=t) one-tail	0.000604255		
t Critical one-tail	3.142668403		
P(T<=t) two-tail	0.001208511		
t Critical two-tail	3.707428021		

	1	9	significant
Mean	1	1.088151259	
Variance	1.82896E-05	0.000464497	
Observations	4	4	
Pearson Correlation	0.000241393		
Hypothesized Mean Difference	0		
df	6		
t Stat	-8.023816514		
P(T<=t) one-tail	0.000100068		
t Critical one-tail	3.142668403		
P(T<=t) two-tail	0.000200135		
t Critical two-tail	3.707428021		

	2	6	no difference
Mean	1.700901697	1.581128814	
Variance	0.002305519	0.00827643	
Observations	4	4	
Pearson Correlation	0.005290975		
Hypothesized Mean Difference	0		
df	6		
t Stat	2.328657743		
P(T<=t) one-tail	0.029374274		
t Critical one-tail	3.142668403		
P(T<=t) two-tail	0.058748549		

t Critical two-tail	3.707428021	

	2	10	significant
Mean	1.700901697	1.534341774	
Variance	0.002305519	0.003274269	
Observations	4	4	
Pearson Correlation	0.002789894		
Hypothesized Mean Difference	0		
df	6		
t Stat	4.459556484		
P(T<=t) one-tail	0.002142523		
t Critical one-tail	3.142668403		
P(T<=t) two-tail	0.004285047		
t Critical two-tail	3.707428021		

	3	7	significant
Mean	2.396488292	2.687751586	
Variance	0.011328381	0.000370761	
Observations	4	4	
Pearson Correlation	0.005849571		
Hypothesized Mean Difference	0		
df	6		
t Stat	-5.385657725		
P(T<=t) one-tail	0.000843299		
t Critical one-tail	3.142668403		
P(T<=t) two-tail	0.001686599		
t Critical two-tail	3.707428021		

	3	11	significant
Mean	2.396488292	2.77624195	
Variance	0.011328381	0.001280055	
Observations	4	4	
Pearson Correlation	0.006304218		
Hypothesized Mean Difference	0		
df	6		
t Stat	-6.763964987		
P(T<=t) one-tail	0.000254896		
t Critical one-tail	3.142668403		
P(T<=t) two-tail	0.000509791		
t Critical two-tail	3.707428021		

	4	8	significant
Mean	3.005998375	3.791638393	
Variance	0.009971263	0.018474791	
Observations	4	4	
Pearson Correlation	0.014223027		
Hypothesized Mean Difference	0		
df	6		
t Stat	-9.316281107		
P(T<=t) one-tail	4.33061E-05		
t Critical one-tail	3.142668403		
P(T<=t) two-tail	8.66122E-05		
t Critical two-tail	3.707428021		

	4	12	significant
Mean	3.005998375	3.790553939	
Variance	0.009971263	0.009141118	
Observations	4	4	
Pearson Correlation	0.00955619		
Hypothesized Mean Difference	0		
df	6		
t Stat	-11.35001181		
P(T<=t) one-tail	1.40078E-05		
t Critical one-tail	3.142668403		
P(T<=t) two-tail	2.80155E-05		
t Critical two-tail	3.707428021		

W303-1A / TDH3pro::GFP

Population	Time after reinoculation at OD600=0.1	Terminator	Average log10 value (a.u.) of fluorecent intensity (FI) from 3000 cells	Average value of FI	Mean value of Average FI	Standard Deviatoin of Average FI
1	6h	PGK1t	-0.119456	0.759528369	0.753375405	0.008633064
	6h	PGK1t	-0.124764	0.75030182		
	6h	PGK1t	-0.11856	0.761096982		
	6h	PGK1t	-0.12926	0.742574446		
2	6h	wild-type DIT1t	0.241034	1.741943241	1.787952092	0.034035957
	6h	wild-type DIT1t	0.260634	1.822359269		
	6h	wild-type DIT1t	0.251985	1.786425873		
	6h	wild-type DIT1t	0.255533	1.801079985		
3	6h	DIT1t-m22	0.289435	1.947309578	1.946740352	0.01234284
	6h	DIT1t-m22	0.286787	1.935472477		
	6h	DIT1t-m22	0.293089	1.96376267		
	6h	DIT1t-m22	0.287895	1.940416684		
4	6h	DIT1t-d7	0.317415	2.076897196	2.025282462	0.034718387
	6h	DIT1t-d7	0.304155	2.014443078		
	6h	DIT1t-d7	0.301817	2.003627575		
	6h	DIT1t-d7	0.302366	2.006162		
5	6h	DIT1t-d21	0.281554	1.912291083	1.928262975	0.012417506
	6h	DIT1t-d21	0.288138	1.941502705		
	6h	DIT1t-d21	0.284624	1.925856838		
	6h	DIT1t-d21	0.286322	1.933401272		
6	6h	DIT1t-d22	0.320376	2.091105768	2.094419029	0.053151642
	6h	DIT1t-d22	0.305816	2.022162256		
	6h	DIT1t-d22	0.325983	2.118278216		
	6h	DIT1t-d22	0.331656	2.146129874		
7	12h	PGK1t	-0.0370237	0.918282483	0.921945105	0.007103499
	12h	PGK1t	-0.0339291	0.924849146		
	12h	PGK1t	-0.0313475	0.930363149		
	12h	PGK1t	-0.0389181	0.914285643		
8	12h	wild-type DIT1t	0.442879	2.77254753	2.749204539	0.056574938
	12h	wild-type DIT1t	0.449833	2.817299382		
	12h	wild-type DIT1t	0.43368	2.71443846		
	12h	wild-type DIT1t	0.430161	2.692532784		
9	12h	DIT1t-m22	0.496244	3.135046595	3.079137321	0.05157521
	12h	DIT1t-m22	0.486517	3.065610677		
	12h	DIT1t-m22	0.491555	3.101380129		
	12h	DIT1t-m22	0.479217	3.014511882		
10	12h	DIT1t-d7	0.494946	3.125690696	3.04743976	0.069596189
	12h	DIT1t-d7	0.487813	3.074772584		
	12h	DIT1t-d7	0.481005	3.026948277		
	12h	DIT1t-d7	0.471636	2.962347484		
11	12h	DIT1t-d21	0.460584	2.887912297	2.880693283	0.039502402
	12h	DIT1t-d21	0.46725	2.932580889		
	12h	DIT1t-d21	0.453531	2.841390997		
	12h	DIT1t-d21	0.456501	2.860888948		
12	12h	DIT1t-d22	0.494211	3.120405251	3.207480469	0.14751809
	12h	DIT1t-d22	0.484515	3.051511423		
	12h	DIT1t-d22	0.527994	3.372826489		
	12h	DIT1t-d22	0.516559	3.285178713		
13	24h	PGK1t	-0.0768213	0.837873973	0.841493252	0.013368222
	24h	PGK1t	-0.083718	0.824673426		
	24h	PGK1t	-0.0717269	0.847760349		
	24h	PGK1t	-0.0676961	0.85566526		
	24h	wild-type DIT1t	0.386592	2.435521681	2.518769775	0.110271982

14	24h	wild-type DIT1t	0.427234	2.674447026		
	24h	wild-type DIT1t	0.388434	2.445873547		
	24h	wild-type DIT1t	0.401269	2.519236848		
15	24h	DIT1t-m22	0.54378	3.497679406	3.393934845	0.080047969
	24h	DIT1t-m22	0.531788	3.402420607		
	24h	DIT1t-m22	0.527649	3.370148207		
	24h	DIT1t-m22	0.519236	3.30549116		
16	24h	DIT1t-d7	0.446931	2.798536657	2.863119246	0.080983958
	24h	DIT1t-d7	0.469588	2.948410845		
	24h	DIT1t-d7	0.464798	2.916070369		
	24h	DIT1t-d7	0.44552	2.789459113		
17	24h	DIT1t-d21	0.509367	3.23122351	3.069758735	0.108604572
	24h	DIT1t-d21	0.478215	3.007564847		
	24h	DIT1t-d21	0.477703	3.004021247		
	24h	DIT1t-d21	0.482334	3.036225336		
18	24h	DIT1t-d22	0.565108	3.673736473	3.597837756	0.139045819
	24h	DIT1t-d22	0.536436	3.439030282		
	24h	DIT1t-d22	0.573786	3.747882786		
	24h	DIT1t-d22	0.547861	3.530701483		
19	30h	PGK1t	-0.122583	0.754079267	0.75505896	0.013632203
	30h	PGK1t	-0.128113	0.744538226		
	30h	PGK1t	-0.110929	0.77458842		
	30h	PGK1t	-0.126662	0.747029926		
20	30h	wild-type DIT1t	0.379883	2.398186755	2.401647628	0.032049585
	30h	wild-type DIT1t	0.373171	2.361407836		
	30h	wild-type DIT1t	0.381613	2.407758917		
	30h	wild-type DIT1t	0.387254	2.439237005		
21	30h	DIT1t-m22	0.550978	3.556133039	3.279995314	0.264553189
	30h	DIT1t-m22	0.469245	2.946083149		
	30h	DIT1t-m22	0.532785	3.410240439		
	30h	DIT1t-m22	0.50617	3.207524631		
22	30h	DIT1t-d7	0.401734	2.521935645	2.605920157	0.06251485
	30h	DIT1t-d7	0.42702	2.673129508		
	30h	DIT1t-d7	0.417782	2.616869106		
	30h	DIT1t-d7	0.416931	2.611746371		
23	30h	DIT1t-d21	0.457966	2.870555843	2.812988843	0.114499495
	30h	DIT1t-d21	0.42687	2.672206401		
	30h	DIT1t-d21	0.443232	2.774802007		
	30h	DIT1t-d21	0.467518	2.934391122		
24	30h	DIT1t-d22	0.57218	3.734048891	3.669809141	0.278881435
	30h	DIT1t-d22	0.52126	3.32093213		
	30h	DIT1t-d22	0.60153	3.995121577		
	30h	DIT1t-d22	0.559803	3.629133965		

	2	3	significant
Mean	1.787952092	1.946740352	
Variance	0.001158446	0.000152346	
Observations	4	4	
Pearson Correlation	0.000655396		
Hypothesized Mean Differer	0		
df	6		
t Stat	-8.77165385		
P(T<=t) one-tail	6.08275E-05		
t Critical one-tail	3.142668403		
P(T<=t) two-tail	0.000121655		
t Critical two-tail	3.707428021		

	2	4	significant
Mean	1.787952092	2.025282462	
Variance	0.001158446	0.001205366	

Observations	4	4
Pearson Correlation	0.001181906	
Hypothesized Mean Differer	0	
df	6	
t Stat	-9.762853574	
P(T<=t) one-tail	3.32019E-05	
t Critical one-tail	3.142668403	
P(T<=t) two-tail	6.64038E-05	
t Critical two-tail	3.707428021	

	2	5	significant
Mean	1.787952092	1.928262975	
Variance	0.001158446	0.000154194	
Observations	4	4	
Pearson Correlation	0.00065632		
Hypothesized Mean Differer	0		
df	6		
t Stat	-7.74548113		
P(T<=t) one-tail	0.00012167		
t Critical one-tail	3.142668403		
P(T<=t) two-tail	0.00024334		
t Critical two-tail	3.707428021		

	2	6	significant
Mean	1.787952092	2.094419029	
Variance	0.001158446	0.002825097	
Observations	4	4	
Pearson Correlation	0.001991772		
Hypothesized Mean Differer	0		
df	6		
t Stat	-9.711332962		
P(T<=t) one-tail	3.42162E-05		
t Critical one-tail	3.142668403		
P(T<=t) two-tail	6.84324E-05		
t Critical two-tail	3.707428021		

	8	9	significant
Mean	2.749204539	3.079137321	
Variance	0.003200724	0.002660002	
Observations	4	4	
Pearson Correlation	0.002930363		
Hypothesized Mean Differer	0		
df	6		
t Stat	-8.619454027		
P(T<=t) one-tail	6.71074E-05		
t Critical one-tail	3.142668403		
P(T<=t) two-tail	0.000134215		
t Critical two-tail	3.707428021		

	8	10	significant
Mean	2.749204539	3.04743976	
Variance	0.003200724	0.00484363	
Observations	4	4	
Pearson Correlation	0.004022177		
Hypothesized Mean Differer	0		
df	6		
t Stat	-6.650332561		

P(T<=t) one-tail	0.000279222
t Critical one-tail	3.142668403
P(T<=t) two-tail	0.000558444
t Critical two-tail	3.707428021

	8	11	significant
Mean	2.749204539	2.880693283	
Variance	0.003200724	0.00156044	
Observations	4	4	
Pearson Correlation	0.002380582		
Hypothesized Mean Differer	0		
df	6		
t Stat	-3.811202545		
P(T<=t) one-tail	0.004425569		
t Critical one-tail	3.142668403		
P(T<=t) two-tail	0.008851138		
t Critical two-tail	3.707428021		

	8	12	significant
Mean	2.749204539	3.207480469	
Variance	0.003200724	0.021761587	
Observations	4	4	
Pearson Correlation	0.012481155		
Hypothesized Mean Differer	0		
df	6		
t Stat	-5.801157451		
P(T<=t) one-tail	0.000575125		
t Critical one-tail	3.142668403		
P(T<=t) two-tail	0.00115025		
t Critical two-tail	3.707428021		

	9	12	no difference
Mean	3.079137321	3.207480469	
Variance	0.002660002	0.021761587	
Observations	4	4	
Pearson Correlation	0.012210795		
Hypothesized Mean Differer	0		
df	6		
t Stat	-1.642539123		
P(T<=t) one-tail	0.075792559		
t Critical one-tail	3.142668403		
P(T<=t) two-tail	0.151585118		
t Critical two-tail	3.707428021		

	14	15	significant
Mean	2.518769775	3.393934845	
Variance	0.01215991	0.006407677	
Observations	4	4	
Pearson Correlation	0.009283794		
Hypothesized Mean Differer	0		
df	6		
t Stat	-12.84524035		
P(T<=t) one-tail	6.84042E-06		
t Critical one-tail	3.142668403		
P(T<=t) two-tail	1.36808E-05		
t Critical two-tail	3.707428021		

	14	16	significant
Mean	2.518769775	2.863119246	
Variance	0.01215991	0.006558401	
Observations	4	4	
Pearson Correlation	0.009359156		
Hypothesized Mean Differer	0		
df	6		
t Stat	-5.033801452		
P(T<=t) one-tail	0.001185681		
t Critical one-tail	3.142668403		
P(T<=t) two-tail	0.002371362		
t Critical two-tail	3.707428021		

	14	17	significant
Mean	2.518769775	3.069758735	
Variance	0.01215991	0.011794953	
Observations	4	4	
Pearson Correlation	0.011977432		
Hypothesized Mean Differer	0		
df	6		
t Stat	-7.119935281		
P(T<=t) one-tail	0.000193069		
t Critical one-tail	3.142668403		
P(T<=t) two-tail	0.000386138		
t Critical two-tail	3.707428021		

	14	18	significant
Mean	2.518769775	3.597837756	
Variance	0.01215991	0.01933374	
Observations	4	4	
Pearson Correlation	0.015746825		
Hypothesized Mean Differer	0		
df	6		
t Stat	-12.16094447		
P(T<=t) one-tail	9.39959E-06		
t Critical one-tail	3.142668403		
P(T<=t) two-tail	1.87992E-05		
t Critical two-tail	3.707428021		

	15	18	significant	p<0.05
Mean	3.393934845	3.597837756		
Variance	0.006407677	0.01933374		
Observations	4	4		
Pearson Correlation	0.012870709			
Hypothesized Mean Differer	0			
df	6			
t Stat	-2.541775546			
P(T<=t) one-tail	0.021987742			
t Critical one-tail	3.142668403			
P(T<=t) two-tail	0.043975485			
t Critical two-tail	3.707428021			

	21	24	significant	p<0.05
Mean	3.279995314	3.669809141		
Variance	0.06998839	0.077774855		
Observations	4	4		

Pearson Correlation	0.073881622
Hypothesized Mean Differer	0
df	6
t Stat	-2.028168463
P(T<=t) one-tail	0.044444493
t Critical one-tail	3.142668403
P(T<=t) two-tail	0.088888985
t Critical two-tail	3.707428021

ACT1pro::GFP

Population	Time after reinoculation at OD600=0.1	Terminator	Average log10 value (a.u.) of fluorecent intensity (FI) from 3000 cells	Average value of FI	Mean value of Average FI	Standard Deviatoin of Average FI
1	6h	PGK1t	0.618308	4.152484308	4.10938419	0.031828611
	6h	PGK1t	0.61218	4.094303192		
	6h	PGK1t	0.614067	4.112131554		
	6h	PGK1t	0.610513	4.078617704		
2	6h	wild-type DIT1t	1.03921	10.94485469	10.86178188	0.079470084
	6h	wild-type DIT1t	1.03559	10.85400457		
	6h	wild-type DIT1t	1.03288	10.78648639		
3	6h	DIT1t-d22	1.08317	12.11072102	11.93207403	0.182774301
	6h	DIT1t-d22	1.07903	11.99582164		
	6h	DIT1t-d22	1.0674	11.67884783		
	6h	DIT1t-d22	1.07711	11.94290561		
4	12h	PGK1t	0.778735	6.008070221	6.007603055	0.061684332
	12h	PGK1t	0.780277	6.029440309		
	12h	PGK1t	0.783157	6.06955708		
	12h	PGK1t	0.772567	5.923344612		
5	12h	wild-type DIT1t	1.33776	21.76506658	20.69649723	1.095069925
	12h	wild-type DIT1t	1.31697	20.74770192		
	12h	wild-type DIT1t	1.29174	19.57672319		
6	12h	DIT1t-d22	1.36007	22.91236927	21.67181448	1.199612624
	12h	DIT1t-d22	1.3476	22.2638363		
	12h	DIT1t-d22	1.32975	21.36731734		
	12h	DIT1t-d22	1.30414	20.14373503		
7	24h	PGK1t	0.687034	4.86445287	4.839062909	0.053861292
	24h	PGK1t	0.684748	4.838915074		
	24h	PGK1t	0.689189	4.888650615		
	24h	PGK1t	0.677993	4.764233077		
8	24h	wild-type DIT1t	1.31493	20.65047283	19.22788395	1.279764258
	24h	wild-type DIT1t	1.27561	18.86296678		
	24h	wild-type DIT1t	1.25936	18.17021223		
9	24h	DIT1t-d22	1.31382	20.59776031	20.07745689	0.516847644
	24h	DIT1t-d22	1.30793	20.3202946		
	24h	DIT1t-d22	1.30095	19.9963164		
	24h	DIT1t-d22	1.2877	19.39545624		
10	30h	PGK1t	0.630514	4.270846868	4.30475684	0.063761287
	30h	PGK1t	0.63562	4.321355546		
	30h	PGK1t	0.642097	4.386286549		
	30h	PGK1t	0.627421	4.240538397		
11	30h	wild-type DIT1t	1.27613	18.88556578	17.37169375	1.408700879
	30h	wild-type DIT1t	1.20681	16.09941146		
	30h	wild-type DIT1t	1.23376	17.13010401		
12	30h	DIT1t-d22	1.25062	17.808199	17.85805721	0.238696169
	30h	DIT1t-d22	1.25527	17.99989617		
	30h	DIT1t-d22	1.24412	17.54365184		
	30h	DIT1t-d22	1.25721	18.08048183		

	2	3	significant
Mean	10.86178188	11.93207403	
Variance	0.006315494	0.033406445	
Observations	3	4	
Pearson Correlation	0.022570065		
Hypothesized Mean Differer	0		
df	5		

t Stat	-9.327763764	
P(T<=t) one-tail	0.000119217	
t Critical one-tail	3.364929999	
P(T<=t) two-tail	0.000238435	
t Critical two-tail	4.032142984	

	5	6	no difference
Mean	20.69649723	21.67181448	
Variance	1.19917814	1.439070447	
Observations	3	4	
Pearson Correlation	1.343113524		
Hypothesized Mean Differer	0		
df	5		
t Stat	-1.101872001		
P(T<=t) one-tail	0.160354864		
t Critical one-tail	3.364929999		
P(T<=t) two-tail	0.320709727		
t Critical two-tail	4.032142984		

	8	9	no difference
Mean	19.22788395	20.07745689	
Variance	1.637796556	0.267131488	
Observations	3	4	
Pearson Correlation	0.815397515		
Hypothesized Mean Differer	0		
df	5		
t Stat	-1.231849303		
P(T<=t) one-tail	0.136387255		
t Critical one-tail	3.364929999		
P(T<=t) two-tail	0.272774509		
t Critical two-tail	4.032142984		

	11	12	no difference
Mean	17.37169375	17.85805721	
Variance	1.984438168	0.056975861	
Observations	3	4	
Pearson Correlation	0.827960784		
Hypothesized Mean Differer	0		
df	5		
t Stat	-0.699838224		
P(T<=t) one-tail	0.257620869		
t Critical one-tail	3.364929999		
P(T<=t) two-tail	0.515241737		
t Critical two-tail	4.032142984		

TDH3pro::mKO2

Population	Time after reinoculation at OD600=0.1	Terminator	Average log10 value (a.u.) of fluorecent intensity (FI) from 3000 cells	Average value of FI	Mean value of Average FI	Standard Deviatoin of Average FI
1	6h	PGK1t	0.472952	2.971337609	2.941615146	0.020464403
	6h	PGK1t	0.467752	2.935972612		
	6h	PGK1t	0.466054	2.924515988		
	6h	PGK1t	0.467554	2.934634373		
2	6h	wild-type DIT1t	0.829464	6.752490782	6.712674905	0.029379232
	6h	wild-type DIT1t	0.825595	6.692602017		
	6h	wild-type DIT1t	0.827179	6.717056479		
	6h	wild-type DIT1t	0.825332	6.688550339		
3	6h	DIT1t-d22	0.850156	7.082001265	7.161073637	0.093178827
	6h	DIT1t-d22	0.853651	7.139223859		
	6h	DIT1t-d22	0.863077	7.295868538		
	6h	DIT1t-d22	0.852919	7.127200887		
4	12h	PGK1t	0.925691	8.427349395	8.55859611	0.233108045
	12h	PGK1t	0.919884	8.315416368		
	12h	PGK1t	0.946295	8.836799473		
	12h	PGK1t	0.937258	8.654819203		
5	12h	wild-type DIT1t	1.2866	19.34639274	18.34480355	0.812664582
	12h	wild-type DIT1t	1.25355	17.92874953		
	12h	wild-type DIT1t	1.26986	18.61486967		
	12h	wild-type DIT1t	1.24277	17.48920225		
6	12h	DIT1t-d22	1.29675	19.80386695	19.33663533	0.410065987
	12h	DIT1t-d22	1.28429	19.24376302		
	12h	DIT1t-d22	1.2894	19.4715265		
	12h	DIT1t-d22	1.27479	18.82738485		
7	24h	PGK1t	1.18924	15.46108615	16.04325689	0.707958646
	24h	PGK1t	1.19198	15.55893978		
	24h	PGK1t	1.23049	17.00160806		
	24h	PGK1t	1.20821	16.15139357		
8	24h	wild-type DIT1t	1.69119	49.1122691	46.87162543	1.56001043
	24h	wild-type DIT1t	1.66922	46.68958352		
	24h	wild-type DIT1t	1.66366	46.09565601		
	24h	wild-type DIT1t	1.65886	45.58899307		
9	24h	DIT1t-d22	1.70553	50.76098023	49.09357805	1.290974891
	24h	DIT1t-d22	1.69213	49.21868429		
	24h	DIT1t-d22	1.68792	48.74386923		
	24h	DIT1t-d22	1.67807	47.65077846		
10	30h	PGK1t	1.08296	12.10486638	12.82407599	0.904378573
	30h	PGK1t	1.08094	12.0486947		
	30h	PGK1t	1.14307	13.90176683		
	30h	PGK1t	1.12192	13.24097605		
11	30h	wild-type DIT1t	1.64722	44.38334191	41.13742027	2.189206504
	30h	wild-type DIT1t	1.60658	40.41848213		
	30h	wild-type DIT1t	1.60348	40.13100168		
	30h	wild-type DIT1t	1.59788	39.61685537		
12	30h	DIT1t-d22	1.64243	43.89651071	42.44090164	0.988127155
	30h	DIT1t-d22	1.62401	42.07363161		
	30h	DIT1t-d22	1.62429	42.10076623		
	30h	DIT1t-d22	1.62006	41.69269801		

	2	3	significant
Mean	6.712674905	7.161073637	
Variance	0.000863139	0.008682294	

Observations	4	4
Pearson Correlation	0.004772717	
Hypothesized Mean Differer	0	
df	6	
t Stat	-9.179025033	
P(T<=t) one-tail	4.7098E-05	
t Critical one-tail	3.142668403	
P(T<=t) two-tail	9.41961E-05	
t Critical two-tail	3.707428021	

	5	6	significant	p<0.05
Mean	18.34480355	19.33663533		
Variance	0.660423722	0.168154113		
Observations	4	4		
Pearson Correlation	0.414288918			
Hypothesized Mean Differer	0			
df	6			
t Stat	-2.179221423			
P(T<=t) one-tail	0.036069361			
t Critical one-tail	3.142668403			
P(T<=t) two-tail	0.072138722			
t Critical two-tail	3.707428021			

	8	9	significant	p<0.05
Mean	46.87162543	49.09357805		
Variance	2.433632541	1.666616168		
Observations	4	4		
Pearson Correlation	2.050124354			
Hypothesized Mean Differer	0			
df	6			
t Stat	-2.194621808			
P(T<=t) one-tail	0.03531178			
t Critical one-tail	3.142668403			
P(T<=t) two-tail	0.07062356			
t Critical two-tail	3.707428021			

	11	12	no difference
Mean	41.13742027	42.44090164	
Variance	4.792625116	0.976395274	
Observations	4	4	
Pearson Correlation	2.884510195		
Hypothesized Mean Differer	0		
df	6		
t Stat	-1.085384926		
P(T<=t) one-tail	0.159711789		
t Critical one-tail	3.142668403		
P(T<=t) two-tail	0.319423577		
t Critical two-tail	3.707428021		

A451, TDH3pro::GFP

Population	Time after reinoculation at OD600=0.1	Terminator	Average log10 value (a.u.) of fluorecent intensity (FI) from 3000 cells	Average value of FI	Mean value of Average FI	Standard Deviatoin of Average FI
1	6h	PGK1t	-0.0318824	0.92921797	0.919078776	0.007019422
	6h	PGK1t	-0.0394256	0.913217867		
	6h	PGK1t	-0.0380712	0.916070294		
	6h	PGK1t	-0.0372477	0.917808975		
2	6h	wild-type DIT1t	0.321983	2.098857724	2.077941986	0.017784809
	6h	wild-type DIT1t	0.314971	2.065242245		
	6h	wild-type DIT1t	0.314132	2.061256319		
	6h	wild-type DIT1t	0.3194	2.086411655		
3	6h	DIT1t-d22	0.384394	2.423226446	2.396490998	0.025708641
	6h	DIT1t-d22	0.373244	2.361804795		
	6h	DIT1t-d22	0.381033	2.404545503		
	6h	DIT1t-d22	0.379557	2.396387249		
4	12h	PGK1t	0.0261231	1.061996536	1.052630067	0.0063449
	12h	PGK1t	0.0203483	1.047968672		
	12h	PGK1t	0.0214488	1.050627587		
	12h	PGK1t	0.0211593	1.049927473		
5	12h	wild-type DIT1t	0.441418	2.763236138	2.738112046	0.025766749
	12h	wild-type DIT1t	0.439379	2.750293231		
	12h	wild-type DIT1t	0.437041	2.735526964		
	12h	wild-type DIT1t	0.431909	2.703391849		
6	12h	DIT1t-d22	0.491567	3.101465825	3.019051401	0.075685095
	12h	DIT1t-d22	0.480373	3.022546563		
	12h	DIT1t-d22	0.482006	3.033933099		
	12h	DIT1t-d22	0.465124	2.918260118		
7	24h	PGK1t	0.0757147	1.190459705	1.197979525	0.016554943
	24h	PGK1t	0.0873551	1.222799071		
	24h	PGK1t	0.0753085	1.189346777		
	24h	PGK1t	0.075296	1.189312545		
8	24h	wild-type DIT1t	0.634404	4.309272918	4.180080658	0.129593571
	24h	wild-type DIT1t	0.629646	4.262319491		
	24h	wild-type DIT1t	0.61513	4.122208932		
	24h	wild-type DIT1t	0.60493	4.026521292		
9	24h	DIT1t-d22	0.66211	4.593143352	4.536010765	0.099534343
	24h	DIT1t-d22	0.655726	4.526119332		
	24h	DIT1t-d22	0.665087	4.624736571		
	24h	DIT1t-d22	0.643457	4.400043803		
10	30h	PGK1t	0.0500856	1.122239627	1.138620859	0.016865872
	30h	PGK1t	0.064848	1.161042186		
	30h	PGK1t	0.0530557	1.129940825		
	30h	PGK1t	0.0573849	1.141260799		
11	30h	wild-type DIT1t	0.637346	4.338563917	4.264918331	0.081143177
	30h	wild-type DIT1t	0.635489	4.320052255		
	30h	wild-type DIT1t	0.619283	4.161817193		
	30h	wild-type DIT1t	0.627288	4.239239957		
12	30h	DIT1t-d22	0.670189	4.679387386	4.556818029	0.12707534
	30h	DIT1t-d22	0.661153	4.583033165		
	30h	DIT1t-d22	0.661482	4.586506359		
	30h	DIT1t-d22	0.64131	4.378345205		

	2	3	significant
Mean	2.077941986	2.396490998	
Variance	0.000316299	0.000660934	

Observations	4	4	
Pearson Correlation	0.000488617		
Hypothesized Mean Differer	0		
df	6		
t Stat	-20.38013472		
P(T<=t) one-tail	4.53605E-07		
t Critical one-tail	3.142668403		
P(T<=t) two-tail	9.0721E-07		
t Critical two-tail	3.707428021		

	5	6	significant
Mean	2.738112046	3.019051401	
Variance	0.000663925	0.005728234	
Observations	4	4	
Pearson Correlation	0.00319608		
Hypothesized Mean Differer	0		
df	6		
t Stat	-7.027790283		
P(T<=t) one-tail	0.000207237		
t Critical one-tail	3.142668403		
P(T<=t) two-tail	0.000414473		
t Critical two-tail	3.707428021		

	8	9	significant
Mean	4.180080658	4.536010765	
Variance	0.016794494	0.009907085	
Observations	4	4	
Pearson Correlation	0.01335079		
Hypothesized Mean Differer	0		
df	6		
t Stat	-4.356384931		
P(T<=t) one-tail	0.002394623		
t Critical one-tail	3.142668403		
P(T<=t) two-tail	0.004789246		
t Critical two-tail	3.707428021		

	11	12	significant
Mean	4.264918331	4.556818029	
Variance	0.006584215	0.016148142	
Observations	4	4	
Pearson Correlation	0.011366179		
Hypothesized Mean Differer	0		
df	6		
t Stat	-3.872054001		
P(T<=t) one-tail	0.004123114		
t Critical one-tail	3.142668403		
P(T<=t) two-tail	0.008246228		
t Critical two-tail	3.707428021		

TDO2, TDH3pro::GFP

Population	Time after reinoculation at OD600=0.1	Terminator	Average log10 value (a.u.) of fluorecent intensity (FI) from 3000 cells	Average value of FI	Mean value of Average FI	Standard Deviatoin of Average FI
1	6h	PGK1t	0.479898	3.019242527	2.941692552	0.111005432
	6h	PGK1t	0.483296	3.042958294		
	6h	PGK1t	0.46252	2.900814783		
	6h	PGK1t	0.44774	2.803754606		
2	6h	wild-type DIT1t	0.739978	5.495130365	5.261716931	0.173723891
	6h	wild-type DIT1t	0.721969	5.27192229		
	6h	wild-type DIT1t	0.715664	5.195938475		
	6h	wild-type DIT1t	0.706195	5.083876595		
3	6h	DIT1t-d22	0.898721	7.919923743	7.69845226	0.193561066
	6h	DIT1t-d22	0.890226	7.766511685		
	6h	DIT1t-d22	0.883397	7.645343452		
	6h	DIT1t-d22	0.872857	7.462030159		
4	12h	PGK1t	0.512764	3.256596862	3.365526303	0.267937682
	12h	PGK1t	0.545054	3.50795489		
	12h	PGK1t	0.483811	3.046568869		
	12h	PGK1t	0.56241	3.650984591		
5	12h	wild-type DIT1t	0.819756	6.603223544	6.734067819	0.272438142
	12h	wild-type DIT1t	0.853262	7.132832079		
	12h	wild-type DIT1t	0.824325	6.673059543		
	12h	wild-type DIT1t	0.814724	6.527156112		
6	12h	DIT1t-d22	1.00989	10.23033841	9.920203597	0.252600642
	12h	DIT1t-d22	0.994823	9.881502845		
	12h	DIT1t-d22	0.997961	9.953160331		
	12h	DIT1t-d22	0.982986	9.615812802		
7	24h	PGK1t	0.454088	2.845037532	2.840197731	0.065597617
	24h	PGK1t	0.449321	2.813979958		
	24h	PGK1t	0.466608	2.928248975		
	24h	PGK1t	0.443032	2.773524458		
8	24h	wild-type DIT1t	0.873612	7.475013821	7.2601798	0.164387723
	24h	wild-type DIT1t	0.850248	7.08350166		
	24h	wild-type DIT1t	0.857486	7.202545326		
	24h	wild-type DIT1t	0.862111	7.279658393		
9	24h	DIT1t-d22	0.933091	8.572174437	8.462502943	0.209461461
	24h	DIT1t-d22	0.919786	8.313540178		
	24h	DIT1t-d22	0.939617	8.701958344		
	24h	DIT1t-d22	0.917103	8.262338811		
10	30h	PGK1t	0.446495	2.79572854	2.776173561	0.042392723
	30h	PGK1t	0.43577	2.727532914		
	30h	PGK1t	0.447973	2.80525923		
11	30h	wild-type DIT1t	0.91502	8.222805165	7.868619078	0.238606022
	30h	wild-type DIT1t	0.887798	7.723212778		
	30h	wild-type DIT1t	0.888151	7.729492854		
	30h	wild-type DIT1t	0.892037	7.798965515		
12	30h	DIT1t-d22	0.896519	7.879869044	8.050765051	0.140076714
	30h	DIT1t-d22	0.913952	8.202608807		
	30h	DIT1t-d22	0.909393	8.116952416		
	30h	DIT1t-d22	0.903287	8.003629937		

	2	3	significant
Mean	5.261716931	7.69845226	
Variance	0.03017999	0.037465886	
Observations	4	4	

Pearson Correlation	0.033822938	
Hypothesized Mean Differer	0	
df	6	
t Stat	-18.73776083	
P(T<=t) one-tail	7.45841E-07	
t Critical one-tail	3.142668403	
P(T<=t) two-tail	1.49168E-06	
t Critical two-tail	3.707428021	

	5	6	significant
Mean	6.734067819	9.920203597	
Variance	0.074222541	0.063807084	
Observations	4	4	
Pearson Correlation	0.069014813		
Hypothesized Mean Differer	0		
df	6		
t Stat	-17.15173745		
P(T<=t) one-tail	1.25719E-06		
t Critical one-tail	3.142668403		
P(T<=t) two-tail	2.51439E-06		
t Critical two-tail	3.707428021		

	8	9	significant
Mean	7.2601798	8.462502943	
Variance	0.027023323	0.043874103	
Observations	4	4	
Pearson Correlation	0.035448713		
Hypothesized Mean Differer	0		
df	6		
t Stat	-9.031002565		
P(T<=t) one-tail	5.1625E-05		
t Critical one-tail	3.142668403		
P(T<=t) two-tail	0.00010325		
t Critical two-tail	3.707428021		

	11	12	no difference
Mean	7.868619078	8.050765051	
Variance	0.056932834	0.019621486	
Observations	4	4	
Pearson Correlation	0.03827716		
Hypothesized Mean Differer	0		
df	6		
t Stat	-1.31663295		
P(T<=t) one-tail	0.118007896		
t Critical one-tail	3.142668403		
P(T<=t) two-tail	0.236015792		
t Critical two-tail	3.707428021		

Supplementary Table S5. Raw data sets of Supplementary Figures S3-S6 & S8.

Population	Genetic Background	Overexpressing plasmid	Average log10 value (a.u.) of fluorecent intensity (FI) from 3000 cells	Average value of FI	Mean value of Average FI	Standard Deviatoin of Average FI
1	DIT1t	pGP564	0.831781	6.788612196	6.7245	0.0782
	DIT1t	pGP564	0.825535	6.691677464		
	DIT1t	pGP564	0.821463	6.629228671		
	DIT1t	pGP564	0.831777	6.788549671		
2	DIT1t	pGP564-7F9	0.925055	8.415017045	8.5042	0.2468
	DIT1t	pGP564-7F9	0.938842	8.686443516		
	DIT1t	pGP564-7F9	0.913593	8.195831103		
	DIT1t	pGP564-7F9	0.940501	8.719689091		
3	DIT1t	pGP564-10A10	1.02041	10.48117567	10.6056	0.2733
	DIT1t	pGP564-10A10	1.01658	10.3891496		
	DIT1t	pGP564-10A10	1.02318	10.54823994		
	DIT1t	pGP564-10A10	1.04154	11.00373189		
4	DIT1t	pGP564-11E10	0.940168	8.713005737	8.6256	0.0942
	DIT1t	pGP564-11E10	0.939013	8.689864408		
	DIT1t	pGP564-11E10	0.934026	8.590649498		
	DIT1t	pGP564-11E10	0.92987	8.508833		
5	DIT1t	pGP564-12B3	1.06249	11.54755395	11.4103	0.3067
	DIT1t	pGP564-12B3	1.04371	11.05885083		
	DIT1t	pGP564-12B3	1.06537	11.62438539		

t-Test: Paired Assuming equal variances (p<0.01) significance

Population	1	2
Mean	6.724517	8.504245189
Variance	0.006122226	0.060897739
Observations	4	4
Pearson Correlation	0.033509982	
Hypothesized Mean Differe	0	
df	6	
t Stat	-13.74933134	
P(T<=t) one-tail	4.60204E-06	
t Critical one-tail	3.142668403	
P(T<=t) two-tail	9.20407E-06	
t Critical two-tail	3.707428021	

t-Test: Paired Assuming equal variances (p<0.01) significance

Population	1	3
Mean	6.724517	10.60557427
Variance	0.006122226	0.074710455
Observations	4	4
Pearson Correlation	0.04041634	
Hypothesized Mean Differe	0	
df	6	
t Stat	-27.30150304	
P(T<=t) one-tail	7.98019E-08	
t Critical one-tail	3.142668403	
P(T<=t) two-tail	1.59604E-07	
t Critical two-tail	3.707428021	

t-Test: Paired Assuming equal variances (p<0.01) significance

Population	1	4
Mean	6.724517	8.625588161
Variance	0.006122226	0.008875249
Observations	4	4

Pearson Correlation	0.007498737	
Hypothesized Mean Differe	0	
df	6	
t Stat	-31.04697562	
P(T<=t) one-tail	3.70752E-08	
t Critical one-tail	3.142668403	
P(T<=t) two-tail	7.41504E-08	
t Critical two-tail	3.707428021	

t-Test: Paired Assuming equal variances (p<0.01) significance

Population	1	5
Mean	6.724517	11.41026339
Variance	0.006122226	0.094093859
Observations	4	3
Pearson Correlation	0.041310879	
Hypothesized Mean Differe	0	
df	5	
t Stat	-30.18479039	
P(T<=t) one-tail	3.74317E-07	
t Critical one-tail	2.015048373	
P(T<=t) two-tail	7.48634E-07	
t Critical two-tail	2.570581836	

Population	Genetic Background	Overexpressing plasmid	Average log10 value (a.u.) of fluorecent intensity (FI) from 3000 cells	Average value of FI	Mean value of Average FI	Standard Deviatoin of Average FI
1	DIT1t	pGP564	0.782255	6.056964106	6.2198	0.1116
	DIT1t	pGP564	0.797779	6.277388392		
	DIT1t	pGP564	0.799615	6.303982516		
	DIT1t	pGP564	0.795251	6.240954265		
2	DIT1t	pGP564-NAB6	0.992469	9.828087175	9.8755	0.0375
	DIT1t	pGP564-NAB6	0.994002	9.862840276		
	DIT1t	pGP564-NAB6	0.995933	9.906790979		
	DIT1t	pGP564-NAB6	0.995828	9.904396089		
3	RPL41Bt	pGP564	0.836123	6.856823965	7.0635	0.1400
	RPL41Bt	pGP564	0.852424	7.119082077		
	RPL41Bt	pGP564	0.855328	7.166844803		
	RPL41Bt	pGP564	0.851944	7.111218123		
4	RPL41Bt	pGP564-NAB6	0.816559	6.554793283	6.4983	0.0380
	RPL41Bt	pGP564-NAB6	0.811212	6.474585943		
	RPL41Bt	pGP564-NAB6	0.811465	6.478358838		
	RPL41Bt	pGP564-NAB6	0.811929	6.485284012		
5	RPL15At	pGP564	0.816999	6.561437555	6.6307	0.0868
	RPL15At	pGP564	0.821423	6.628618125		
	RPL15At	pGP564	0.818161	6.57901687		
	RPL15At	pGP564	0.829545	6.753750303		
6	RPL15At	pGP564-NAB6	0.790798	6.177290138	5.8588	0.2834
	RPL15At	pGP564-NAB6	0.778881	6.010090339		
	RPL15At	pGP564-NAB6	0.754319	5.67961634		
	RPL15At	pGP564-NAB6	0.745712	5.568163768		
7	RPL3t	pGP564	0.817695	6.57196134	6.5113	0.0709
	RPL3t	pGP564	0.806805	6.409217355		
	RPL3t	pGP564	0.814536	6.524331209		
	RPL3t	pGP564	0.815552	6.53961227		
8	RPL3t	pGP564-NAB6	0.793214	6.211750448	6.1639	0.1036
	RPL3t	pGP564-NAB6	0.79666	6.261234939		
	RPL3t	pGP564-NAB6	0.789684	6.161465201		
	RPL3t	pGP564-NAB6	0.779672	6.02104676		
9	IDP1t	pGP564	0.785813	6.1067902	6.0425	0.0500
	IDP1t	pGP564	0.781208	6.042379524		
	IDP1t	pGP564	0.777058	5.984915182		
	IDP1t	pGP564	0.780751	6.036024585		
10	IDP1t	pGP564-NAB6	0.754442	5.681225138	5.4713	0.1659
	IDP1t	pGP564-NAB6	0.741737	5.517432135		
	IDP1t	pGP564-NAB6	0.731234	5.385598831		
	IDP1t	pGP564-NAB6	0.724357	5.300990184		
11	PGK1t	pGP564	0.477676	3.003834493	2.9421	0.0525
	PGK1t	pGP564	0.466297	2.926152795		
	PGK1t	pGP564	0.471119	2.958823096		
	PGK1t	pGP564	0.459323	2.879539233		
12	PGK1t	pGP564-NAB6	0.447889	2.804716697	2.8007	0.0231
	PGK1t	pGP564-NAB6	0.451942	2.831013889		
	PGK1t	pGP564-NAB6	0.443703	2.777812961		
	PGK1t	pGP564-NAB6	0.44546	2.789073761		

t-Test: Paired Assuming equal variances (p<0.01) significance

Population	1	2
Mean	6.21982232	9.87552863
Variance	0.012455383	0.00140745
Observations	4	4
Pearson Correlation	0.006931417	
Hypothesized Mean Differe	0	
df	6	
t Stat	-62.09766902	
P(T<=t) one-tail	5.86205E-10	

t Critical one-tail	3.142668403	
P(T<=t) two-tail	1.17241E-09	
t Critical two-tail	3.707428021	

t-Test: Paired Assuming equal variances (p<0.01) significance

Population	3	4
Mean	7.063492242	6.498255519
Variance	0.019587173	0.001440302
Observations	4	4
Pearson Correlation	0.010513737	
Hypothesized Mean Differe	0	
df	6	
t Stat	7.795909848	
P(T<=t) one-tail	0.000117385	
t Critical one-tail	3.142668403	
P(T<=t) two-tail	0.000234771	
t Critical two-tail	3.707428021	

t-Test: Paired Assuming equal variances (p<0.01) significance

Population	5	6
Mean	6.630705713	5.858790146
Variance	0.007538048	0.080300313
Observations	4	4
Pearson Correlation	0.04391918	
Hypothesized Mean Differe	0	
df	6	
t Stat	5.209039906	
P(T<=t) one-tail	0.000998632	
t Critical one-tail	3.142668403	
P(T<=t) two-tail	0.001997264	
t Critical two-tail	3.707428021	

t-Test: Paired Assuming equal variances (p<0.01) significance

Population	7	8
Mean	6.511280544	6.163874337
Variance	0.00502402	0.010725576
Observations	4	4
Pearson Correlation	0.007874798	
Hypothesized Mean Differe	0	
df	6	
t Stat	5.536468745	
P(T<=t) one-tail	0.000732176	
t Critical one-tail	3.142668403	
P(T<=t) two-tail	0.001464352	
t Critical two-tail	3.707428021	

t-Test: Paired Assuming equal variances (p<0.01) significance

Population	9	10
Mean	6.042527373	5.471311572
Variance	0.002497061	0.02751562
Observations	4	4
Pearson Correlation	0.015006341	
Hypothesized Mean Differe	0	
df	6	
t Stat	6.594438294	
P(T<=t) one-tail	0.000292159	
t Critical one-tail	3.142668403	
P(T<=t) two-tail	0.000584318	
t Critical two-tail	3.707428021	

t-Test: Paired Assuming equal variances (p<0.01) significance

Population	11	12
Mean	2.942087404	2.800654327
Variance	0.002752991	0.000531348
Observations	4	4
Pearson Correlation	0.001642169	
Hypothesized Mean Differe	0	
df	6	
t Stat	4.935794117	
P(T<=t) one-tail	0.001307475	
t Critical one-tail	3.142668403	
P(T<=t) two-tail	0.00261495	
t Critical two-tail	3.707428021	

Population	Genetic Background	Overexpressing plasmid	Average log10 value (a.u.) of fluorecent intensity (FI) from 3000 cells	Average value of FI	Mean value of Average FI	Standard Deviatoin of Average FI
	DIT1t	pGP564	0.74054	5.502245957	5.6187	0.1054
1	DIT1t	pGP564	0.756453	5.707593044		
	DIT1t	pGP564	0.751759	5.646235653		
	DIT1t	pGP564-PAP1	1.11476	13.02446821	12.6206	0.3562
2	DIT1t	pGP564-PAP1	1.0917	12.35093966		
	DIT1t	pGP564-PAP1	1.09644	12.48647926		
	RPL41Bt	pGP564	0.823009	6.65286943	6.7972	0.3818
3	RPL41Bt	pGP564	0.859151	7.230211474		
	RPL41Bt	pGP564	0.81349	6.50863624		
	RPL41Bt	pGP564-PAP1	0.840526	6.926693964	6.9242	0.2392
4	RPL41Bt	pGP564-PAP1	0.825024	6.683808526		
	RPL41Bt	pGP564-PAP1	0.855045	7.162176182		
	RPL15At	pGP564	0.758117	5.729503644	5.9172	0.3630
5	RPL15At	pGP564	0.801789	6.335618233		
	RPL15At	pGP564	0.754854	5.686617275		
	RPL15At	pGP564-PAP1	0.725836	5.319073604	5.6502	0.4693
6	RPL15At	pGP564-PAP1	0.735946	5.444349538		
	RPL15At	pGP564-PAP1	0.791498	6.187254782		
	RPL3t	pGP564	0.793287	6.212794661	6.1034	0.1428
7	RPL3t	pGP564	0.773923	5.941868005		
	RPL3t	pGP564	0.789274	6.155651154		
	RPL3t	pGP564-PAP1	0.801583	6.332613756	6.2525	0.1708
8	RPL3t	pGP564-PAP1	0.782215	6.056406265		
	RPL3t	pGP564-PAP1	0.80404	6.368541746		
	IDP1t	pGP564	0.759239	5.744324957	5.9796	0.3339
9	IDP1t	pGP564	0.765872	5.832731703		
	IDP1t	pGP564	0.803579	6.361785179		
	IDP1t	pGP564-PAP1	0.745316	5.563088898	5.5381	0.0638
10	IDP1t	pGP564-PAP1	0.737633	5.46553903		
	IDP1t	pGP564-PAP1	0.747074	5.585653613		
	PGK1t	pGP564	0.437995	2.741542609	2.8000	0.0833
11	PGK1t	pGP564	0.441386	2.763032543		
	PGK1t	pGP564	0.461715	2.895442868		
	PGK1t	pGP564-PAP1	0.420156	2.631212962	2.6284	0.0251
12	PGK1t	pGP564-PAP1	0.415298	2.601944328		
	PGK1t	pGP564-PAP1	0.423558	2.651905231		

t-Test: Paired Assuming equal variances ($p<0.01$) significance

Population	1	2
Mean	5.618691551	12.62062904
Variance	0.011110865	0.1269073
Observations	3	3
Pearson Correlation	0.069009082	
Hypothesized Mean Differe	0	
df	4	
t Stat	-32.64455349	
P(T<=t) one-tail	2.62522E-06	
t Critical one-tail	3.746947388	
P(T<=t) two-tail	5.25045E-06	
t Critical two-tail	4.604094871	

t-Test: Paired Assuming equal variances ($p<0.01$) No

Population	3	4
Mean	6.797239048	6.924226224
Variance	0.145799645	0.057213471
Observations	3	3
Pearson Correlation	0.101506558	
Hypothesized Mean Differe	0	

df	4	
t Stat	-0.488155791	
P(T<=t) one-tail	0.325495153	
t Critical one-tail	3.746947388	
P(T<=t) two-tail	0.650990305	
t Critical two-tail	4.604094871	

t-Test: Paired Assuming equal variances (p<0.01) No

Population	5	6
Mean	5.917246384	5.650225975
Variance	0.131736063	0.22022347
Observations	3	3
Pearson Correlation	0.175979767	
Hypothesized Mean Differe	0	
df	4	
t Stat	0.779576463	
P(T<=t) one-tail	0.239594734	
t Critical one-tail	3.746947388	
P(T<=t) two-tail	0.479189469	
t Critical two-tail	4.604094871	

t-Test: Paired Assuming equal variances (p<0.01) No

Population	7	8
Mean	6.10343794	6.252520589
Variance	0.020394978	0.029168326
Observations	3	3
Pearson Correlation	0.024781652	
Hypothesized Mean Differe	0	
df	4	
t Stat	-1.159865424	
P(T<=t) one-tail	0.155309128	
t Critical one-tail	3.746947388	
P(T<=t) two-tail	0.310618255	
t Critical two-tail	4.604094871	

t-Test: Paired Assuming equal variances (p<0.01) No

Population	9	10
Mean	5.979613946	5.538093847
Variance	0.111495076	0.004075443
Observations	3	3
Pearson Correlation	0.05778526	
Hypothesized Mean Differe	0	
df	4	
t Stat	2.249508166	
P(T<=t) one-tail	0.043846467	
t Critical one-tail	3.746947388	
P(T<=t) two-tail	0.087692934	
t Critical two-tail	4.604094871	

t-Test: Paired Assuming equal variances (p<0.01) No

Population	11	12
Mean	2.800006006	2.628354174
Variance	0.0069466	0.000630152
Observations	3	3
Pearson Correlation	0.003788376	
Hypothesized Mean Differe	0	
df	4	
t Stat	3.415604014	
P(T<=t) one-tail	0.013444261	
t Critical one-tail	3.746947388	
P(T<=t) two-tail	0.026888522	

t Critical two-tail	4.604094871

Population	Genetic Background	Overexpressing plasmid	Average log10 value (a.u.) of fluorecent intensity (FI) from 3000 cells	Average value of FI	Mean value of Average FI	Standard Deviatoin of Average FI
1	DIT1t	pGP564	0.851408	7.102446974	6.9596	0.1484
	DIT1t	pGP564	0.834463	6.830665212		
	DIT1t	pGP564	0.849601	7.07295671		
	DIT1t	pGP564	0.834572	6.8323798		
2	DIT1t	pGP564-MOS2	0.915968	8.240773925	8.2712	0.3537
	DIT1t	pGP564-MOS2	0.941153	8.732789665		
	DIT1t	pGP564-MOS2	0.915963	8.24067905		
	DIT1t	pGP564-MOS2	0.896016	7.870747861		
3	RPL41Bt	pGP564	0.83918	6.905259436	6.9711	0.0679
	RPL41Bt	pGP564	0.842662	6.960845589		
	RPL41Bt	pGP564	0.84211	6.952003787		
	RPL41Bt	pGP564	0.84918	7.066103591		
4	RPL41Bt	pGP564-MOS2	0.850863	7.09353964	7.0069	0.1624
	RPL41Bt	pGP564-MOS2	0.85573	7.173481787		
	RPL41Bt	pGP564-MOS2	0.842424	6.957031984		
	RPL41Bt	pGP564-MOS2	0.832737	6.803572233		
5	RPL15At	pGP564	0.81193	6.485298945	6.4924	0.0368
	RPL15At	pGP564	0.815887	6.544658651		
	RPL15At	pGP564	0.811658	6.481238453		
	RPL15At	pGP564	0.810124	6.458386029		
6	RPL15At	pGP564-MOS2	0.81321	6.504441319	6.4683	0.0521
	RPL15At	pGP564-MOS2	0.81133	6.476345359		
	RPL15At	pGP564-MOS2	0.812924	6.500159299		
	RPL15At	pGP564-MOS2	0.805665	6.392415561		
7	RPL3t	pGP564	0.809232	6.445134725	6.4113	0.0479
	RPL3t	pGP564	0.809936	6.455590889		
	RPL3t	pGP564	0.805618	6.391723801		
	RPL3t	pGP564	0.802974	6.352928975		
8	RPL3t	pGP564-MOS2	0.800679	6.319445896	6.3788	0.1005
	RPL3t	pGP564-MOS2	0.811873	6.484447822		
	RPL3t	pGP564-MOS2	0.797299	6.270454199		
	RPL3t	pGP564-MOS2	0.808946	6.440891747		
9	IDP1t	pGP564	0.768643	5.870066213	5.8324	0.1618
	IDP1t	pGP564	0.767697	5.857293683		
	IDP1t	pGP564	0.74881	5.608025762		
	IDP1t	pGP564	0.777724	5.99410022		
10	IDP1t	pGP564-MOS2	0.777173	5.986500181	5.9583	0.0680
	IDP1t	pGP564-MOS2	0.778119	5.999554461		
	IDP1t	pGP564-MOS2	0.767649	5.856646347		
	IDP1t	pGP564-MOS2	0.777453	5.990361065		
11	PGK1t	pGP564	0.45885	2.876404768	2.8424	0.0445
	PGK1t	pGP564	0.456185	2.858808074		
	PGK1t	pGP564	0.455982	2.857472109		
	PGK1t	pGP564	0.443556	2.776872886		
12	PGK1t	pGP564-MOS2	0.527869	3.371855851	3.1000	0.1828
	PGK1t	pGP564-MOS2	0.482969	3.040667975		
	PGK1t	pGP564-MOS2	0.477597	3.003288133		
	PGK1t	pGP564-MOS2	0.474824	2.984173023		

t-Test: Paired Assuming equal variances ($p<0.01$) significance

Population	1	2
Mean	6.959612174	8.271247625
Variance	0.022021387	0.125094733
Observations	4	4
Pearson Correlation	0.07355806	
Hypothesized Mean Differe	0	
df	6	
t Stat	-6.839321368	
P(T<=t) one-tail	0.000240105	

t Critical one-tail	3.142668403	
P(T<=t) two-tail	0.000480211	
t Critical two-tail	3.707428021	

t-Test: Paired Assuming equal variances (p<0.01) No

Population	3	4
Mean	6.971053101	7.006906411
Variance	0.004610157	0.02636164
Observations	4	4
Pearson Correlation	0.015485898	
Hypothesized Mean Differe	0	
df	6	
t Stat	-0.407451628	
P(T<=t) one-tail	0.348907522	
t Critical one-tail	3.142668403	
P(T<=t) two-tail	0.697815043	
t Critical two-tail	3.707428021	

t-Test: Paired Assuming equal variances (p<0.01) No

Population	5	6
Mean	6.492395519	6.468340385
Variance	0.001354307	0.002714793
Observations	4	4
Pearson Correlation	0.00203455	
Hypothesized Mean Differe	0	
df	6	
t Stat	0.754203578	
P(T<=t) one-tail	0.239635171	
t Critical one-tail	3.142668403	
P(T<=t) two-tail	0.479270343	
t Critical two-tail	3.707428021	

t-Test: Paired Assuming equal variances (p<0.01) No

Population	7	8
Mean	6.411344598	6.378809916
Variance	0.002298956	0.010092856
Observations	4	4
Pearson Correlation	0.006195906	
Hypothesized Mean Differe	0	
df	6	
t Stat	0.584533142	
P(T<=t) one-tail	0.290080239	
t Critical one-tail	3.142668403	
P(T<=t) two-tail	0.580160478	
t Critical two-tail	3.707428021	

t-Test: Paired Assuming equal variances (p<0.01) No

Population	9	10
Mean	5.83237147	5.958265514
Variance	0.026176399	0.004619518
Observations	4	4
Pearson Correlation	0.015397958	
Hypothesized Mean Differe	0	
df	6	
t Stat	-1.434790905	
P(T<=t) one-tail	0.100674642	
t Critical one-tail	3.142668403	
P(T<=t) two-tail	0.201349284	
t Critical two-tail	3.707428021	

t-Test: Paired Assuming equal variances (p<0.01) No

Population	11	12
Mean	2.842389459	3.099996245
Variance	0.001982173	0.033398322
Observations	4	4
Pearson Correlation	0.017690248	
Hypothesized Mean Differe	0	
df	6	
t Stat	-2.739083925	
P(T<=t) one-tail	0.016888344	
t Critical one-tail	3.142668403	
P(T<=t) two-tail	0.033776687	
t Critical two-tail	3.707428021	

Population	Genetic Background	Overexpressing plasmid	Average log10 value (a.u.) of fluorecent intensity (FI) from 3000 cells	Average value of FI	Mean value of Average FI	Standard Deviatoin of Average FI
1	DIT1t	pGP564	0.782255	6.056964106	6.2198	0.1116
	DIT1t	pGP564	0.797779	6.277388392		
	DIT1t	pGP564	0.799615	6.303982516		
	DIT1t	pGP564	0.795251	6.240954265		
2	DIT1t	pGP564-CCC1	0.870018	7.413409667	7.8420	0.3142
	DIT1t	pGP564-CCC1	0.911815	8.162345991		
	DIT1t	pGP564-CCC1	0.894929	7.85107272		
	DIT1t	pGP564-CCC1	0.899887	7.941215837		
3	RPL41Bt	pGP564	0.836123	6.856823965	7.0635	0.1400
	RPL41Bt	pGP564	0.852424	7.119082077		
	RPL41Bt	pGP564	0.855328	7.166844803		
	RPL41Bt	pGP564	0.851944	7.111218123		
4	RPL41Bt	pGP564-CCC1	0.862097	7.279423729	7.4710	0.1684
	RPL41Bt	pGP564-CCC1	0.869133	7.398318093		
	RPL41Bt	pGP564-CCC1	0.884613	7.666779978		
	RPL41Bt	pGP564-CCC1	0.877352	7.539664134		
5	RPL15At	pGP564	0.816999	6.561437555	6.6307	0.0868
	RPL15At	pGP564	0.821423	6.628618125		
	RPL15At	pGP564	0.818161	6.57901687		
	RPL15At	pGP564	0.829545	6.753750303		
6	RPL15At	pGP564-CCC1	0.841418	6.940935362	6.9839	0.0509
	RPL15At	pGP564-CCC1	0.847976	7.046541273		
	RPL15At	pGP564-CCC1	0.845352	7.004094548		
	RPL15At	pGP564-CCC1	0.841603	6.943892679		
7	RPL3t	pGP564	0.817695	6.57196134	6.5113	0.0709
	RPL3t	pGP564	0.806805	6.409217355		
	RPL3t	pGP564	0.814536	6.524331209		
	RPL3t	pGP564	0.815552	6.53961227		
8	RPL3t	pGP564-CCC1	0.836249	6.858813595	6.9456	0.0791
	RPL3t	pGP564-CCC1	0.838852	6.900046222		
	RPL3t	pGP564-CCC1	0.844983	6.99814602		
	RPL3t	pGP564-CCC1	0.846683	7.02559321		
9	IDP1t	pGP564	0.785813	6.1067902	6.0425	0.0500
	IDP1t	pGP564	0.781208	6.042379524		
	IDP1t	pGP564	0.777058	5.984915182		
	IDP1t	pGP564	0.780751	6.036024585		
10	IDP1t	pGP564-CCC1	0.817524	6.569374192	6.4619	0.0799
	IDP1t	pGP564-CCC1	0.810523	6.464322278		
	IDP1t	pGP564-CCC1	0.808539	6.43485848		
	IDP1t	pGP564-CCC1	0.804765	6.379182105		
11	PGK1t	pGP564	0.477676	3.003834493	2.9421	0.0525
	PGK1t	pGP564	0.466297	2.926152795		
	PGK1t	pGP564	0.471119	2.958823096		
	PGK1t	pGP564	0.459323	2.879539233		
12	PGK1t	pGP564-CCC1	0.496049	3.133639262	3.1160	0.0219
	PGK1t	pGP564-CCC1	0.493143	3.1127411		
	PGK1t	pGP564-CCC1	0.495741	3.131417685		
	PGK1t	pGP564-CCC1	0.48945	3.086384292		

t-Test: Paired Assuming equal variances (p<0.01) significance

Population	1	2
Mean	6.21982232	7.842011054
Variance	0.012455383	0.098745775
Observations	4	4
Pearson Correlation	0.055600579	
Hypothesized Mean Differe	0	
df	6	
t Stat	-9.729190853	
P(T<=t) one-tail	3.38606E-05	

t Critical one-tail	3.142668403
P(T<=t) two-tail	6.77212E-05
t Critical two-tail	3.707428021

t-Test: Paired Assuming equal variances (p<0.01) significance

Population	3	4
Mean	7.063492242	7.471046483
Variance	0.019587173	0.028342894
Observations	4	4
Pearson Correlation	0.023965033	
Hypothesized Mean Differe	0	
df	6	
t Stat	-3.723157398	
P(T<=t) one-tail	0.004907837	
t Critical one-tail	3.142668403	
P(T<=t) two-tail	0.009815674	
t Critical two-tail	3.707428021	

t-Test: Paired Assuming equal variances (p<0.01) significance

Population	5	6
Mean	6.630705713	6.983865965
Variance	0.007538048	0.002592763
Observations	4	4
Pearson Correlation	0.005065406	
Hypothesized Mean Differe	0	
df	6	
t Stat	-7.017456108	
P(T<=t) one-tail	0.000208899	
t Critical one-tail	3.142668403	
P(T<=t) two-tail	0.000417798	
t Critical two-tail	3.707428021	

t-Test: Paired Assuming equal variances (p<0.01) significance

Population	7	8
Mean	6.511280544	6.945649762
Variance	0.00502402	0.006255672
Observations	4	4
Pearson Correlation	0.005639846	
Hypothesized Mean Differe	0	
df	6	
t Stat	-8.179757413	
P(T<=t) one-tail	8.99127E-05	
t Critical one-tail	3.142668403	
P(T<=t) two-tail	0.000179825	
t Critical two-tail	3.707428021	

t-Test: Paired Assuming equal variances (p<0.01) significance

Population	9	10
Mean	6.042527373	6.461934263
Variance	0.002497061	0.006376686
Observations	4	4
Pearson Correlation	0.004436874	
Hypothesized Mean Differe	0	
df	6	
t Stat	-8.904550993	
P(T<=t) one-tail	5.58943E-05	
t Critical one-tail	3.142668403	
P(T<=t) two-tail	0.000111789	
t Critical two-tail	3.707428021	

t-Test: Paired Assuming equal variances (p<0.01) significance

Population	11	12
Mean	2.942087404	3.116045585
Variance	0.002752991	0.00047885
Observations	4	4
Pearson Correlation	0.00161592	
Hypothesized Mean Differe	0	
df	6	
t Stat	-6.119978058	
P(T<=t) one-tail	0.000434572	
t Critical one-tail	3.142668403	
P(T<=t) two-tail	0.000869143	
t Critical two-tail	3.707428021	

Population	Genetic Background	NAB6 Background	Average log10 value (a.u.) of fluorecent intensity (FI) from 3000 cells	Average value of FI	Mean value of Average FI	Standard Deviatoin of Average FI
1	DIT1t	+	0.86939	7.40269745	7.1912	0.1896
	DIT1t	+	0.841491	6.942102153		
	DIT1t	+	0.857133	7.196693385		
	DIT1t	+	0.858747	7.223488738		
2	DIT1t	−	0.650996	4.477091806	4.5534	0.0890
	DIT1t	−	0.669916	4.67644682		
	DIT1t	−	0.653291	4.500813319		
	DIT1t	−	0.658895	4.559266726		
3	RPL41Bt	+	0.917793	8.275476314	8.2119	0.0721
	RPL41Bt	+	0.915656	8.234855825		
	RPL41Bt	+	0.908928	8.108266229		
	RPL41Bt	+	0.915345	8.228960923		
4	RPL41Bt	−	0.908115	8.093101739	8.1514	0.0518
	RPL41Bt	−	0.914587	8.214610955		
	RPL41Bt	−	0.912055	8.166857917		
	RPL41Bt	−	0.910144	8.131000729		
5	RPL15At	+	0.884719	7.668651468	7.8513	0.1823
	RPL15At	+	0.889086	7.746151737		
	RPL15At	+	0.89828	7.911885617		
	RPL15At	+	0.907326	8.078412028		
6	RPL15At	−	0.89639	7.877528807	7.8881	0.0307
	RPL15At	−	0.894845	7.849554335		
	RPL15At	−	0.89848	7.915530014		
	RPL15At	−	0.89817	7.909881914		
7	RPL3t	+	0.877731	7.546246718	7.6817	0.1119
	RPL3t	+	0.893194	7.819770369		
	RPL3t	+	0.884896	7.671777522		
	RPL3t	+	0.885865	7.68891394		
8	RPL3t	−	0.879466	7.576454161	7.6635	0.1247
	RPL3t	−	0.883337	7.644287282		
	RPL3t	−	0.894606	7.845235773		
	RPL3t	−	0.880123	7.587924483		
9	IDP1t	+	0.853582	7.138089681	7.1148	0.0807
	IDP1t	+	0.854121	7.146954216		
	IDP1t	+	0.844877	6.996438162		
	IDP1t	+	0.855985	7.177695		
10	IDP1t	−	0.860495	7.252621282	7.2635	0.1287
	IDP1t	−	0.870574	7.422906668		
	IDP1t	−	0.861541	7.270110302		
	IDP1t	−	0.851762	7.108238646		
11	PGK1t	+	0.522942	3.333818867	3.4213	0.0643
	PGK1t	+	0.533648	3.417023771		
	PGK1t	+	0.541976	3.483180657		
	PGK1t	+	0.53797	3.451198985		
12	PGK1t	−	0.535414	3.430946927	3.3638	0.0462
	PGK1t	−	0.523616	3.338996778		
	PGK1t	−	0.522309	3.328963244		
	PGK1t	−	0.525848	3.35620129		

t-Test: Paired Assuming equal variances (p<0.01) significance

Population	1	2
Mean	7.191245431	4.553404668
Variance	0.035951213	0.007921079
Observations	4	4
Pearson Correlation	0.021936146	
Hypothesized Mean Differe	0	
df	6	
t Stat	25.18740384	
P(T<=t) one-tail	1.28956E-07	

t Critical one-tail	3.142668403	
P(T<=t) two-tail	2.57912E-07	
t Critical two-tail	3.707428021	

t-Test: Paired Assuming equal variances (p<0.01) No

Population	3	4
Mean	8.211889823	8.151392835
Variance	0.005199984	0.00268313
Observations	4	4
Pearson Correlation	0.003941557	
Hypothesized Mean Differe	0	
df	6	
t Stat	1.362745833	
P(T<=t) one-tail	0.110945156	
t Critical one-tail	3.142668403	
P(T<=t) two-tail	0.221890312	
t Critical two-tail	3.707428021	

t-Test: Paired Assuming equal variances (p<0.01) No

Population	5	6
Mean	7.851275213	7.888123767
Variance	0.033222377	0.000941458
Observations	4	4
Pearson Correlation	0.017081918	
Hypothesized Mean Differe	0	
df	6	
t Stat	-0.398719198	
P(T<=t) one-tail	0.351951315	
t Critical one-tail	3.142668403	
P(T<=t) two-tail	0.703902629	
t Critical two-tail	3.707428021	

t-Test: Paired Assuming equal variances (p<0.01) No

Population	7	8
Mean	7.681677137	7.663475425
Variance	0.012520504	0.015561885
Observations	4	4
Pearson Correlation	0.014041195	
Hypothesized Mean Differe	0	
df	6	
t Stat	0.217232714	
P(T<=t) one-tail	0.417612802	
t Critical one-tail	3.142668403	
P(T<=t) two-tail	0.835225605	
t Critical two-tail	3.707428021	

t-Test: Paired Assuming equal variances (p<0.01) No

Population	9	10
Mean	7.114794265	7.263469224
Variance	0.006513869	0.016559538
Observations	4	4
Pearson Correlation	0.011536704	
Hypothesized Mean Differe	0	
df	6	
t Stat	-1.95754593	
P(T<=t) one-tail	0.049014042	
t Critical one-tail	3.142668403	
P(T<=t) two-tail	0.098028084	
t Critical two-tail	3.707428021	

t-Test: Paired Assuming equal variances (p<0.01) No

Population	11	12
Mean	3.42130557	3.36377706
Variance	0.004131467	0.002131749
Observations	4	4
Pearson Correlation	0.003131608	
Hypothesized Mean Differe	0	
df	6	
t Stat	1.453832715	
P(T<=t) one-tail	0.09810977	
t Critical one-tail	3.142668403	
P(T<=t) two-tail	0.196219539	
t Critical two-tail	3.707428021	

Population	Genetic Background	Overexpression	Average log10 value (a.u.) of fluorecent intensity (FI) from 3000 cells	Average value of FI	Mean value of Average FI	Standard Deviatoin of Average FI
1	DIT1t	–	0.815773	6.542940938	6.46687263	0.056845395
	DIT1t	–	0.810309	6.461137747		
	DIT1t	–	0.806535	6.405233996		
	DIT1t	–	0.81011	6.458177839		
2	DIT1t	NAB6	1.01681	10.39465309	9.626356691	0.871659614
	DIT1t	NAB6	0.994123	9.865588573		
	DIT1t	NAB6	0.994421	9.87236037		
	DIT1t	NAB6	0.922872	8.372824727		
3	DIT1t	PAP1	1.15389	14.25246555	14.51142591	0.547921558
	DIT1t	PAP1	1.18496	15.3094645		
	DIT1t	PAP1	1.15843	14.40223855		
	DIT1t	PAP1	1.14865	14.08153503		
4	DIT1t-d1	–	0.801358	6.329333795	6.115034915	0.254701768
	DIT1t-d1	–	0.765925	5.833443556		
	DIT1t-d1	–	0.791152	6.182327394		
	DIT1t-d1	–	0.772189	,		
5	DIT1t-d1	NAB6	0.941243	8.734599571	8.346120242	0.297237881
	DIT1t-d1	NAB6	0.925387	8.421452434		
	DIT1t-d1	NAB6	0.91038	8.135420397		
	DIT1t-d1	NAB6	0.90811	8.093008565		
6	DIT1t-d1	PAP1	1.03247	10.77630811	11.46956545	0.564937876
	DIT1t-d1	PAP1	1.05442	11.33496021		
	DIT1t-d1	PAP1	1.08366	12.12439286		
	DIT1t-d1	PAP1	1.06605	11.64260062		
7	DIT1t-d2	–	0.600256	3.983419087	4.006769761	0.182242022
	DIT1t-d2	–	0.594434	3.930375102		
	DIT1t-d2	–	0.630096	4.266738238		
	DIT1t-d2	–	0.585071	3.846546616		
8	DIT1t-d2	NAB6	0.706846	5.091502953	4.813025064	0.193996224
	DIT1t-d2	NAB6	0.679318	4.778790592		
	DIT1t-d2	NAB6	0.666915	4.644243693		
	DIT1t-d2	NAB6	0.675555	4.737563019		
9	DIT1t-d2	PAP1	0.668713	4.663510941	4.63822091	0.062076316
	DIT1t-d2	PAP1	0.67	4.677351413		
	DIT1t-d2	PAP1	0.66899	4.666486352		
	DIT1t-d2	PAP1	0.657585	4.545534936		
10	DIT1t-d3	–	0.773664	5.938325513	5.960035868	0.325318917
	DIT1t-d3	–	0.756913	5.713641667		
	DIT1t-d3	–	0.807943	6.426033718		
	DIT1t-d3	–	0.760584	5.762142575		
11	DIT1t-d3	NAB6	0.951894	8.951462569	8.521697562	0.531157073
	DIT1t-d3	NAB6	0.892672	7.810377047		
	DIT1t-d3	NAB6	0.925393	8.421568781		
	DIT1t-d3	NAB6	0.949555	8.903381852		
12	DIT1t-d3	PAP1	1.0132	10.3086074	10.61147848	0.343492631
	DIT1t-d3	PAP1	1.01859	10.43734406		
	DIT1t-d3	PAP1	1.04505	11.09302521		
	DIT1t-d3	PAP1	1.02559	10.60693726		
13	DIT1t-d4	–	0.899677	7.937376847	6.815641846	0.749247835
	DIT1t-d4	–	0.810849	6.469176496		
	DIT1t-d4	–	0.804595	6.37668553		
	DIT1t-d4	–	0.81153	6.479328513		
14	DIT1t-d4	NAB6	0.979901	9.547749146	9.851669014	0.472139736
	DIT1t-d4	NAB6	1.01072	10.24990877		
	DIT1t-d4	NAB6	1.01111	10.25911741		
	DIT1t-d4	NAB6	0.970807	9.349900732		

15	DIT1t-d4	PAP1	1.12366	13.29413241	13.24800553	0.698135631
	DIT1t-d4	PAP1	1.15286	14.21870356		
	DIT1t-d4	PAP1	1.10585	12.75998019		
	DIT1t-d4	PAP1	1.10446	12.71920596		
16	DIT1t-d5	–	0.618537	4.154674458	4.401465465	0.226364361
	DIT1t-d5	–	0.666907	4.644158144		
	DIT1t-d5	–	0.656455	4.533723186		
	DIT1t-d5	–	0.630764	4.273306073		
17	DIT1t-d5	NAB6	0.836239	6.858655667	6.420838515	0.385552492
	DIT1t-d5	NAB6	0.792873	6.206875011		
	DIT1t-d5	NAB6	0.820317	6.611758771		
	DIT1t-d5	NAB6	0.77859	6.006064613		
18	DIT1t-d5	PAP1	0.935215	8.614200977	8.33677006	0.197621342
	DIT1t-d5	PAP1	0.921303	8.34263033		
	DIT1t-d5	PAP1	0.913339	8.191039118		
	DIT1t-d5	PAP1	0.913772	8.199209815		
19	DIT1t-d6	–	0.808941	6.440817594	6.502596547	0.77246815
	DIT1t-d6	–	0.881372	7.609778223		
	DIT1t-d6	–	0.77057	5.896170044		
	DIT1t-d6	–	0.782732	6.063620325		
20	DIT1t-d6	NAB6	0.940036	8.710357898	8.861682643	0.274490225
	DIT1t-d6	NAB6	0.941952	8.748870738		
	DIT1t-d6	NAB6	0.967202	9.272610128		
	DIT1t-d6	NAB6	0.940262	8.71489181		
21	DIT1t-d6	PAP1	1.01938	10.45634731	10.9111431	0.758439393
	DIT1t-d6	PAP1	1.00675	10.15663861		
	DIT1t-d6	PAP1	1.04865	11.18536087		
	DIT1t-d6	PAP1	1.07358	11.84622562		

Population	Genetic Background	Overexpression	Average log10 value (a.u.) of fluorecent intensity (FI) from 3000 cells	Average value of FI	Mean value of Average FI	Standard Deviatoin of Average FI	Relative FI (Overexpression−− Mean value as a standard)	Mean value of relative FI	SD of relative FI	t-Test: Two-Sample Assuming Equal Variances(P<0.01)			
1	ASP3t	–	0.172862	1.488887898	1.537397778	0.101456939	0.968446761	1.00	0.07	Population	1	4	significant
	ASP3t	–	0.227742	1.689436995			1.09889387			Mean	1	1.318907875	
	ASP3t	–	0.170415	1.480522459			0.963005463			Variance	0.004355029	7.67515E-05	
	ASP3t	–	0.173403	1.49074376			0.969653905			Observations	4	3	
2	ASP3t	NAB6	0.245337	1.759288241	1.742315452	0.032072375	1.144328596	1.13	0.02	Pooled Variance	0.002643718		
	ASP3t	NAB6	0.229254	1.695329035			1.102726347			Hypothesized Mean D	0		
	ASP3t	NAB6	0.242781	1.748964523			1.137613536			df	5		
	ASP3t	NAB6	0.246912	1.765680009			1.14848612			t Stat	-8.120809153		
3	ASP3t	PAP1	0.2382	1.730613153	1.746732296	0.03326792	1.125676892	1.14	0.02	P(T<=t) one-tail	0.000229713		
	ASP3t	PAP1	0.254445	1.796573544			1.168580812			t Critical one-tail	3.364929999		
	ASP3t	PAP1	0.238506	1.731832957			1.126470313			P(T<=t) two-tail	0.000459427		
	ASP3t	PAP1	0.237521	1.72790953			1.123918322			t Critical two-tail	4.032142984		
4	ASP3t	NAB6+PAP1	0.304561	2.016327159	2.027686037	0.013468823	1.311519496	1.32	0.01				
	ASP3t	NAB6+PAP1	0.310176	2.042565537			1.328586242						
	ASP3t	NAB6+PAP1	0.306246	2.024165414			1.316617887						
5	PIR1t	–	-0.00524738	0.987990162	0.978883875	0.031766884	1.009302725	1.00	0.03	Population	5	8	significant
	PIR1t	–	-0.0262735	0.941296622			0.961601929			Mean	1	1.531957965	
	PIR1t	–	-0.0134989	0.969395725			0.990307175			Variance	0.001053142	0.022209371	
	PIR1t	–	0.00725817	1.01685299			1.038788171			Observations	4	4	
6	PIR1t	NAB6	0.125369	1.334654945	1.353341129	0.015193116	1.363445634	1.38	0.02	Pooled Variance	0.011631256		
	PIR1t	NAB6	0.131183	1.35264241			1.381821118			Hypothesized Mean D	0		
	PIR1t	NAB6	0.131693	1.354231776			1.383444769			df	6		
	PIR1t	NAB6	0.137302	1.371835382			1.401428114			t Stat	-6.9755587		
7	PIR1t	PAP1	0.064807	1.160932582	1.159751315	0.023267836	1.185975796	1.18	0.02	P(T<=t) one-tail	0.000215797		
	PIR1t	PAP1	0.0763643	1.192241679			1.217960281			t Critical one-tail	3.142668403		
	PIR1t	PAP1	0.057875	1.142549435			1.167196094			P(T<=t) two-tail	0.000431594		
	PIR1t	PAP1	0.0581532	1.143281563			1.167944016			t Critical two-tail	3.707428021		
8	PIR1t	NAB6+PAP1	0.157916	1.438520317	1.499608949	0.145881192	1.469551552	1.53	0.15				
	PIR1t	NAB6+PAP1	0.123341	1.328437113			1.357093673						
	PIR1t	NAB6+PAP1	0.196431	1.571922028			1.605830956						
	PIR1t	NAB6+PAP1	0.219992	1.659556337			1.69535568						
9	FMP52t	–	-0.00786945	0.982043103	0.997261057	0.02892311	0.98474025	1.00	0.03	Population	9	12	significant
	FMP52t	–	0.0141387	1.03309129			1.035928639			Mean	1	1.602123333	
	FMP52t	–	-0.0144911	0.967183547			0.969839883			Variance	0.000841148	9.18678E-05	
	FMP52t	–	0.00291141	1.00672629			1.009491228			Observations	4	4	
10	FMP52t	NAB6	0.0986559	1.255035181	1.217656235	0.047652675	1.258482091	1.22	0.05	Pooled Variance	0.000466508		
	FMP52t	NAB6	0.0991831	1.256559622			1.260010719			Hypothesized Mean D	0		
	FMP52t	NAB6	0.0634242	1.157242034			1.160420359			df	6		
	FMP52t	NAB6	0.0798279	1.201788102			1.205088771			t Stat	-39.42493718		
11	FMP52t	PAP1	0.149968	1.412433469	1.355776649	0.051799439	1.416312668	1.36	0.05	P(T<=t) one-tail	8.89724E-09		
	FMP52t	PAP1	0.11456	1.30184716			1.305422638			t Critical one-tail	3.142668403		
	FMP52t	PAP1	0.121681	1.323369131			1.327003718			P(T<=t) two-tail	1.77945E-08		
	FMP52t	PAP1	0.141593	1.385456836			1.389261945			t Critical two-tail	3.707428021		
12	FMP52t	NAB6+PAP1	0.202336	1.593441046	1.59773521	0.009558519	1.597817376	1.60	0.01				
	FMP52t	NAB6+PAP1	0.202221	1.593019163			1.597394334						
	FMP52t	NAB6+PAP1	0.207381	1.61205925			1.616486714						
	FMP52t	NAB6+PAP1	0.202058	1.592421381			1.59679491						
13	MFA1t	–	0.707507	5.099258165	5.182590192	0.067473767	0.983920776	1.00	0.01	Population	13	16	significant
	MFA1t	–	0.721317	5.264013568			1.015710942			Mean	1	1.855567097	
	MFA1t	–	0.714089	5.17712916			0.998946274			Variance	0.000169503	0.010754066	
	MFA1t	–	0.715164	5.189959872			1.001422007			Observations	4	3	
14	MFA1t	NAB6	0.777645	5.993009967	5.993655244	0.114838131	1.156373502	1.16	0.02	Pooled Variance	0.004403328		
	MFA1t	NAB6	0.789318	6.156274837			1.187876064			Hypothesized Mean D	0		
	MFA1t	NAB6	0.771785	5.912688506			1.140875178			df	5		
	MFA1t	NAB6	0.771782	5.912647663			1.140867297			t Stat	-16.88127156		
15	MFA1t	PAP1	0.818429	6.583077987	6.510532358	0.152191885	1.270229315	1.26	0.03	P(T<=t) one-tail	6.66894E-06		
	MFA1t	PAP1	0.812938	6.500368843			1.254270278			t Critical one-tail	3.364929999		
	MFA1t	PAP1	0.799549	6.303024569			1.216191969			P(T<=t) two-tail	1.33379E-05		
	MFA1t	PAP1	0.823191	6.655658035			1.284233904			t Critical two-tail	4.032142984		
16	MFA1t	NAB6+PAP1	0.961349	9.148481201	9.616643835	0.537444007	1.76523338	1.86	0.10				
	MFA1t	NAB6+PAP1	0.977629	9.497930776			1.832660972						
	MFA1t	NAB6+PAP1	1.00875	10.20351953			1.968806938						
17	SCW4t	–	-0.0127184	0.971139458	0.966794932	0.027467844	1.004493741	1.00	0.03	Population	17	20	significant
	SCW4t	–	-0.0120794	0.972569397			1.005972792			Mean	1	1.550013572	
	SCW4t	–	-0.00235197	0.994599027			1.028759041			Variance	0.000807199	0.015670693	
	SCW4t	–	-0.0320442	0.928871847			0.960774427			Observations	4	3	
18	SCW4t	NAB6	0.0362583	1.087071976	0.9968581	0.069633379	1.12440802	1.03	0.07	Pooled Variance	0.006752596		
	SCW4t	NAB6	0.00614009	1.014238495			1.049073036			Hypothesized Mean D	0		
	SCW4t	NAB6	-0.031302	0.930460626			0.962417774			df	5		
	SCW4t	NAB6	-0.019696	0.955661301			0.988483979			t Stat	-8.763540773		
19	SCW4t	PAP1	0.104559	1.272210571	1.266113744	0.027616856	1.315905296	1.31	0.03	P(T<=t) one-tail	0.000160369		
	SCW4t	PAP1	0.111456	1.292575738			1.336969915			t Critical one-tail	3.364929999		
	SCW4t	PAP1	0.104632	1.272424434			1.316126503			P(T<=t) two-tail	0.000320737		
	SCW4t	PAP1	0.088931	1.227244233			1.269394566			t Critical two-tail	4.032142984		
20	SCW4t	NAB6+PAP1	0.185032	1.531200281	1.498545266	0.121025941	1.58379014	1.55	0.13				
	SCW4t	NAB6+PAP1	0.204091	1.599893228			1.654842381						
	SCW4t	NAB6+PAP1	0.134987	1.36454229			1.411408195						
21	TIR2t	–	1.01918	10.45153309	10.40796215	0.204871618	1.004186308	1.00	0.02	Population	21	24	significant
	TIR2t	–	1.0273	10.64878356			1.02313819			Mean	1	1.599249805	
	TIR2t	–	1.01615	10.37886827			0.997204622			Variance	0.000387465	0.010898442	
	TIR2t	–	1.00658	10.15266368			0.97547085			Observations	4	4	
22	TIR2t	NAB6	1.14536	13.97526333	14.4229188	0.444205576	1.342747325	1.39	0.04	Pooled Variance	0.005642953		
	TIR2t	NAB6	1.15998	14.45373207			1.388718739			Hypothesized Mean D	0		
	TIR2t	NAB6	1.15356	14.24163989			1.36834086			df	6		
	TIR2t	NAB6	1.1767	15.02103991			1.443225839			t Stat	-11.28157315		
23	TIR2t	PAP1	1.13522	13.65274567	14.31813364	0.456863774	1.311759734	1.38	0.04	P(T<=t) one-tail	1.45048E-05		
	TIR2t	PAP1	1.16587	14.65109215			1.407681152			t Critical one-tail	3.142668403		
	TIR2t	PAP1	1.16368	14.57739762			1.40060056			P(T<=t) two-tail	2.90095E-05		
	TIR2t	PAP1	1.1581	14.39129912			1.382720163			t Critical two-tail	3.707428021		
24	TIR2t	NAB6+PAP1	1.22216	16.67861562	16.64493144	1.0865455	1.602486191	1.60	0.10				
	TIR2t	NAB6+PAP1	1.17988	15.13143094			1.453832241						
	TIR2t	NAB6+PAP1	1.24707	17.66322495			1.697087739						
	TIR2t	NAB6+PAP1	1.23316	17.10645424			1.643593048						
25	YDR133Ct	–	-0.0269359	0.93986202	0.933412245	0.019077407	1.006909889	1.00	0.02	Population	25	28	significant
	YDR133Ct	–	-0.0431744	0.905368958			0.969956162			Mean	1	1.521224825	
	YDR133Ct	–	-0.0231342	0.948125441			1.015762806			Variance	0.000417726	0.000359542	
	YDR133Ct	–	-0.026737	0.94029256			1.007371144			Observations	4	4	
26	YDR133Ct	NAB6	0.0956442	1.246362002	1.250717599	0.011788318	1.335274964	1.34	0.01	Pooled Variance	0.000388634		
	YDR133Ct	NAB6	0.0941634	1.242119558			1.330729873			Hypothesized Mean D	0		
	YDR133Ct	NAB6	0.103171	1.268151092			1.358618444			df	6		
	YDR133Ct	NAB6	0.0956009	1.246237743			1.335141841			t Stat	-37.39121326		
27	YDR133Ct	PAP1	0.0518187	1.126726996	1.11256532	0.020190254	1.207105437	1.19	0.02	P(T<=t) one-tail	1.22116E-08		
	YDR133Ct	PAP1	0.0521917	1.127695117			1.208142622			t Critical one-tail	3.142668403		
	YDR133Ct	PAP1	0.0458779	1.111419213			1.190705628			P(T<=t) two-tail	2.44233E-08		
	YDR133Ct	PAP1	0.0351975	1.084419953			1.161780295			t Critical two-tail	3.707428021		
28	YDR133Ct	NAB6+PAP1	0.151138	1.416243729	1.419929878	0.01769899	1.517275713	1.52	0.02				
	YDR133Ct	NAB6+PAP1	0.155565	1.430754103			1.532821228						
	YDR133Ct	NAB6+PAP1	0.145062	1.396567721			1.496196058						
	YDR133Ct	NAB6+PAP1	0.157201	1.43615396			1.538606299						
29	RPL26Bt	–	0.885916	7.689816917	7.532230436	0.277438888	1.020921622	1.00	0.04	Population	29	32	no difference
	RPL26Bt	–	0.891667	7.792323965			1.034530745			Mean	1	0.949189098	
	RPL26Bt	–	0.855118	7.163380164			0.951030405			Variance	0.001356711	0.000349926	
	RPL26Bt	–	0.874099	7.483400696			0.993517227			Observations	4	4	
30	RPL26Bt	NAB6	0.827728	6.725553008	6.839748366	0.300324688	0.892903246	0.91	0.04	Pooled Variance	0.000853318		
	RPL26Bt	NAB6	0.827197	6.717334884			0.891812185			Hypothesized Mean D	0		
	RPL26Bt	NAB6	0.862465	7.285594572			0.967255932			df	6		
	RPL26Bt	NAB6	0.821547	6.630511002			0.880285203			t Stat	2.459893619		
31	RPL26Bt	PAP1	0.892182	7.801569828	7.843823524	0.070918704	1.035758252	1.04	0.01	P(T<=t) one-tail	0.024563281		
	RPL26Bt	PAP1	0.896882	7.886458095			1.047028256			t Critical one-tail	3.142668403		
	RPL26Bt	PAP1	0.890301	7.76785303			1.031281915			P(T<=t) two-tail	0.049126561		
	RPL26Bt	PAP1	0.898693	7.919413143			1.05140346			t Critical two-tail	3.707428021		
32	RPL26Bt	NAB6+PAP1	0.851732	7.107747643	7.14951101	0.140900149	0.943644476	0.95	0.02				
	RPL26Bt	NAB6+PAP1	0.860689	7.255861762			0.963308521						
	RPL26Bt	NAB6+PAP1	0.86131	7.266244379			0.964686946						
	RPL26Bt	NAB6+PAP1	0.84312	6.968190257			0.925116447						
33	RPL26At	–	0.706292	5.085012209	5.012665689	0.087693702	1.014432744	1.00	0.02	Population	33	36	no difference
	RPL26At	–	0.699948	5.011272279			0.999722022			Mean	1	1.019588028	
	RPL26At	–	0.704554	5.064703199			1.010381205			Variance	0.000306055	2.37179E-05	
	RPL26At	–	0.68928	4.889675067			0.975464029			Observations	4	4	

34	RPL26At	NAB6	0.676404	4.746833515	4.730494877	0.060883383	0.946967903	0.94	0.01	Pooled Variance	0.000164886		
	RPL26At	NAB6	0.675026	4.731795861			0.943967971			Hypothesized Mean D	0		
	RPL26At	NAB6	0.680771	4.794805557			0.956538069			df	6		
	RPL26At	NAB6	0.667317	4.648544577			0.927359785			t Stat	−2.157313658		
35	RPL26At	PAP1	0.695567	4.96097456	4.927037213	0.038742031	0.989687896	0.98	0.01	P(T<=t) one-tail	0.037175985		
	RPL26At	PAP1	0.69489	4.953247169			0.988146323			t Critical one-tail	3.142668403		
	RPL26At	PAP1	0.691757	4.917643027			0.981043487			P(T<=t) two-tail	0.074351969		
	RPL26At	PAP1	0.688089	4.876284096			0.972792602			t Critical two-tail	3.707428021		
36	RPL26At	NAB6+PAP1	0.705978	5.081337014	5.110853924	0.024412203	1.013699562	1.02	0.00				
	RPL26At	NAB6+PAP1	0.709875	5.127137919			1.022836598						
	RPL26At	NAB6+PAP1	0.710478	5.134261683			1.024257751						
	RPL26At	NAB6+PAP1	0.707628	5.100679082			1.017558201						
37	HSP150t	–	0.286585	1.934572456	2.070645074	0.199489966	0.934284914	1.00	0.10	Population	37	40	significant
	HSP150t	–	0.290096	1.950275657			0.941868639			Mean	1	1.464954503	
	HSP150t	–	0.30863	2.035307346			0.982933952			Variance	0.00928177	0.001547306	
	HSP150t	–	0.373358	2.362424838			1.140912495			Observations	4	4	
38	HSP150t	NAB6	0.398464	2.503018157	2.825900628	0.215615471	1.208810814	1.36	0.10	Pooled Variance	0.005414538		
	HSP150t	NAB6	0.467675	2.935452113			1.417651026			Hypothesized Mean D	0		
	HSP150t	NAB6	0.469489	2.947738814			1.423584781			df	6		
	HSP150t	NAB6	0.464995	2.917393426			1.40892974			t Stat	−8.936032896		
39	HSP150t	PAP1	0.388897	2.448482476	2.419537636	0.094314869	1.182473282	1.17	0.05	P(T<=t) one-tail	5.47944E-05		
	HSP150t	PAP1	0.368376	2.33547918			1.127899324			t Critical one-tail	3.142668403		
	HSP150t	PAP1	0.404834	2.540001657			1.226671673			P(T<=t) two-tail	0.000109589		
	HSP150t	PAP1	0.371841	2.354187232			1.136934215			t Critical two-tail	3.707428021		
40	HSP150t	NAB6+PAP1	0.497839	3.146581609	3.033400824	0.081450504	1.519614177	1.46	0.04				
	HSP150t	NAB6+PAP1	0.478804	3.011646541			1.454448461						
	HSP150t	NAB6+PAP1	0.470228	2.952758987			1.42600923						
	HSP150t	NAB6+PAP1	0.480383	3.022616161			1.459746143						
41	YLR285Ct	–	0.165882	1.465149698	1.493874224	0.027906878	0.980771791	1.00	0.02	Population	41	44	no difference
	YLR285Ct	–	0.171551	1.484400186			0.993658075			Mean	1	1.115487827	
	YLR285Ct	–	0.174459	1.494372958			1.000333853			Variance	0.000348975	0.005450098	
	YLR285Ct	–	0.185138	1.531574052			1.02523628			Observations	4	4	
42	YLR285Ct	NAB6	0.138214	1.374719205	1.38238579	0.00747795	0.920237583	0.93	0.01	Pooled Variance	0.002899537		
	YLR285Ct	NAB6	0.143608	1.391899888			0.931738339			Hypothesized Mean D	0		
	YLR285Ct	NAB6	0.141248	1.384356678			0.926688911			df	6		
	YLR285Ct	NAB6	0.139428	1.378567387			0.922813557			t Stat	−3.033101152		
43	YLR285Ct	PAP1	0.15991	1.445140259	1.445806508	0.024530143	0.967377465	0.97	0.02	P(T<=t) one-tail	0.01150299		
	YLR285Ct	PAP1	0.167143	1.46941003			0.983623659			t Critical one-tail	3.142668403		
	YLR285Ct	PAP1	0.163318	1.45652519			0.974998542			P(T<=t) two-tail	0.023005979		
	YLR285Ct	PAP1	0.149881	1.412150552			0.945294141			t Critical two-tail	3.707428021		
44	YLR285Ct	NAB6+PAP1	0.177246	1.503993641	1.666398511	0.110284939	1.006773942	1.12	0.07				
	YLR285Ct	NAB6+PAP1	0.232239	1.707021535			1.142680895						
	YLR285Ct	NAB6+PAP1	0.243081	1.750173082			1.171566558						
	YLR285Ct	NAB6+PAP1	0.231573	1.704405787			1.140929912						
45	SUC2t	–	0.619811	4.166880061	4.339947326	0.135285836	0.960122266	1.00	0.03	Population	45	48	significant
	SUC2t	–	0.634816	4.313362914			0.993874485			Mean	1	1.091067109	< 1.2-fold
	SUC2t	–	0.651882	4.486234801			1.033707201			Variance	0.000971708	0.00067172	
	SUC2t	–	0.642792	4.393311528			1.012296048			Observations	4	4	
46	SUC2t	NAB6	0.640131	4.366475221	4.385231842	0.07948118	1.006112492	1.01	0.02	Pooled Variance	0.000821714		
	SUC2t	NAB6	0.634763	4.312836556			0.993753203			Hypothesized Mean D	0		
	SUC2t	NAB6	0.639782	4.362967722			1.005304303			df	6		
	SUC2t	NAB6	0.653082	4.498647867			1.036567389			t Stat	−4.492790991		
47	SUC2t	PAP1	0.721975	5.271995125	5.396310974	0.092882709	1.214760164	1.24	0.02	P(T<=t) one-tail	0.002067788		
	SUC2t	PAP1	0.740096	5.496623623			1.26651851			t Critical one-tail	3.142668403		
	SUC2t	PAP1	0.733558	5.414495536			1.247594758			P(T<=t) two-tail	0.004135575		
	SUC2t	PAP1	0.732565	5.402129613			1.244745433			t Critical two-tail	3.707428021		
48	SUC2t	NAB6+PAP1	0.662619	4.598529745	4.73517378	0.11248085	1.059581926	1.09	0.03				
	SUC2t	NAB6+PAP1	0.673961	4.720206515			1.087618388						
	SUC2t	NAB6+PAP1	0.687751	4.872490488			1.122707287						
	SUC2t	NAB6+PAP1	0.676645	4.749468373			1.094360833						
49	GPB1t	–	−0.0795747	0.832578708	0.920419339	0.102148057	0.904564553	1.00	0.11	Population	49	52	significant
	GPB1t	–	−0.0781	0.835410636			0.907641333			Mean	1	1.266990243	
	GPB1t	–	−0.00869348	0.980181542			1.064929321			Variance	0.012316541	0.005500601	
	GPB1t	–	0.0143132	1.033506471			1.122864793			Observations	4	4	
50	GPB1t	NAB6	0.0269725	1.064075638	1.093729065	0.055114492	1.15607701	1.19	0.06	Pooled Variance	0.008908571		
	GPB1t	NAB6	0.0404907	1.097717783			1.1926279			Hypothesized Mean D	0		
	GPB1t	NAB6	0.0679496	1.169363678			1.270468393			df	6		
	GPB1t	NAB6	0.0186003	1.04375916			1.134003944			t Stat	−4.000427161		
51	GPB1t	PAP1	−0.0295762	0.934165448	0.998539662	0.043029664	1.014934616	1.08	0.05	P(T<=t) one-tail	0.003557757		
	GPB1t	PAP1	0.0069216	1.016065253			1.103915586			t Critical one-tail	3.142668403		
	GPB1t	PAP1	0.00869441	1.020221355			1.108431029			P(T<=t) two-tail	0.007115515		
	GPB1t	PAP1	0.0101755	1.023706592			1.112217604			t Critical two-tail	3.707428021		
52	GPB1t	NAB6+PAP1	0.0456166	1.110750711	1.166162322	0.068263855	1.206787672	1.27	0.07				
	GPB1t	NAB6+PAP1	0.0436713	1.105786542			1.201394294						
	GPB1t	NAB6+PAP1	0.0937023	1.240801472			1.348082792						
	GPB1t	NAB6+PAP1	0.081819	1.207310562			1.311696214						
53	YGP1t	–	0.704301	5.061753595	5.013121175	0.067409542	1.009701026	1.00	0.01	Population	53	56	significant
	YGP1t	–	0.694171	4.945053562			0.986422109			Mean	1	1.618222395	
	YGP1t	–	0.70583	5.079605678			1.013262098			Variance	0.000180812	0.021666396	
	YGP1t	–	0.696013	4.966071865			0.990614767			Observations	4	4	
54	YGP1t	NAB6	0.714384	5.180646985	5.877215946	0.496476954	1.033417467	1.17	0.10	Pooled Variance	0.010923604		
	YGP1t	NAB6	0.79527	6.241227307			1.244978346			Hypothesized Mean D	0		
	YGP1t	NAB6	0.794194	6.225783297			1.241897628			df	6		
	YGP1t	NAB6	0.767987	5.861206195			1.169173054			t Stat	−8.365208267		
55	YGP1t	PAP1	0.783758	6.077962273	6.138374656	0.123441527	1.212410804	1.22	0.02	P(T<=t) one-tail	7.93474E-05		
	YGP1t	PAP1	0.786325	6.113993885			1.219598264			t Critical one-tail	3.142668403		
	YGP1t	PAP1	0.781264	6.043158708			1.205468309			P(T<=t) two-tail	0.000158695		
	YGP1t	PAP1	0.800606	6.318383758			1.260369246			t Critical two-tail	3.707428021		
56	YGP1t	NAB6+PAP1	0.929917	8.509753889	8.112344955	0.737906853	1.697496149	1.62	0.15				
	YGP1t	NAB6+PAP1	0.845464	7.005901064			1.397512811						
	YGP1t	NAB6+PAP1	0.927667	8.465780423			1.688724475						
	YGP1t	NAB6+PAP1	0.927778	8.467944443			1.689156146						
57	SUI1t	–	0.923578	8.386446871	8.160039162	0.44773595	1.02774591	1.00	0.05	Population	57	60	no difference
	SUI1t	–	0.876753	7.529272232			0.922700502			Mean	1	1.067591574	
	SUI1t	–	0.931966	8.549997741			1.047788812			Variance	0.003010644	0.000131126	
	SUI1t	–	0.912458	8.174439803			1.001764776			Observations	4	4	
58	SUI1t	NAB6	0.932552	8.561542167	8.443618858	0.135628592	1.049203564	1.03	0.02	Pooled Variance	0.001570885		
	SUI1t	NAB6	0.916363	8.248272492			1.010812856			Hypothesized Mean D	0		
	SUI1t	NAB6	0.928115	8.474517874			1.038538873			df	6		
	SUI1t	NAB6	0.928915	8.490142899			1.040453695			t Stat	−2.411766979		
59	SUI1t	PAP1	0.921124	8.339192517	8.414928971	0.105823264	1.021954963	1.03	0.01	P(T<=t) one-tail	0.026223777		
	SUI1t	PAP1	0.927297	8.458571018			1.036584611			t Critical one-tail	3.142668403		
	SUI1t	PAP1	0.931649	8.543759209			1.04702429			P(T<=t) two-tail	0.052447554		
	SUI1t	PAP1	0.920029	8.31819314			1.019381522			t Critical two-tail	3.707428021		
60	SUI1t	NAB6+PAP1	0.936788	8.645457894	8.711589055	0.093440814	1.059487304	1.07	0.01				
	SUI1t	NAB6+PAP1	0.94678	8.846673515			1.084145963						
	SUI1t	NAB6+PAP1	0.93715	8.652667197			1.060370793						
	SUI1t	NAB6+PAP1	0.939597	8.701557613			1.066362237						
61	YDL159Wt	–	−0.146124	0.714292352	0.691075715	0.02471862	1.033594925	1.00	0.04	Population	61	64	significant
	YDL159Wt	–	−0.176582	0.665913777			0.963590186			Mean	1	1.127555641	< 1.2-fold
	YDL159Wt	–	−0.171407	0.673896188			0.975140891			Variance	0.001279373	0.000459411	
	YDL159Wt	–	−0.148619	0.710200543			1.027673998			Observations	4	4	
62	YDL159Wt	NAB6	−0.210925	0.615283119	0.620485868	0.019138936	0.89032664	0.90	0.03	Pooled Variance	0.000869392		
	YDL159Wt	NAB6	−0.214405	0.610372558			0.883220963			Hypothesized Mean D	0		
	YDL159Wt	NAB6	−0.216453	0.607501004			0.879065768			df	6		
	YDL159Wt	NAB6	−0.187898	0.648786792			0.938807106			t Stat	−6.117962164		
63	YDL159Wt	PAP1	−0.186318	0.651151432	0.696837174	0.042747123	0.942228786	1.01	0.06	P(T<=t) one-tail	0.000435327		
	YDL159Wt	PAP1	−0.150604	0.706961885			1.022987597			t Critical one-tail	3.142668403		
	YDL159Wt	PAP1	−0.12431	0.751086576			1.086836883			P(T<=t) two-tail	0.000870654		
	YDL159Wt	PAP1	−0.168675	0.678148804			0.981294508			t Critical two-tail	3.707428021		
64	YDL159Wt	NAB6+PAP1	−0.118372	0.761426522	0.779226321	0.014812438	1.101798986	1.13	0.02				
	YDL159Wt	NAB6+PAP1	−0.110965	0.774524215			1.120751602						
	YDL159Wt	NAB6+PAP1	−0.105281	0.784727731			1.135516288						
	YDL159Wt	NAB6+PAP1	−0.0989632	0.796226816			1.152155688						
65	DDR2t	–	0.648616	4.452623766	4.503457545	0.31083715	0.988712277	1.00	0.07	Population	65	68	significant
	DDR2t	–	0.647534	4.441544319			0.986252068			Mean	1	1.621311616	
	DDR2t	–	0.621967	4.187617442			0.929867196			Variance	0.004764021	0.009751121	
	DDR2t	–	0.693027	4.932044654			1.095168458			Observations	4	4	
66	DDR2t	NAB6	0.815879	6.544538095	6.477935603	0.065857732	1.453225223	1.44	0.01	Pooled Variance	0.007257571		
	DDR2t	NAB6	0.807051	6.412848794			1.423983402			Hypothesized Mean D	0		
	DDR2t	NAB6	0.811335	6.476419921			1.438099473			df	6		
67	DDR2t	PAP1	0.721932	5.271473165	5.510751843	0.59735109	1.170539105	1.22	0.13	t Stat	−10.31404011		
	DDR2t	PAP1	0.720064	5.248848044			1.165515161			P(T<=t) one-tail	2.4272E-05		
	DDR2t	PAP1	0.806267	6.401282591			1.421415108			t Critical one-tail	3.142668403		
	DDR2t	PAP1	0.709389	5.121403571			1.13721591			P(T<=t) two-tail	4.85441E-05		

68	DDR2t	NAB6+PAP1	0.877651	7.544856776	7.301508031	0.444706372	1.675347597	1.62	0.10
	DDR2t	NAB6+PAP1	0.860116	7.246294827			1.609051435		
	DDR2t	NAB6+PAP1	0.826128	6.700820742			1.487928036		
	DDR2t	NAB6+PAP1	0.887283	7.714059778			1.712919396		
69	YPR053Ct	–	0.585806	3.853062024	3.865897395	0.0120204	0.996679847	1.00	0.00
	YPR053Ct	–	0.587033	3.863963364			0.99949972		
	YPR053Ct	–	0.587088	3.864452736			0.999626307		
	YPR053Ct	–	0.589068	3.882111457			1.004194126		
70	YPR053Ct	NAB6	0.583941	3.836551214	3.814988234	0.067041159	0.99240896	0.99	0.02
	YPR053Ct	NAB6	0.570534	3.719923425			0.962240599		
	YPR053Ct	NAB6	0.5885	3.877037485			1.002881631		
	YPR053Ct	NAB6	0.582795	3.826440812			0.989793681		
71	YPR053Ct	PAP1	0.571074	3.72455164	3.722824073	0.006609763	0.963437789	0.96	0.00
	YPR053Ct	PAP1	0.569893	3.714437028			0.960821421		
	YPR053Ct	PAP1	0.570769	3.721936848			0.962761416		
	YPR053Ct	PAP1	0.571752	3.730370775			0.964943037		
72	YPR053Ct	NAB6+PAP1	0.522917	3.333626962	3.273330099	0.048723229	0.862316461	0.85	0.01
	YPR053Ct	NAB6+PAP1	0.507379	3.216466259			0.83201025		
	YPR053Ct	NAB6+PAP1	0.513285	3.260505972			0.843402098		
	YPR053Ct	NAB6+PAP1	0.516234	3.282721201			0.849148559		

t Critical two-tail	3.707428021	

Population	69	72	significant
Mean	1	0.846719342	< 1.2-fold
Variance	9.66801E-06	0.000158844	
Observations	4	4	
Pooled Variance	8.42561E-05		
Hypothesized Mean D	0		
df	6		
t Stat	23.61574275		
P(T<=t) one-tail	1.89175E-07		
t Critical one-tail	3.142668403		
P(T<=t) two-tail	3.78349E-07		
t Critical two-tail	3.707428021		
